# Predictable Components of ENSO Evolution in Real-time Multi-Model Predictions

**DOI:** 10.1038/srep35909

**Published:** 2016-10-24

**Authors:** Zhihai Zheng, Zeng-Zhen Hu, Michelle L’Heureux

**Affiliations:** 1 National Climate Center, and Laboratory for Climate Studies, China Meteorological Administration, Beijing, China; 2 Zhuhai Joint Innovative Center for Climate-Environment-Ecosystem, Zhuhai Key Laboratory of Dynamics Urban Climate and Ecology, Future Earth Research Institute, Beijing Normal University, Zhuhai, China; 3 Climate Prediction Center, NCEP/NOAA, College Park, MD, USA

## Abstract

The most predictable components of the El Niño-Southern Oscillation (ENSO) evolution in real-time multi-model predictions are identified by applying an empirical orthogonal function analysis of the model data that maximizes the signal-to-noise ratio (MSN EOF). The normalized Niño3.4 index is analyzed for nine 3-month overlapping seasons. In this sense, the first most predictable component (MSN EOF1) is the decaying phase of ENSO during the Northern Hemisphere spring, followed by persistence through autumn and winter. The second most predictable component of ENSO evolution, with lower prediction skill and smaller explained variance than MSN EOF1, corresponds to the growth during spring and then persistence in summer and autumn. This result suggests that decay phase of ENSO is more predictable than the growth phase. Also, the most predictable components and the forecast skills in dynamical and statistical models are similar overall, with some differences arising during spring season initial conditions. Finally, the reconstructed predictions, with only the first two MSN components, show higher skill than the model raw predictions. Therefore this method can be used as a diagnostic for model comparison and development, and it can provide a new perspective for the most predictable components of ENSO.

The El Niño–Southern Oscillation (ENSO), a coupled ocean–atmosphere phenomenon in the tropical Pacific Ocean, is the strongest known source of seasonal-to-interannual climate variability and predictability^1^. Predictions of the ENSO have received sustained attention since the 1980s. Due to improved observing and analysis/assimilation systems, improved physical parameterizations, higher spatial resolution, and better understanding of the tropical oceanic and atmospheric processes underlying the ENSO phenomenon^2^,^3^, the ability to predict ENSO has improved significantly in the past three decades^4^,^5^,^6^,^7^,^8^,^9^. Now, some major meteorological and climatological centers around the world routinely produce forecasts of the tropical Pacific Ocean with a focus on ENSO. Beginning in early 2002, real-time predictions of the sea surface temperature (SST) in the Niño3.4 region (5°S–5°N, 120°–170°W) have been collected from a large number of models, including both dynamical and statistical models^10^,^11^,^12^,^13^,^14^,^15^,^16^, each month by the International Research Institute for Climate and Society (IRI) at Columbia University.

These real-time predictions from various models have been a critical tool in national and international operational ENSO outlooks. Nevertheless, due to model error and the dependence of ENSO forecast skill on the season, decade, and ENSO phase^6^,^17^,^18^, predicting ENSO evolution is still a challenge^19^, despite upgrades in models and improvements in the observing systems. Climate variability consists of predictable signal and unpredictable noise. Thus, increasing the skill of forecast model involves isolating and then improving the physics behind the predictable part of ENSO variability.

Combining predictions, such as equally weighting and averaging the forecasts from multiple models, is a way to reduce the error arising from individual models, to suppress the unpredictable noise, and to isolate the predictable signal^20^,^21^. In this study, we propose a novel way to isolate the predictable signal by identifying the most predictable components of ENSO *evolution*. To do this, we compute an Empirical Orthogonal Function with a maximized signal-to-noise ratio (MSN EOF) to identify the predictable components from real-time ENSO predictions of the ensemble mean of 20 models collected by IRI (see Data and Methods; Table 1). In this paper, predictable components refer to predicting the models themselves rather than actual behavior in the real world. This is because signal-to-noise cannot be estimated using the observational data alone^22^. Thus, the MSN EOF modes can provide a best estimate of the most predictable components. We also compare the predictable components between the dynamical and statistical models.

The real-time multi-model predictions of Niño3.4 index, issued each month from February 2002 through January 2016, were collected by International Research Institute for Climate and Society (IRI). The 20 real-time model predictions used include 11 dynamical models and 9 statistical models (Table 1). Eight dynamical models had insufficient lead times (less than nine 3-month overlapping seasons) and were excluded. The forecast data from each model and corresponding observations are based on 3-month running mean SST anomalies averaged in the Niño3.4 region. Forecasts made from each start time are run out for nine 3-month overlapping seasons. For example, for predictions initialized in the end of December, the first target season is January-March and the last one is September-November. To suppress the impact due to the varying amplitudes and variances among the models, the normalized Niño3.4 index is used to isolate the predictable components.

Figure 1 shows the leading MSN EOF mode (MSN EOF1) of the multi-model ensemble mean ENSO (normalized Niño3.4 index) evolution and the associated principal component (PC) for initial conditions (ICs) in January (a), April (b), July (c), and October (d) with 0–8 month leads, respectively. For the January IC, MSN EOF1 (bars in Fig. 1a) shows a decline for target seasons in January-February-March (JFM) through September-October-November (SON) that corresponds to a decay of El Niño or La Niña. By comparing with the corresponding observational composite (red curve in Fig. 1a), which is defined as the difference between the mean of three maximum positive PC1 years minus the mean of three maximum negative PC1 years in observations, the decline in MSN EOF1 is associated with decay of warm or cold events, although the decay speed is not same, particularly for the longer leads (Fig. 1a). A similar ENSO decay pattern is evident in MSN EOF1 for IC in November and December (Supplementary Fig. S1).

For IC in April (Fig. 1b), MSN EOF1 decreases sharply before the target season of July-August-September (JAS) and then is relatively constant during autumn and winter. A similar evolution in MSN EOF1 is also seen for IC in February and March (Supplementary Fig. S1). MSN EOF1 for IC in July (Fig. 1c), May, and June (Supplementary Fig. S1) mostly reflects persistence, though with a slow decay into the spring. For IC in October (Fig. 1d), as well as in August and September (Supplementary Fig. S1), the MSN EOF1 shows that the anomaly largely persists through the autumn and winter and then decreases sharply into the spring and summer. Thus, the most predictable evolution of the Niño3.4 anomaly is a decline for forecast targets in spring through autumn and is then relatively constant for the forecast targets in autumn to the following spring.

It is difficult to meaningfully apply significance tests to the real-time prediction period because it is so short (less than five samples of El Niño or La Niña events). So, in order to cross-check whether the most predictable components of the Niño3.4 evolution in MSN EOF1 exist in the real world, we examine the similarity between the time series (PC1) computed by projecting the ensemble mean of the model forecasts onto MSN EOF1 (red line in the right panels of Fig. 1) and by projecting the observational data onto MSN EOF1 (blue line in the right panels of Fig. 1). The correlations between the two PC1 time series are greater than 0.8 for all panels, corroborating the significance of the ENSO evolution pattern identified in MSN EOF1. In addition, the composite of the observed Niño3.4 index evolution is computed based on PC1 for the mean of years with the three most positive PC1 values minus that with the three most negative PC1 values, representing the Niño3.4 evolution in extreme years of PC1. Overall, the red line shown in the left panels of Fig. 1 nicely follow the trajectory of MSN EOF1 evolution (blue bars), which provides further evidence that the MSN EOF1 is robust. The rightmost columns in Table 2 show the correlations between the observed and forecast PCs for all initial months, which range from 0.80 to 0.98, with a minimum for ICs in April and May. The smaller correlations during spring are likely associated with the spring predictability barrier, a period of less skill in ENSO forecasts^23^,^24^,^25^.

Table 2 also shows the fractions of variance explained by the most predictable components of the leading MSN EOFs relative to the variability of the Niño3.4 index within the model and in observational data, respectively. The explained variances of ensemble means associated with the specified MSN EOF mode are calculated from the ensemble mean data projected onto the normalized MSN EOFs, following Venzke *et al*.^26^, Hu and Huang^27^. Then the fractions are calculated as the ratio of the explained variance to the total variances of the ensemble mean of all models and to the observations, respectively. As expected, MSN EOF1 explains more variance within the model than in the observations, which is likely due to the fact the EOFs are derived from the model data and also because the model data is based on ensemble means, which suppresses the noise. MSN EOF1 explains roughly 50% to 90% of total variability for the ensemble mean of the models (with highest percentages for IC during autumn and winter and lowest percentages during spring and summer).

As shown in Fig. 1, there is a sharp decrease of MSN EOF1 during spring, followed by persistence in autumn and winter. That may be due to the fact that Niño3.4 is less persistent during spring^24^. To evaluate the relationship between predictability and persistence, Fig. 2 shows the lag auto-correlations of the observed Niño3.4 index for December-February (DJF), March-May (MAM), June-August (JJA), and September-November (SON). Overall, the auto-correlation of Niño3.4 index is the lowest during MAM and the highest in JJA and SON (Fig. 2 and Supplementary Fig. S2). The similarity between the lag auto-correlation (Fig. 2) and evolution of MSN EOF1 (left panels of Fig. 1) may imply a connection between ENSO predictability and its persistence.

The most noticeable feature of the second most predictable mode of ENSO evolution (MSN EOF2; Fig. 3 and Supplementary Fig. S3) is the growth in spring and the persistence in summer and autumn. Compared to MSN EOF1, the maximum correlation coefficients associated with MSN EOF2 are much lower (right column of Table 2). The correlations associated with MSN EOF1 are around 0.5 for ICs in April, May and July. For MSN EOF2, the correlations are even below zero for ICs in December, February, June and October. Interestingly, the correlation associated with PC2 reaches maximum for IC during spring (April and May) when the correlation with PC1 is at a minimum. In addition, the variances explained by MSN EOF2 are much smaller than that of MSN EOF1 (~5% to 20% of the total variance in models and ~1% to 14% in observations). Overall, the lower correlations and smaller fractions of explained variance in MSN EOF2 suggest that this mode may be less robust than MSN EOF1. The results here indicate that ENSO decay is more predictable than ENSO growth, which might be due to the fact that ENSO growth is triggered by atmospheric noise (such as westerly or easterly wind bursts) and the decay seems associated with large-scale air-sea interaction, which is relatively more predictable^1^,^5^,^6^. Regardless of the exact driver, these results motivate future work to isolate the causes of differing predictability.

Because the skill of statistical models often rivals the skill of dynamical models in ENSO prediction^28^,^29^, we compare ability of MSN EOF1 of these two sets of models in predicting ENSO evolution. Barnston *et al*.^28^ used these same model predictions and noticed the skill of the dynamical models exceeds that of statistical models for start times between March and May. Beyond the spring, the statistical and dynamical models have comparable skill. Figure 4 shows strong similarity between the leading predictable component of the dynamical models and statistical models. However, some differences between the two types of models occur for IC in spring. For example, the decay of the dynamical models is faster than that of the statistical models (Fig. 4b,f). The statistical models favor persistence after summer, while the dynamical models favor a phase transition for targets in the late summer or early fall. Another noticeable difference, for the July IC, is that the correlation with the observations is higher for the dynamical models than for the statistical models (Fig. 4). We further compare the spread using all of the models, the dynamical models only, and the statistical models only (Supplementary Fig. S6). The overall spread among dynamical models are larger than the spread among statistical models despite the fact they have a similar evolution. The inter-model spread strongly depends on the target season. The spread tends to persist for target seasons in autumn and winter, while increasing sharply for target seasons between MAM and JJA.

To enable comparison of forecast skill among various models for ENSO evolution, we reconstruct the forecasts by projecting the forecasts onto MSN EOF1 and EOF2, that largely filters out the unpredictable noise^30^,^31^,^32^,^33^. Because we focus on ENSO *evolution*, the squared error skill score (SESS) of the *tendency* between two adjacent seasons which represents the evolution is used to evaluate the skill. The SESS difference, defined as the SESS of reconstructed prediction minus the SESS of raw predictions, is positive nearly everywhere for all target seasons except at shorter lead times. That suggests that the reconstruction based on the predictable components only increases the skill of tendency at longer lead times (Fig. 5a). Thus, this method can isolate the predictable components at these lead times, which can be used to improve the model forecasts. Additionally, the improvement is most significant for targets during winter and less so during spring. Figure 5b,c show similar skill improvements at longer lead times associated with the predictable components of the dynamical and statistical models. Nevertheless, at shorter lead times, the dynamical models are slightly less skillful than their respective raw predictions, while the decrease in skill in the statistical models is smaller relative to the raw predictions. Overall, the reconstructed forecasts based on either dynamical or statistical models are more skillful than the raw forecasts, and the skill improvement at long leads is more significant for the dynamical models than for the statistical models. The overall skill difference is small between the reconstructions based on MSN EOF1 only and based on first two MSN components for all the models and the statistical models only, implying that compared with MSN EOF1, MSN EOF2 has a relatively smaller contribution to the prediction skill.

In this study, the predictable components of real-time multi-model ENSO evolution are identified with an EOF analysis that maximizes the signal-to-noise ratio. In particular, the first most predictable component largely resembles the lag auto-correlations of the Niño3.4 index. This component shows that the most predictable features resemble the decaying phase of ENSO through spring, followed by persistence during autumn and winter. The most significant difference between the dynamical models and statistical models presents for forecasts made with spring ICs. The second most predictable component, with relatively lower prediction skills and smaller explained variances, is associated with the growth in spring and then persistence during summer and autumn. The overall results indicate that ENSO decay is more predictable and its prediction is more reliable than the growth of ENSO. By isolating the predictable signal, this method provides a way to isolate the components of ENSO prediction that are most predictable, which can be used to compare and improve the forecasts, particularly at the longest lead times when the unpredictable noise is larger.

We should point out that the ‘most predictable components’ are calculated using only the models and are independent of the observations. Because the observations represent a single realization, they cannot be used to estimate the spread or noise of the system. Although models have errors and biases, these models are our only available tools which can be used to evaluate predictability.

Clearly, there are a few caveats and areas for future study. First, while the multi-model data used here represents the skill in real-time ENSO updates, the length of the 14-year period (2002–2015) is short. This may affect the robustness of the MSN EOF modes, particularly MSN EOF2, which captures less variability. ENSO skill can vary from decade to decade, and the data period analyzed here does not include the distinct shift in ENSO variability around 1999–2000^34^. It would be worthwhile to examine a longer hindcast record to see if the results vary. Second, the models used in the IRI multi-model plume are initialized in different ways, with different analysis or reanalysis products, which can account for considerable spread among the models^35^. For example, the NCEP CFSv2 uses the Climate Forecast Reanalysis, while ECMWF model uses its own data assimilation system. Therefore, future work could examine the dependence of the predictable components on the initialization strategy used by the various modeling systems.

## Data and Methods

The Reynolds–Smith^36^ version 2 optimal interpolation (OI) SST data averaged over the Niño3.4 region are used to verify the model predictions. The observed anomalies are based on monthly means during 2002–2015. Details of the real-time ENSO multi-model forecasts can be found in Barnston *et al*.^28^ and Tippett *et al*.^17^. Here, a description of the models is also provided in Table 1.

The empirical orthogonal function (EOF) analysis with maximized signal-to-noise ratio (MSN EOF) is employed, which is a method to derive components that optimize the signal-to-noise ratio from all ensemble members. This approach was developed by Allen and Smith^37^ and discussed in Venzke *et al*.^26^. The MSN EOF has been successfully used in identifying the most predictable components^38^,^39^,^40^,^41^. This method minimizes the effects of noise in a moderate ensemble size by maximizing the ratios of the variances of ensemble mean (the signal) to the deviations or spreads among the ensemble members (the noise). In this study, the multi-model ensemble average is treated as the signal, while the departure of ensemble mean of individual model from the multi-model ensemble average as spread (noise). By definition, the leading MSN EOF mode represents the most predictable component in the multi-model predictions. In this work, X_ijk_ is referred to the prediction with lead month *i* at a certain IC month of year *j* from model *k*. The average for all models 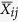 is referred to as “signal” and 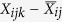 as “noise”. Then each IC month, we have matrixes of 9 (month lead) x 14 (year: 2002~2015) for the “signal” and 9 × 14 × 20 (available models) for the noise. Using MSN EOF technique to de-composite these two matrixes, we can get total 9 MSN EOF modes. For example, for first mode, MSN EOF1 is the evolution of Niño3.4 (analogy to the spatial pattern in conventional EOF), and MSN PC1 is the corresponding time series. The first two modes are analyzed in this work.

The squared error skill score (hereinafter SESS) of tendency is used to measure skill^17^,^30^, and defined as


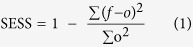


where f and o represent the tendency of forecast (model average or reconstruction based on MSN EOF modes) and observed anomalies, respectively, where tendency means the differences between successive values, e.g. Feb-Mar-Apr minus Jan-Feb-Mar etc. The SESS is equal to one for a perfect prediction, and is negative if the mean square error of a prediction is larger than the variance of the observation.

## Additional Information

**How to cite this article**: Zheng, Z. *et al*. Predictable Components of ENSO Evolution in Real-time Multi-Model Predictions. *Sci. Rep*. **6**, 35909; doi: 10.1038/srep35909 (2016).

## Supplementary Material

Supplementary Information

## Figures and Tables

**Figure 1:**
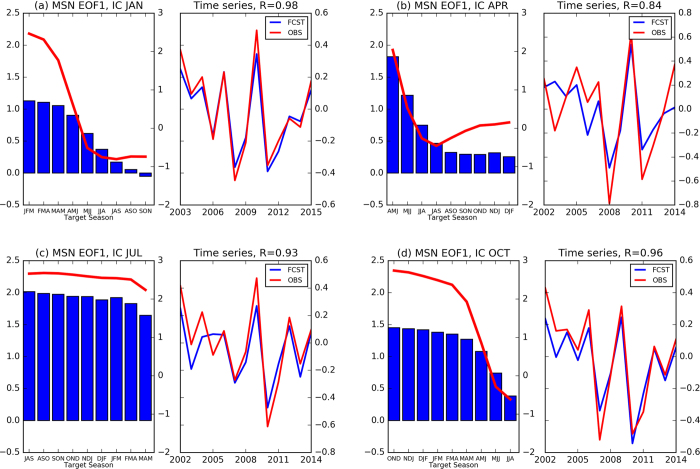
First MSN EOF mode (MSN EOF1) and corresponding PC1 of the model forecasts for ICs in (**a**) January, (**b**) April, (**c**) July, and (**d**) October, respectively. In the left panel of each pair, the bars represent MSN EOF1, the red line is the composite of the observed evolution, which is defined as the differences between the mean of three maximum positive PC1 years minus the mean of three maximum negative PC1 years in observation. The y-axis (on the right) in the bar-plot of each panel is for the scale of the observational composite (red curve). The red and blue lines in right panels of each pair are the corresponding PC1, which the projections of mean forecasts for all model means and observations onto the corresponding MSN EOF1, respectively. Figure is generated by Python (https://www.python.org/).

**Figure 2:**
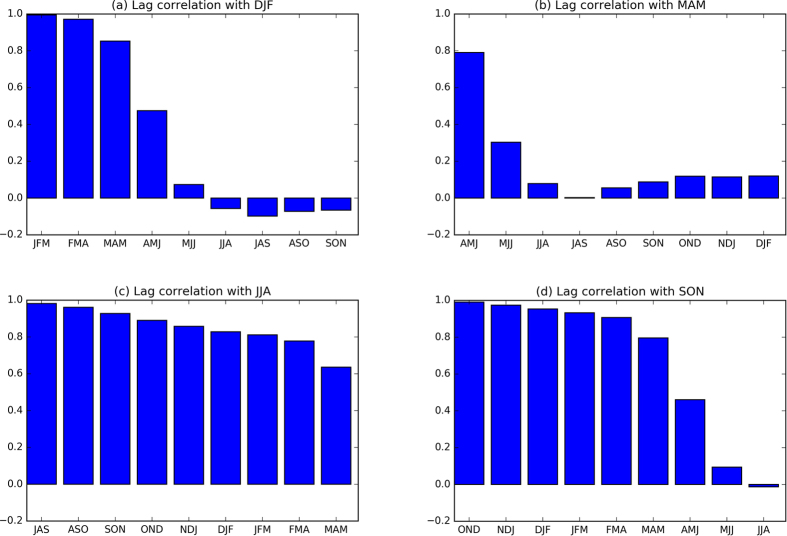
Lag auto-correlations of Niño3.4 index of OI SST for 2002–2015 period for (**a**) DJF, (**b**) MAM, (**c**) JJA, and (**d**) SON, respectively. Figure is generated by Python (https://www.python.org/).

**Figure 3:**
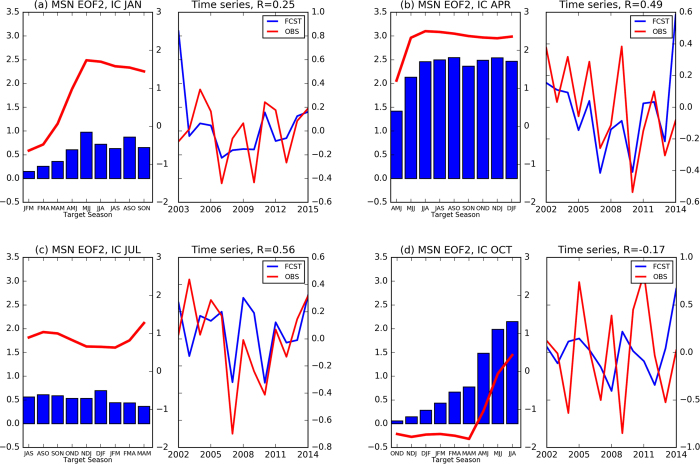
Same as Fig. 1, but for the second MSN EOF mode (MSN EOF2 and PC2). Figure is generated by Python (https://www.python.org/).

**Figure 4:**
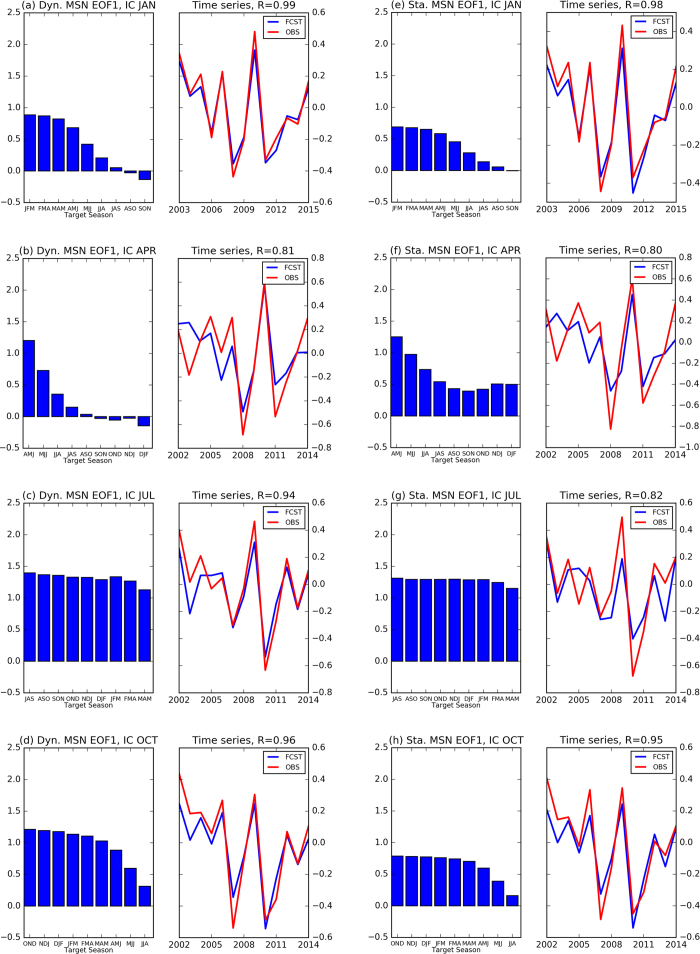
Same as Fig. 1, but for MSN EOF1 and PC1 of dynamical (**a**–**d**) and statistical (**e**–**h**) models for ICs in January, April, July, and October, respectively. Figure is generated by Python (https://www.python.org/).

**Figure 5:**
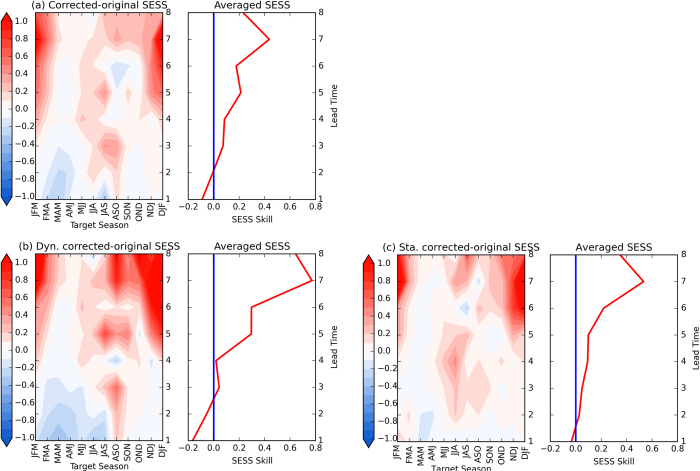
SESS skill score of reconstructed tendency between two adjacent target seasons based on the first two MSN EOF modes minus that of the original raw forecast of normalized Niño3.4 index for (**a**) all the models, (**b**) the dynamical models only, and (**c**) the statistical models only. Figure is generated by Python (https://www.python.org/).

**Table 1:** Information about model name and type.

Dynamical models	Model type
COLA Anomaly	Anomaly coupled
COLA CCSM3	Fully coupled
Univ. Maryland ESSIC	Intermediate coupled
GFDL CM2.1	Fully coupled
GFDL FLOR	Fully coupled
Japan Frontier FRCGC	Fully coupled
Korea Met. Agency SNU	Intermediate coupled
Lamont–Doherty Australia	Intermediate coupled
Scripps Hybrid Coupled Model (HCM)	Comprehensive ocean, statistical atmosphere
Japan Frontier SINTEX	Fully coupled
CMC CANSIP	Fully coupled
**Statistical Models**	**Method and predictors**
NOAA/NCEP/CPC Markov	Markov: Preferred persistence and transitions in SST and sea level height fields
NOAA/ESRL Linear Inverse Model (LIM)	Refined POP: Preferred persistence and transitions within SST field; optimal growth structures
NOAA/NCEP/CPC Constructed Analogue (CA)	Analogue-construction of current global SSTs
NOAA/NCEP/CPC Canonical Correlation Analysis (CCA)	Uses SLP, tropical Pacific SST and subsurface temperature (subsurface not used beginning in 2010)
NOAA/AOML CLIPER	Multiple regression from tropical Pacific SSTs
UBC Neural Network (NN)	Uses sea level pressure and Pacific SST
Florida State Univ. multiple regression	Uses tropical Pacific SST, heat content, winds
UCLA TDC multilevel regression	Uses 60°N–30°S Pacific SST field
Univ. Brasilia Columbia water center	Based on nonlinear method of dimensionality reduction and on a regularized least squares regression

**Table 2:** Percentages of explained variance relative to total variance and PC correlation of first two MSN EOFs in models for different IC month and its counterpart of observation.

IC Month	Explained variance in models (%)		Explained variance in observation (%)		Correlation	
	EOF1	EOF2	EOF1	EOF2	PC1	PC2
Dec	90.2	5.2	40.0	1.4	0.98	−0.30
Jan	89.3	4.9	49.6	2.1	0.98	0.25
Feb	81.2	9.5	35.6	2.3	0.95	−0.30
Mar	77.2	9.6	46.2	6.6	0.96	0.22
Apr	61.1	17.3	43.7	6.4	0.84	0.49
May	49.1	17.9	27.1	13.6	0.80	0.61
Jun	60.9	13.6	28.2	6.3	0.92	−0.04
Jul	72.2	11.1	31.6	5.4	0.93	0.56
Aug	76.0	8.7	27.8	6.3	0.91	0.28
Sep	80.0	8.2	26.3	4.6	0.97	0.14
Oct	88.0	5.7	35.3	6.8	0.96	−0.17
Nov	87.8	4.2	33.4	8.2	0.97	0.12
