# Supplementary material for: Predictable Components of ENSO Evolution in Real-time Multi-Model Predictions
Source: Sci Rep. 2016 Oct 24;6:35909. doi: 10.1038/srep35909 (PMC5075933; doi:10.1038/srep35909)
Supplement: Supplementary Information [file srep35909-s1.doc]

**Supplementary Material of**

**“Predictable Components of ENSO Evolution in Real-time Multi-Model Predictions”**

**by**

### Zhihai Zheng, Zeng-Zhen Hu, and Michelle L'Heureux


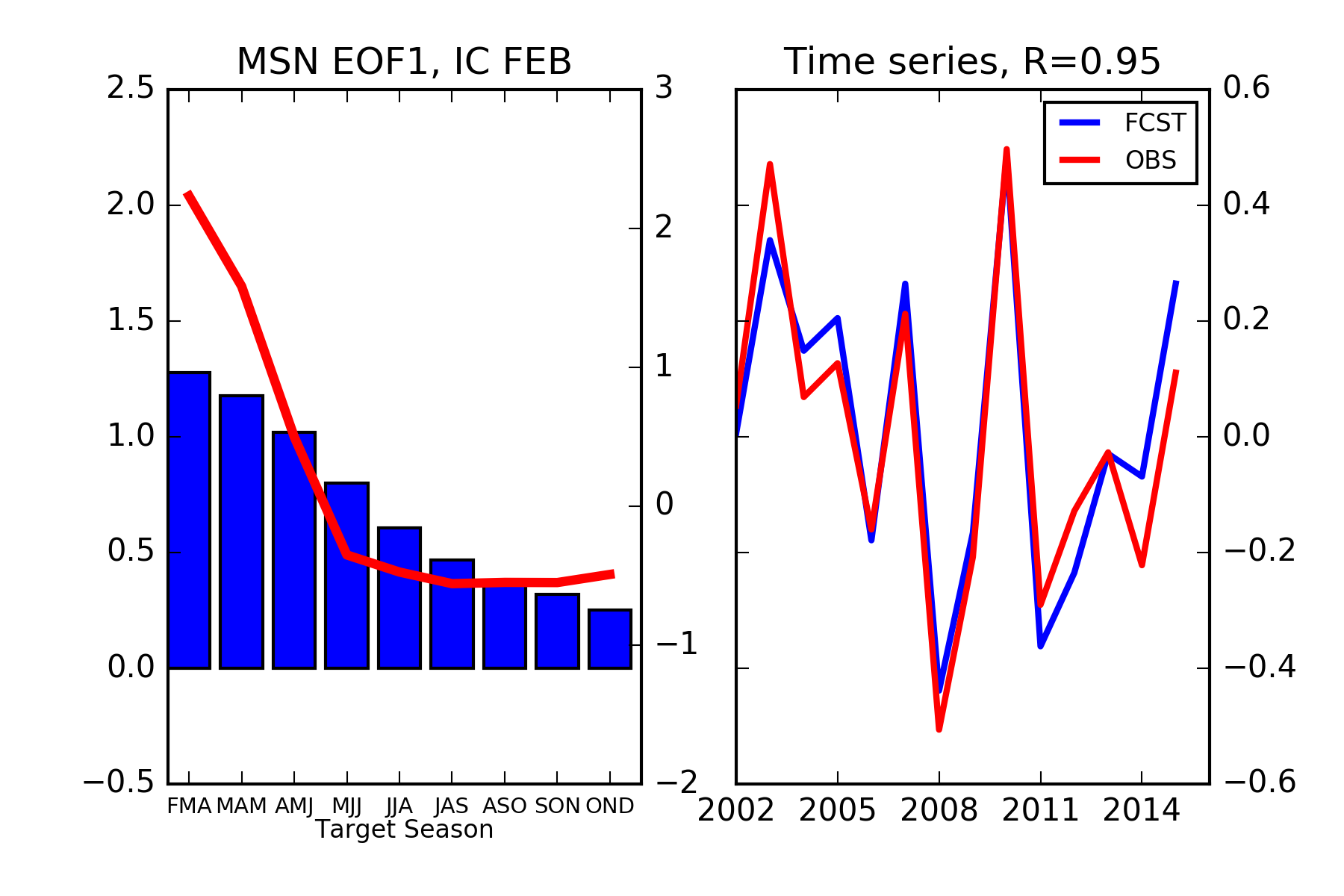

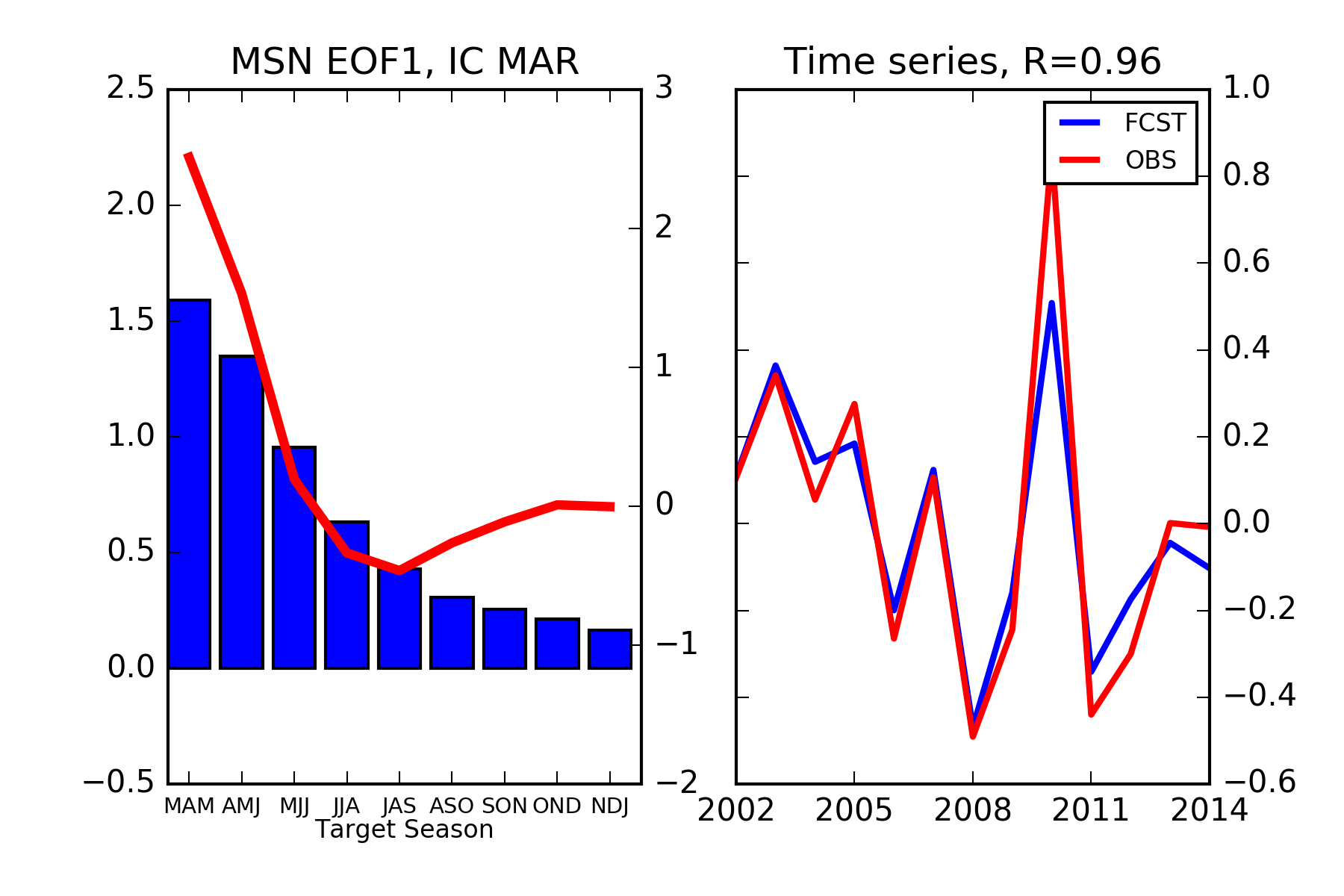

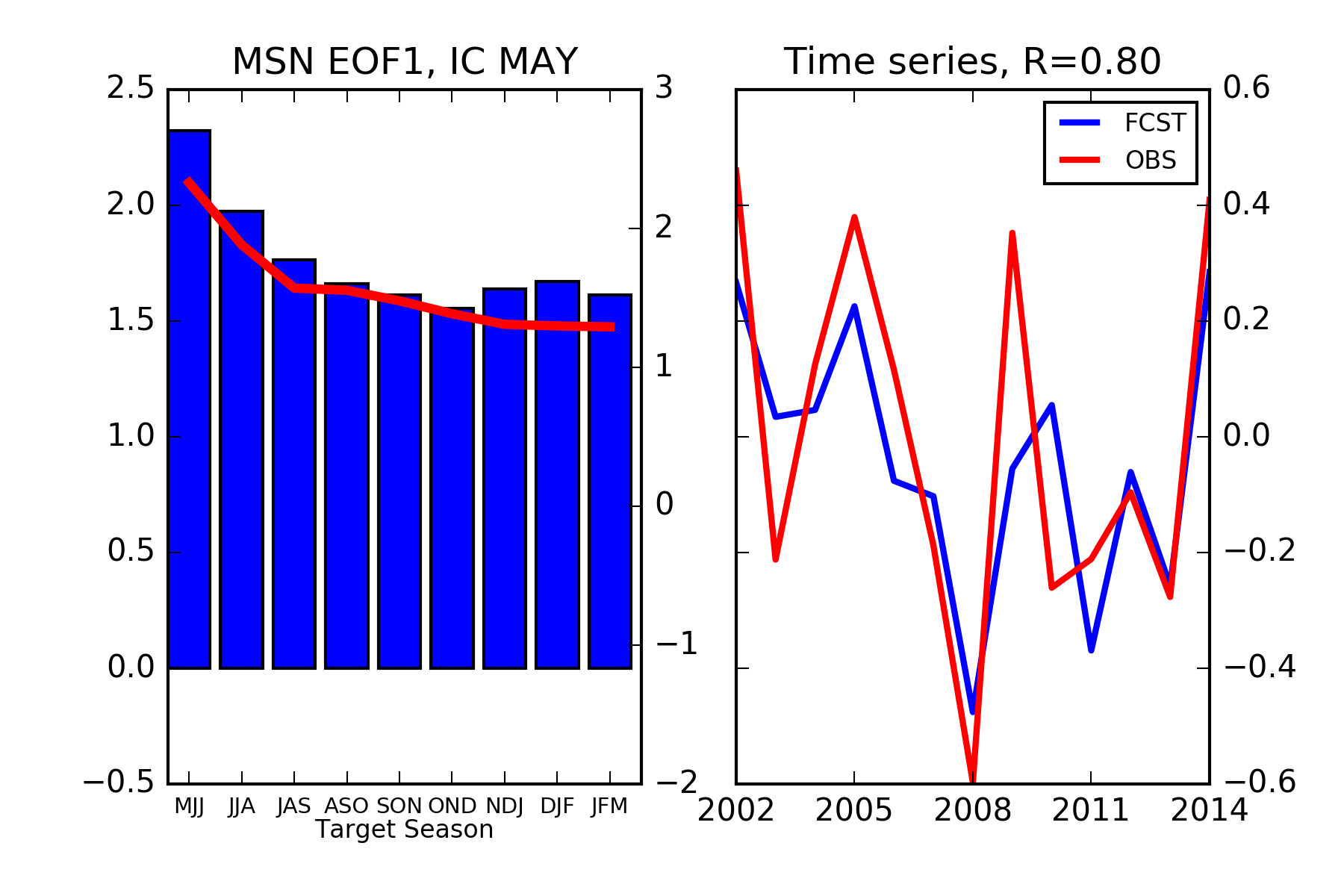

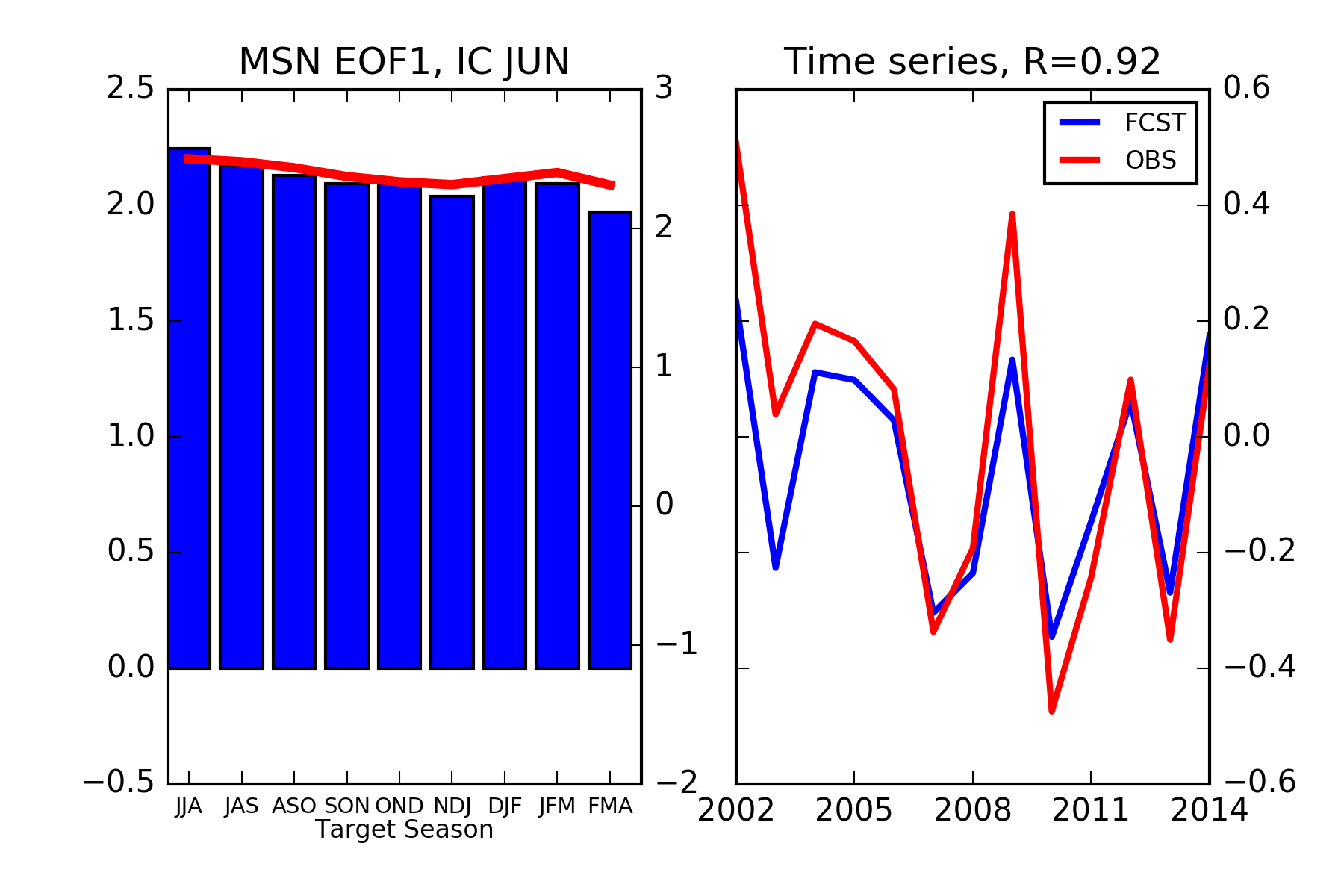

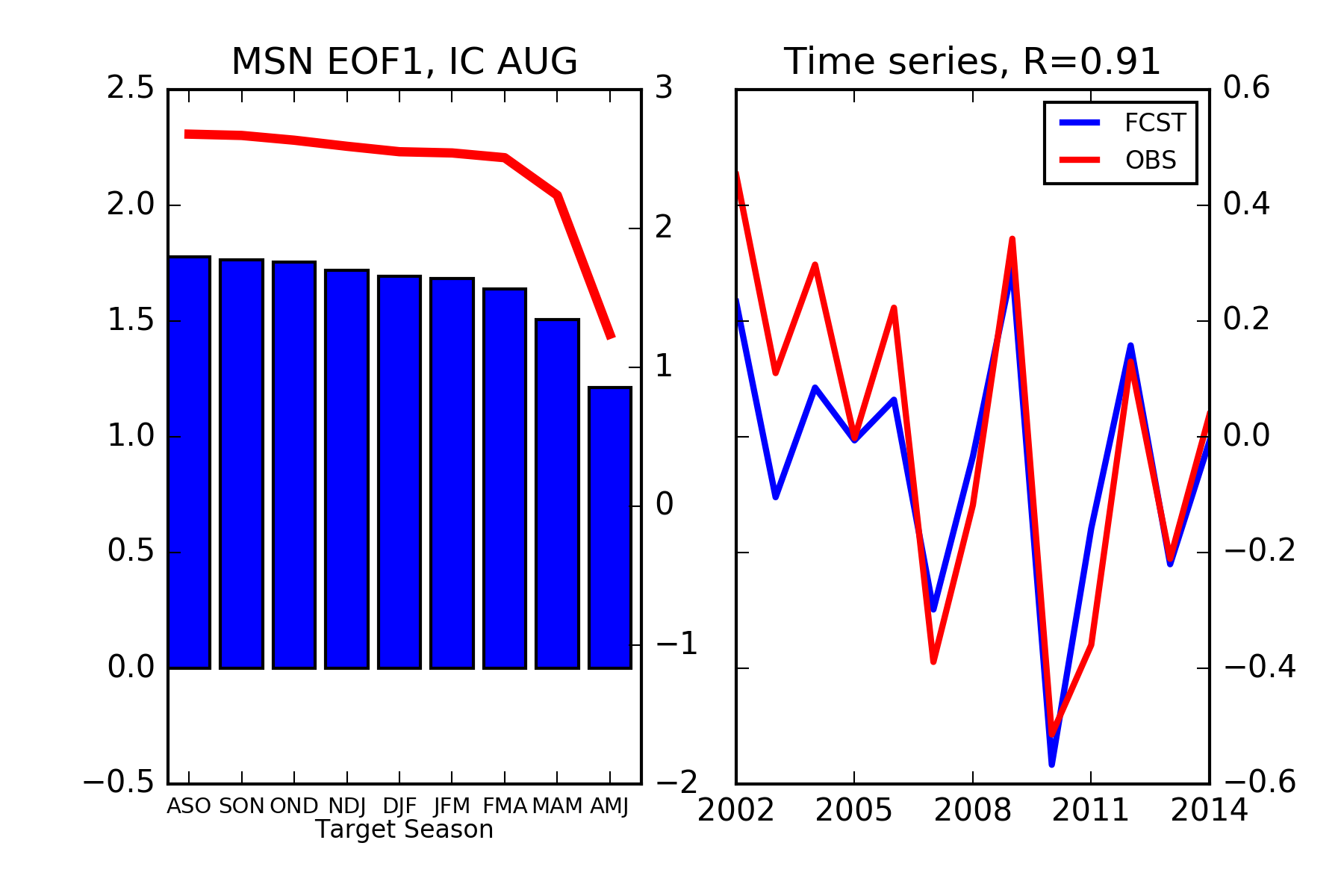

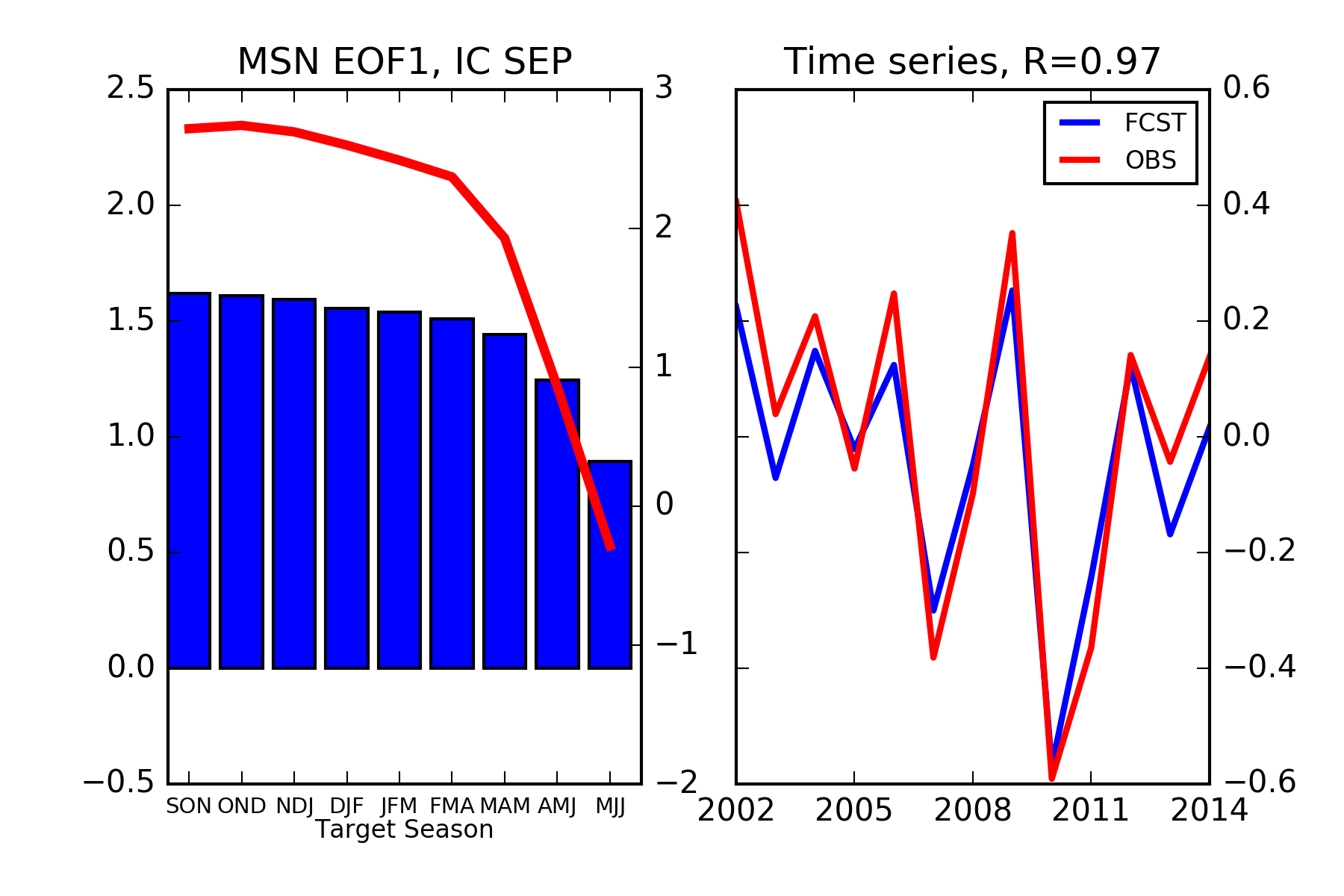

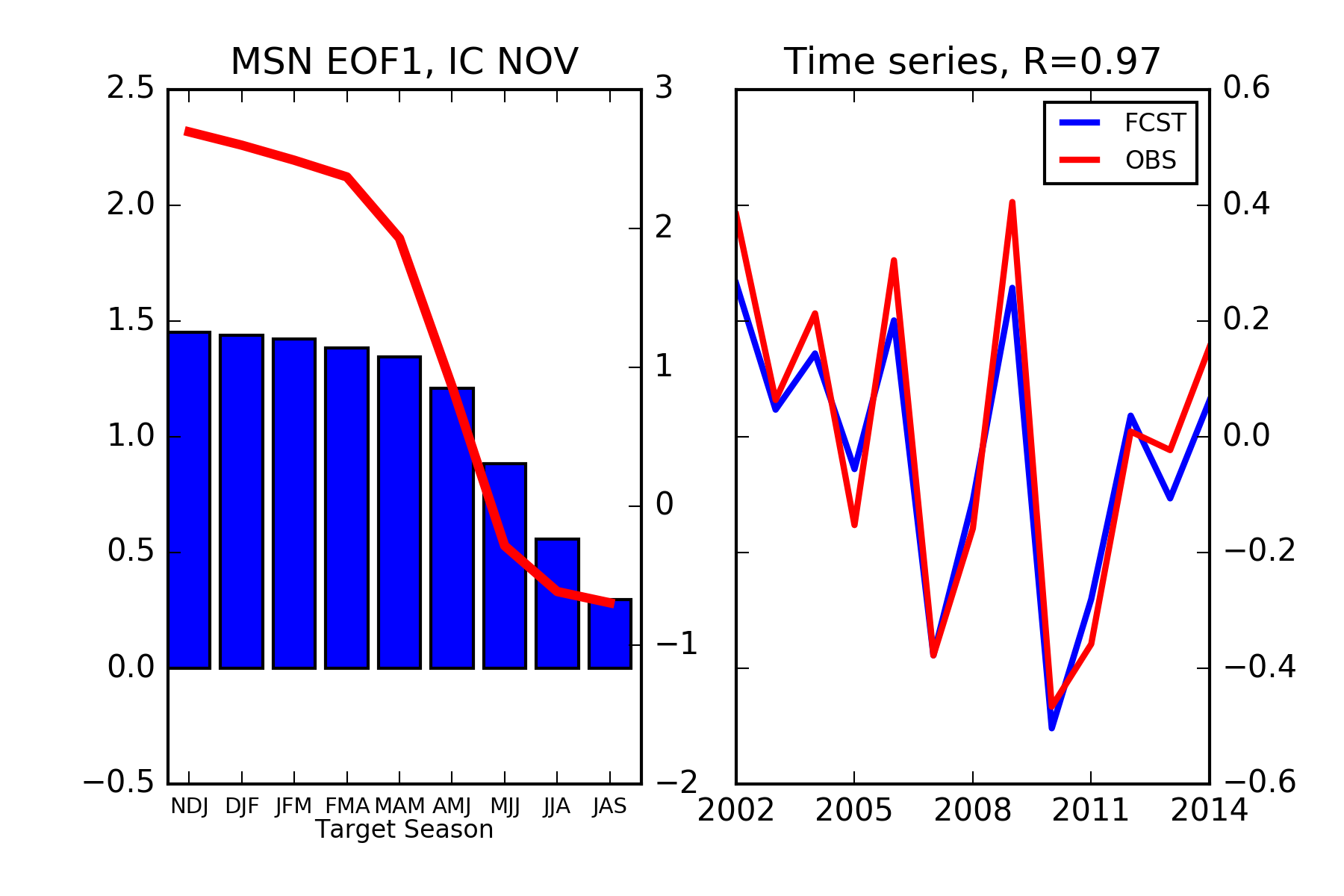

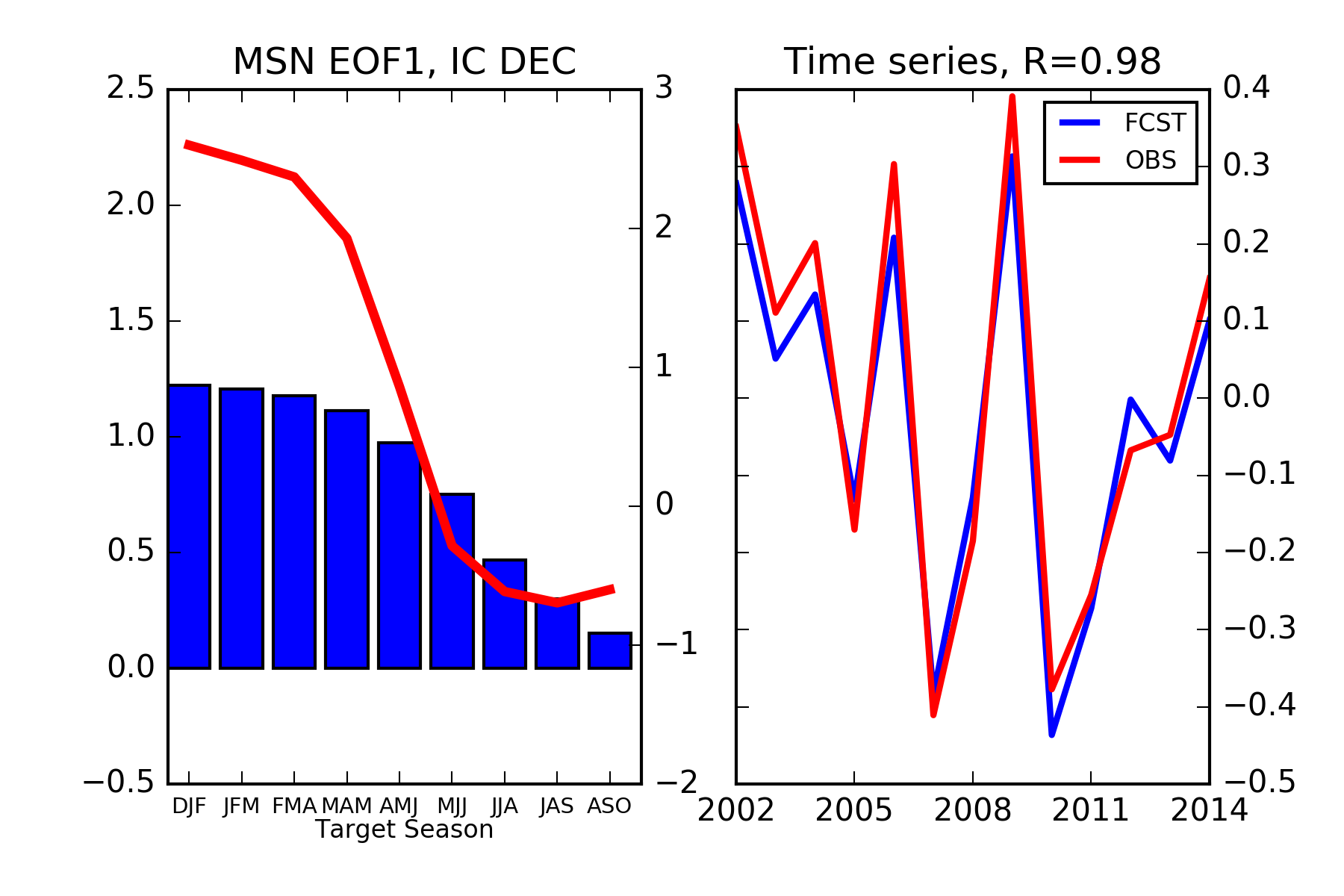


**Supplementary Figure 1** Same as Fig. 1, but for ICs in all other 8 months. Figure is generated by Python (https://www.python.org/)

**
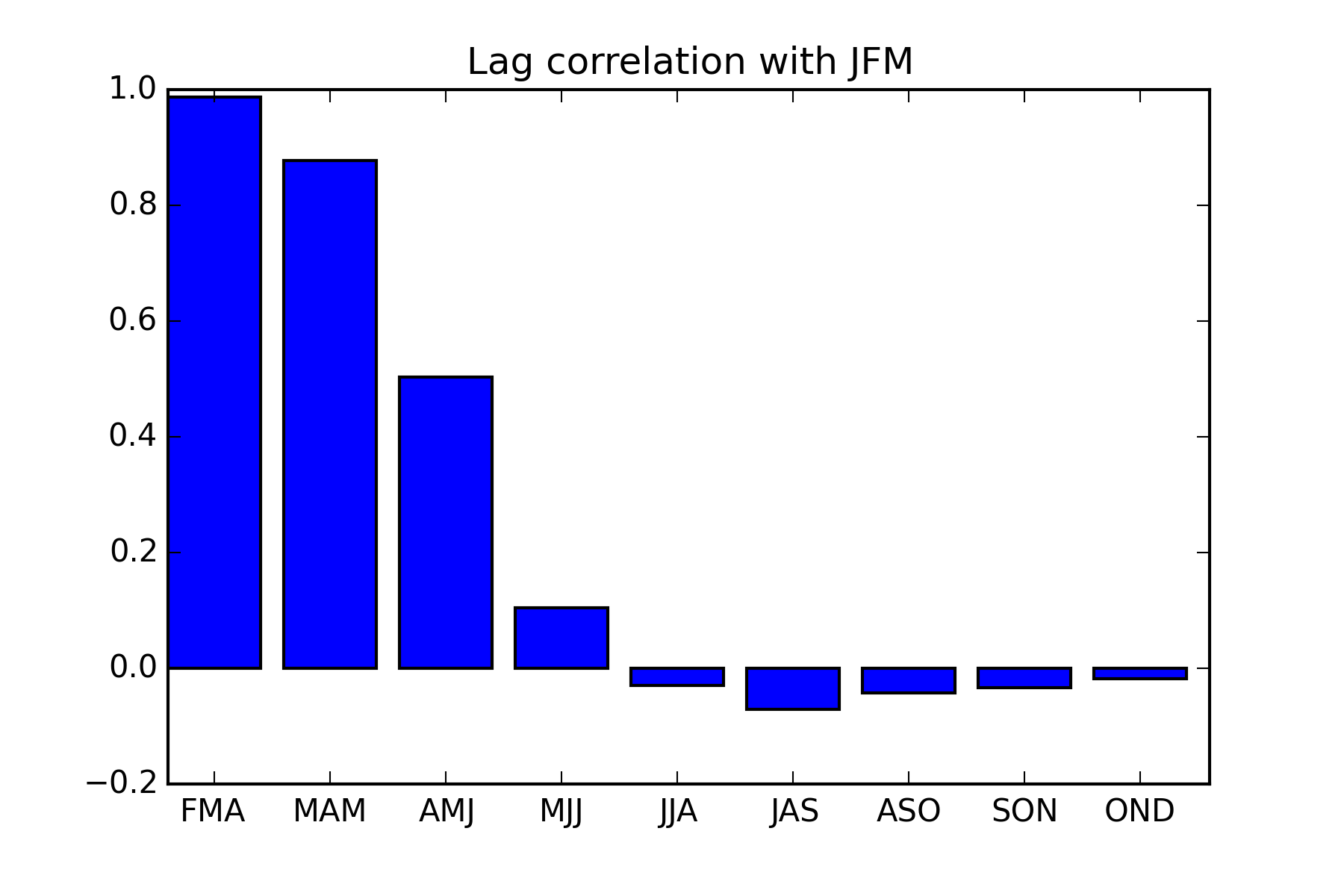

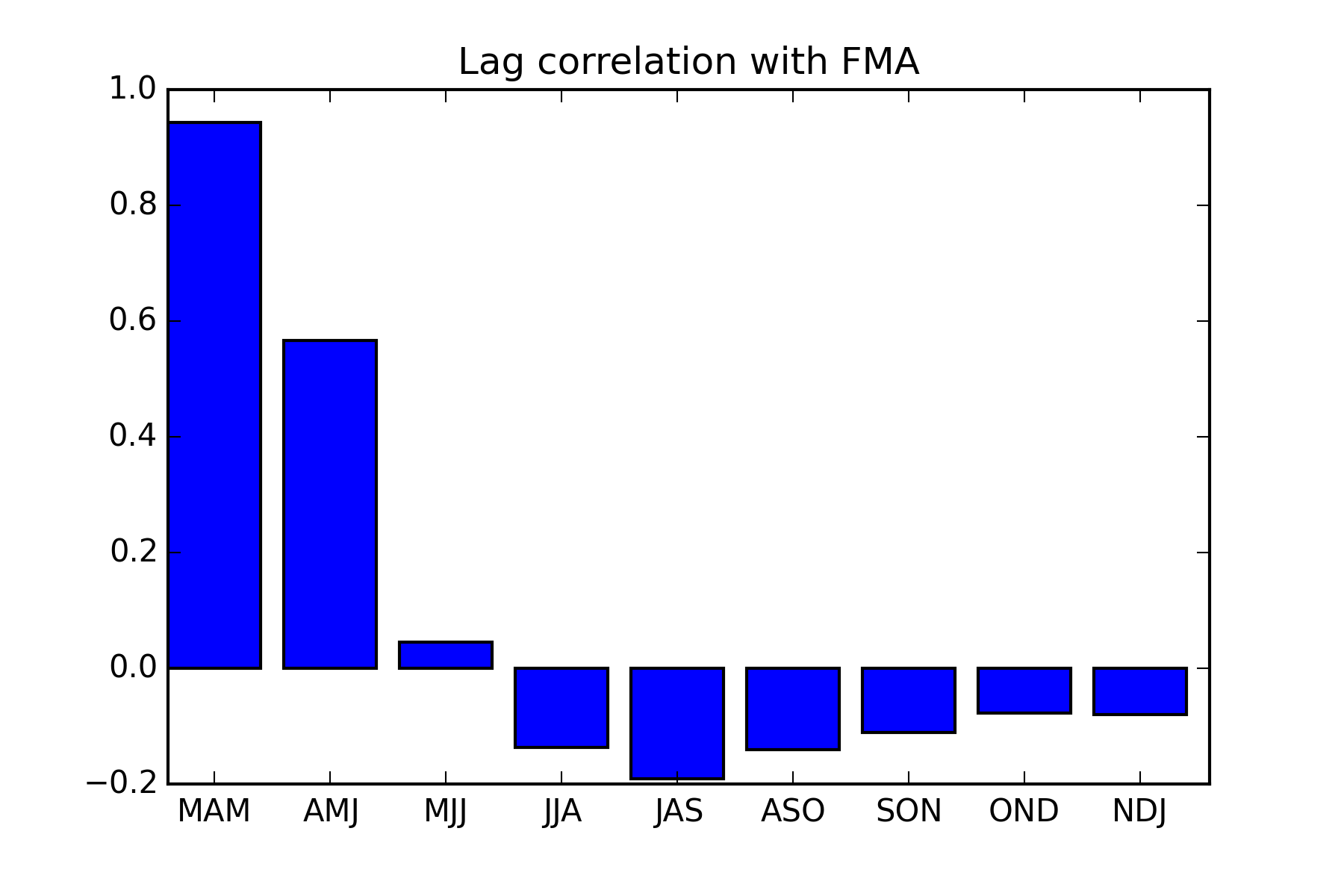

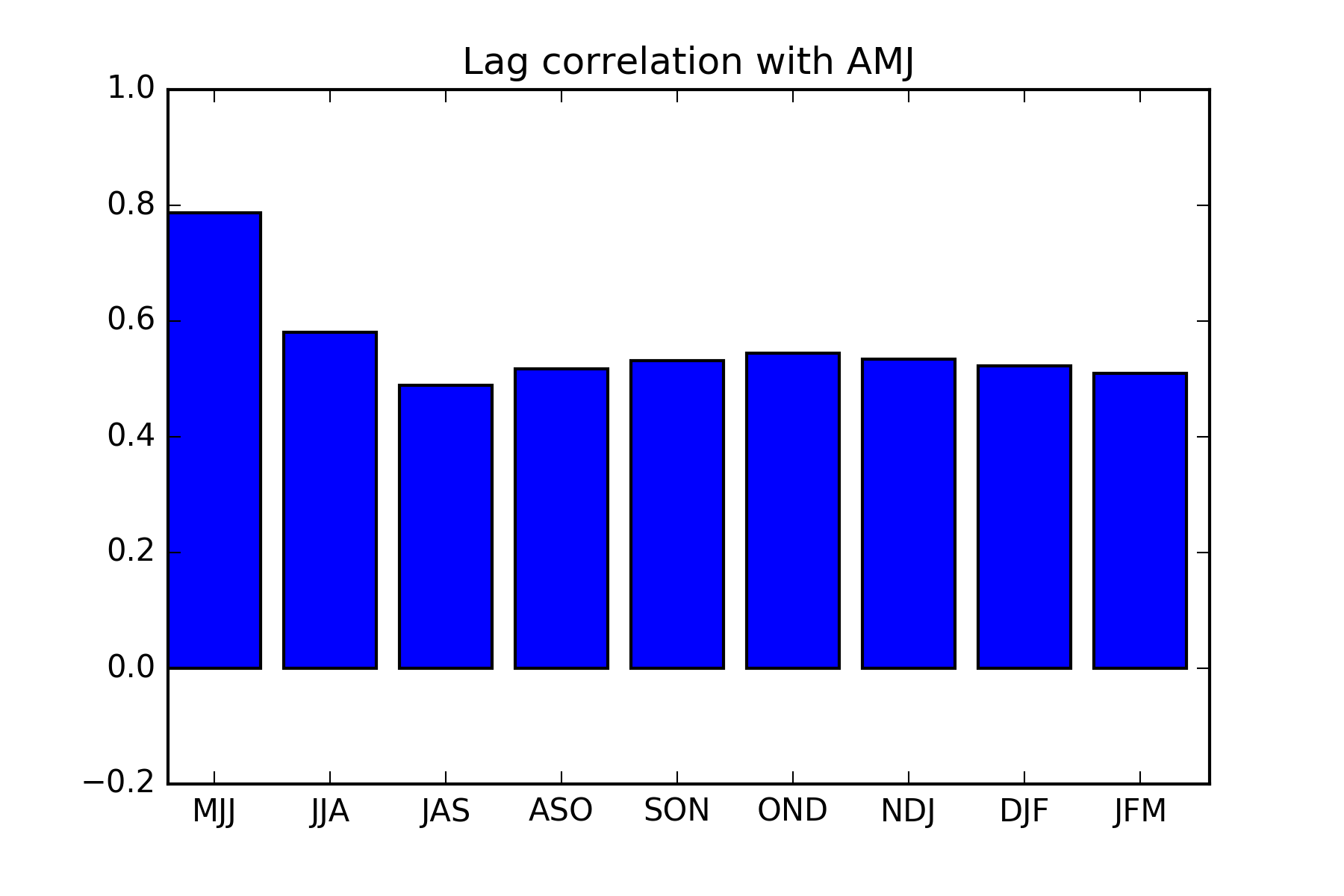

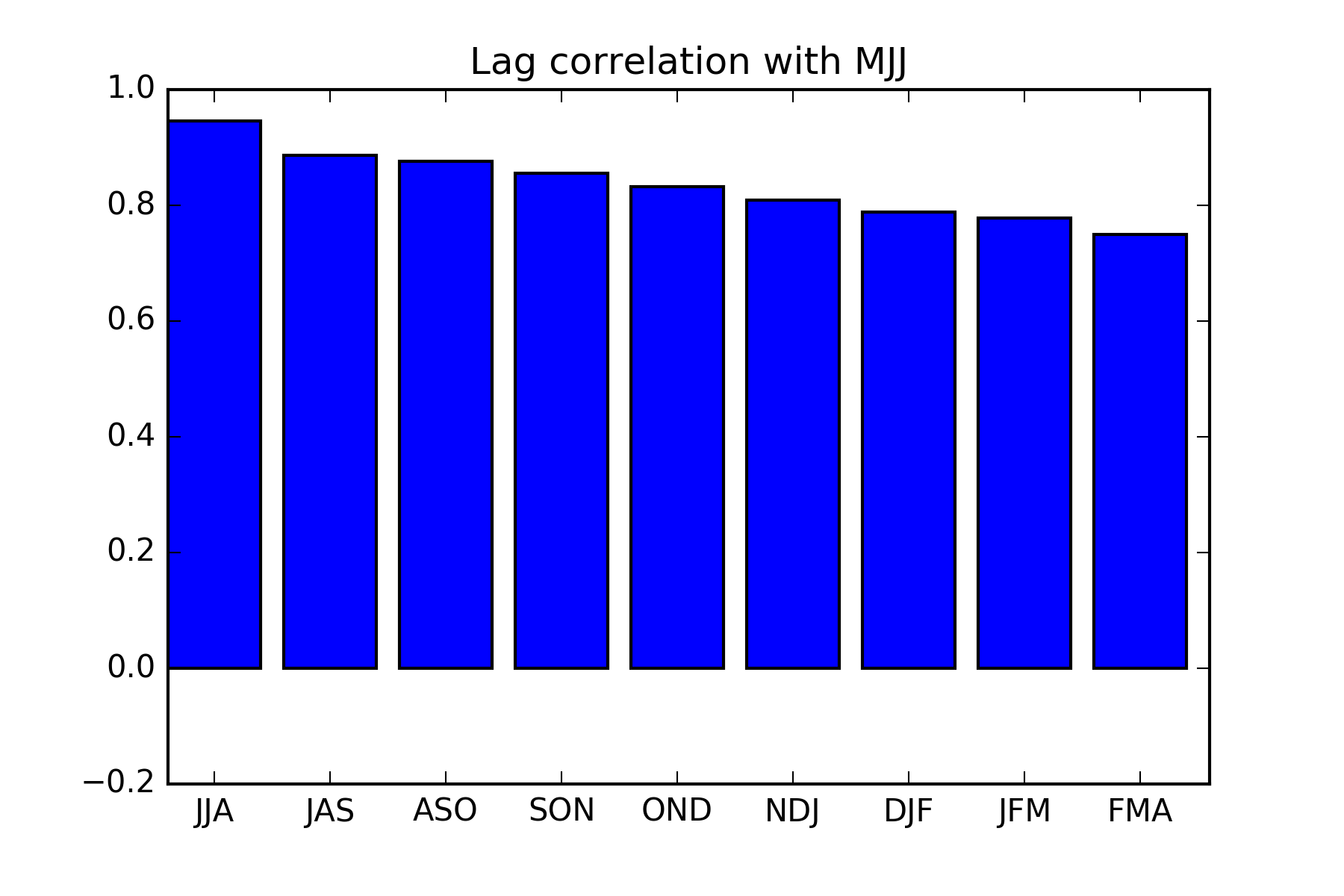

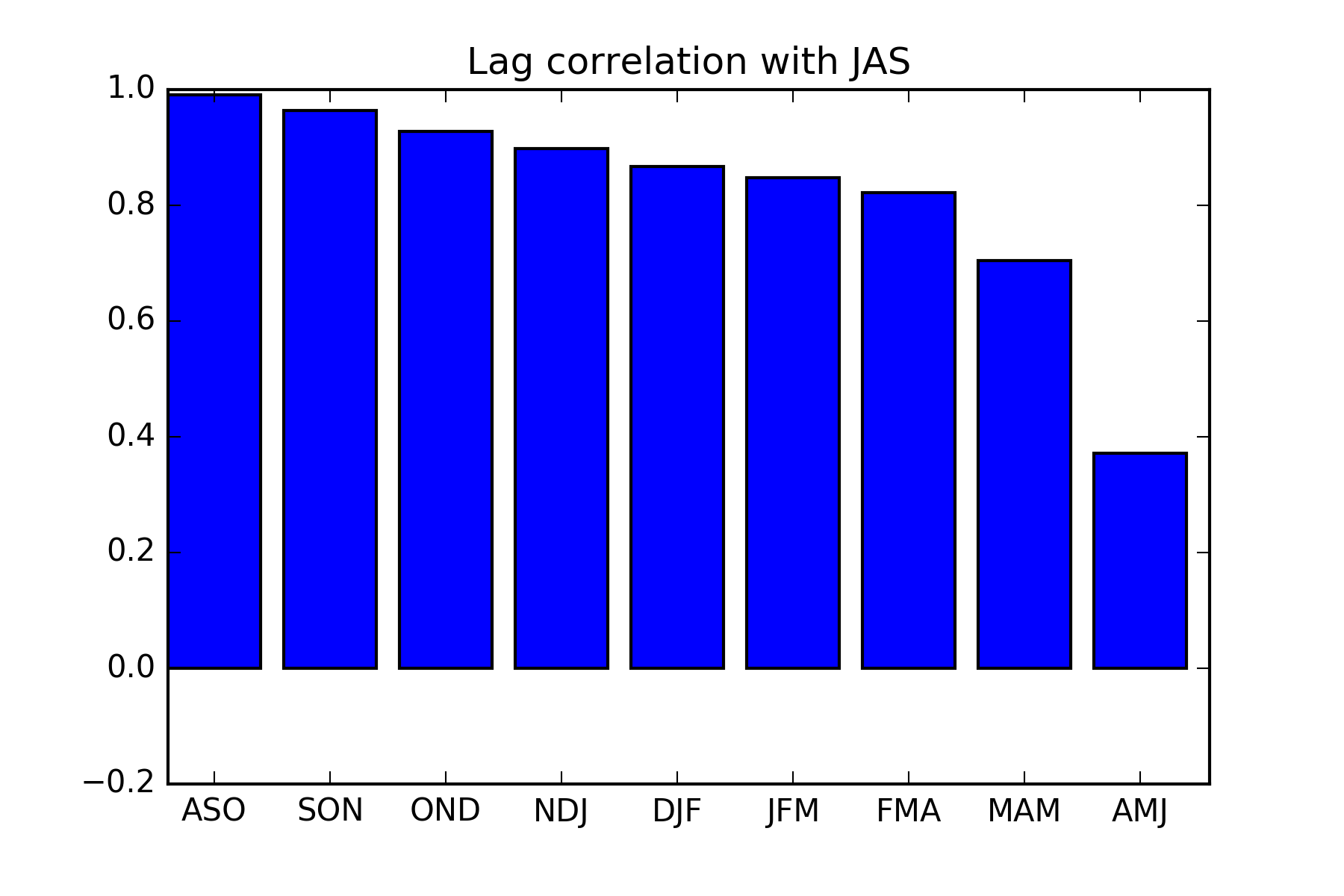

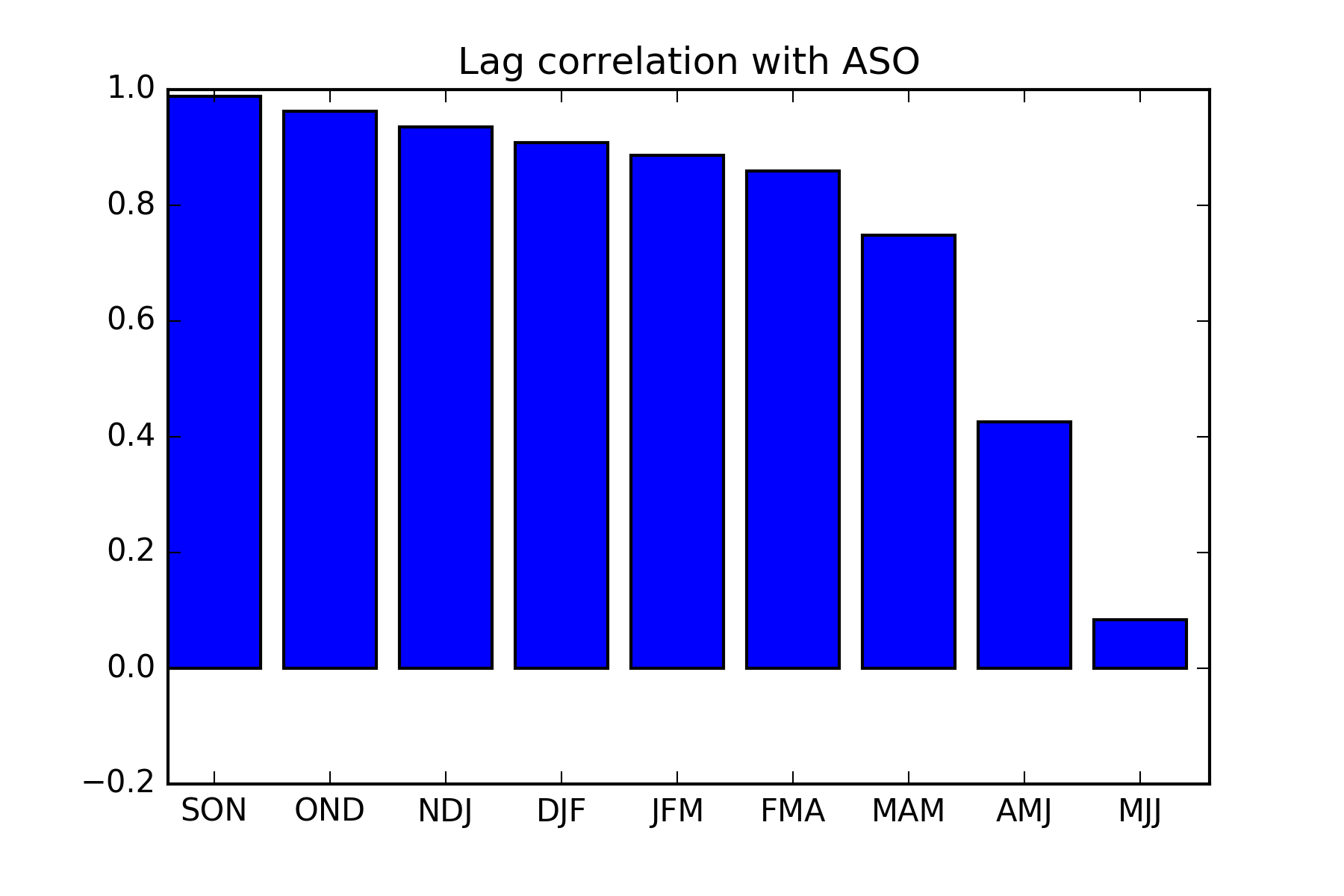

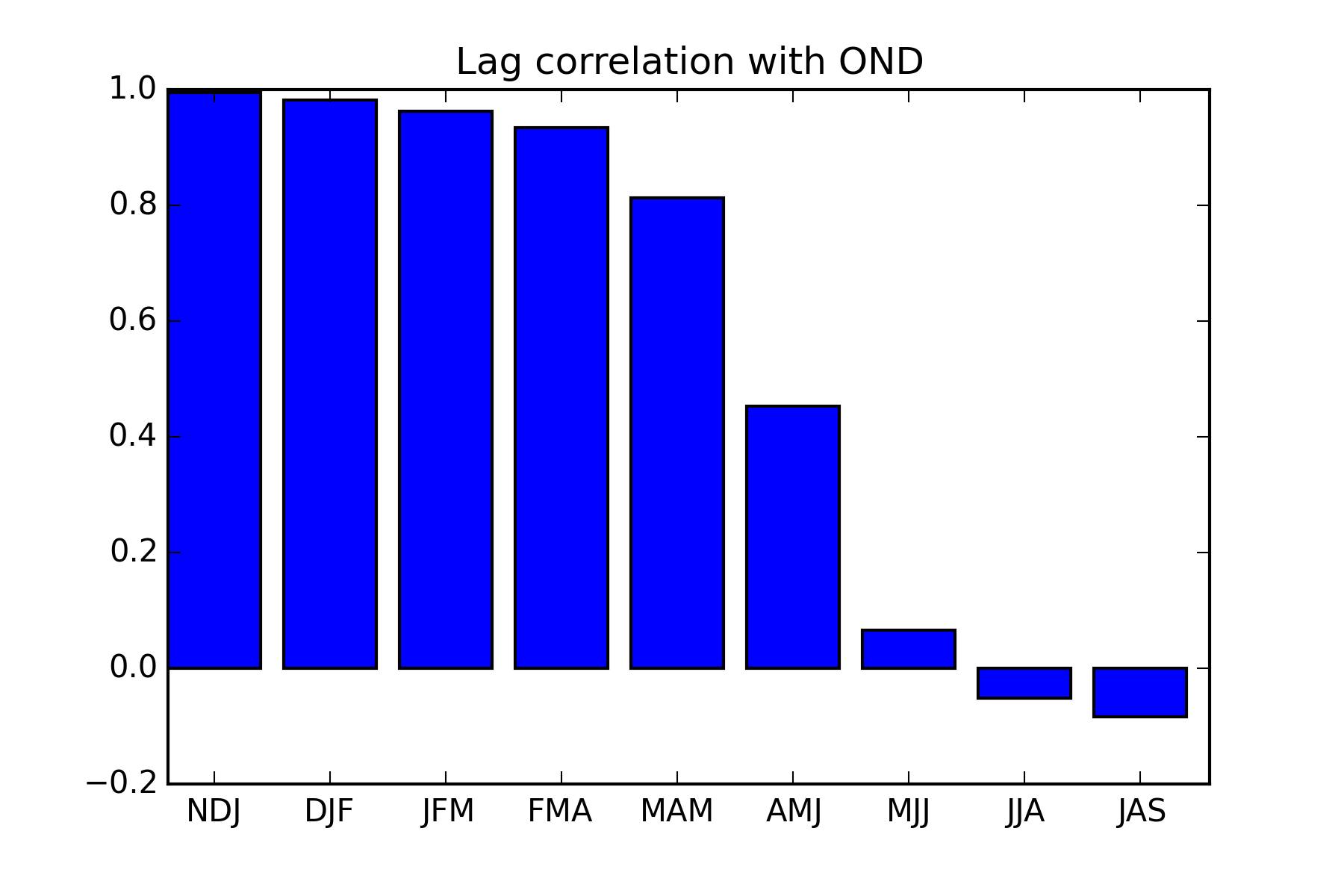

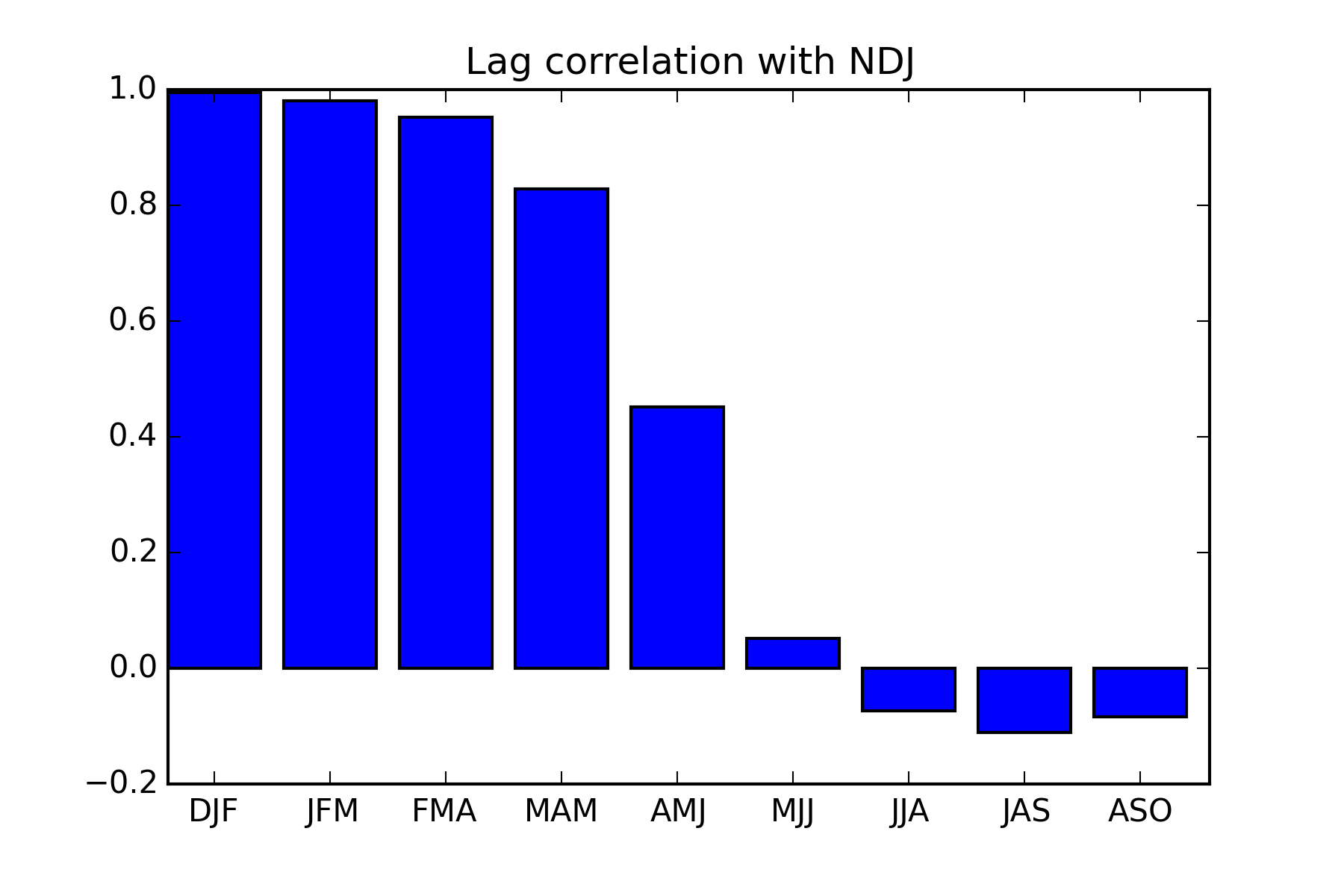
**

**Supplementary Figure 2** Same as Fig. 2, but for all other 8 3-month means. Figure is generated by Python (https://www.python.org/)

**
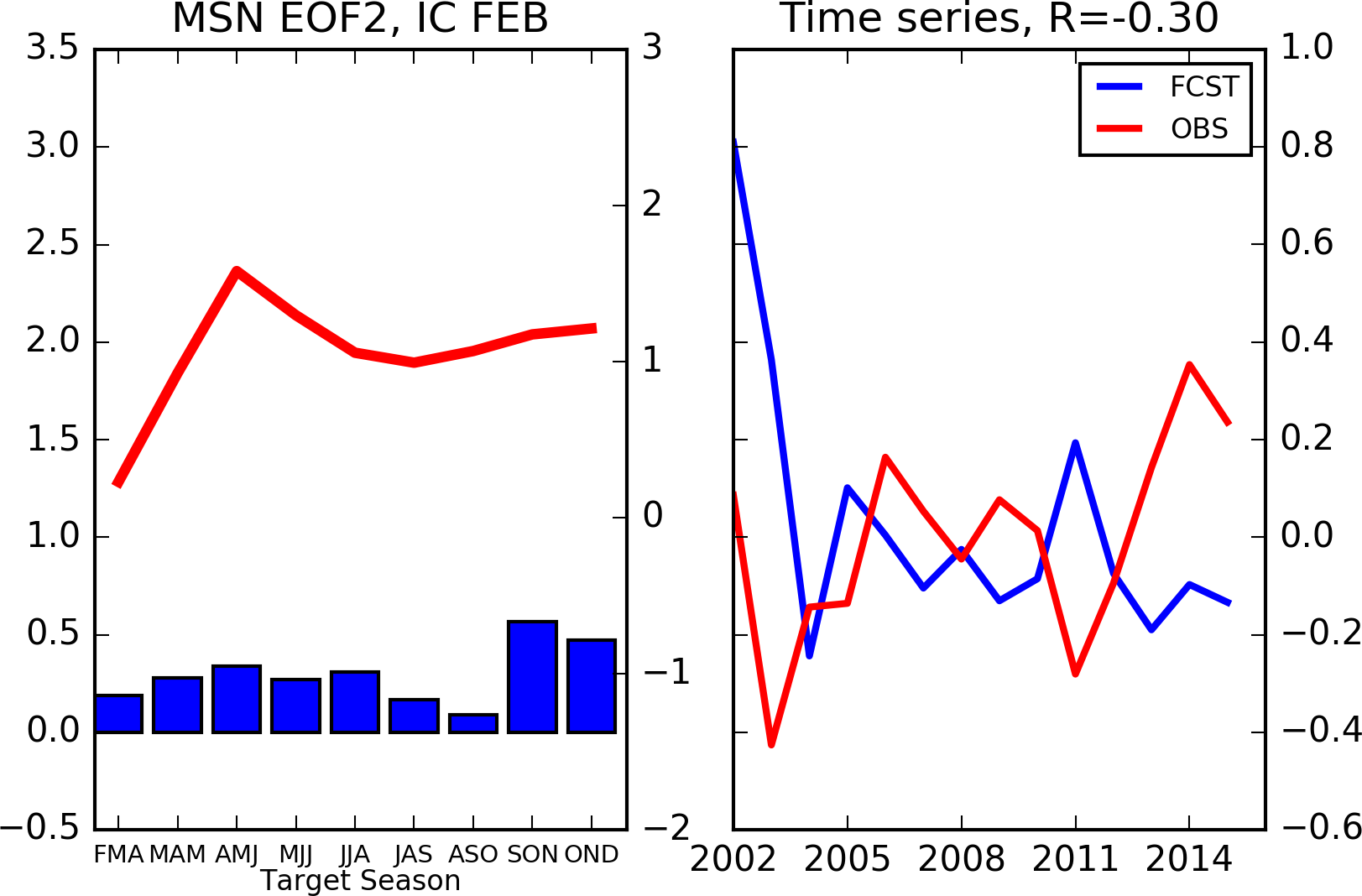

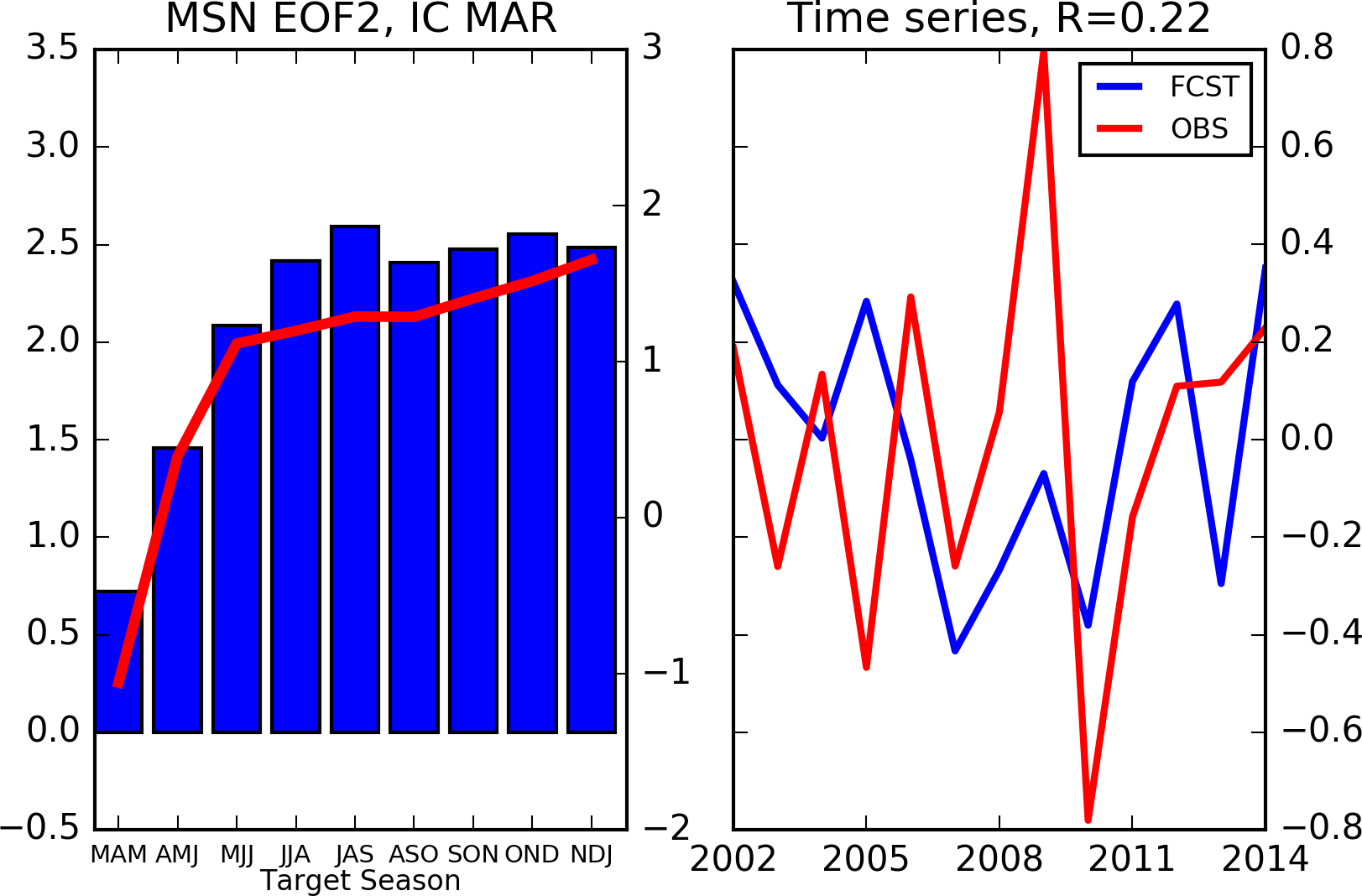

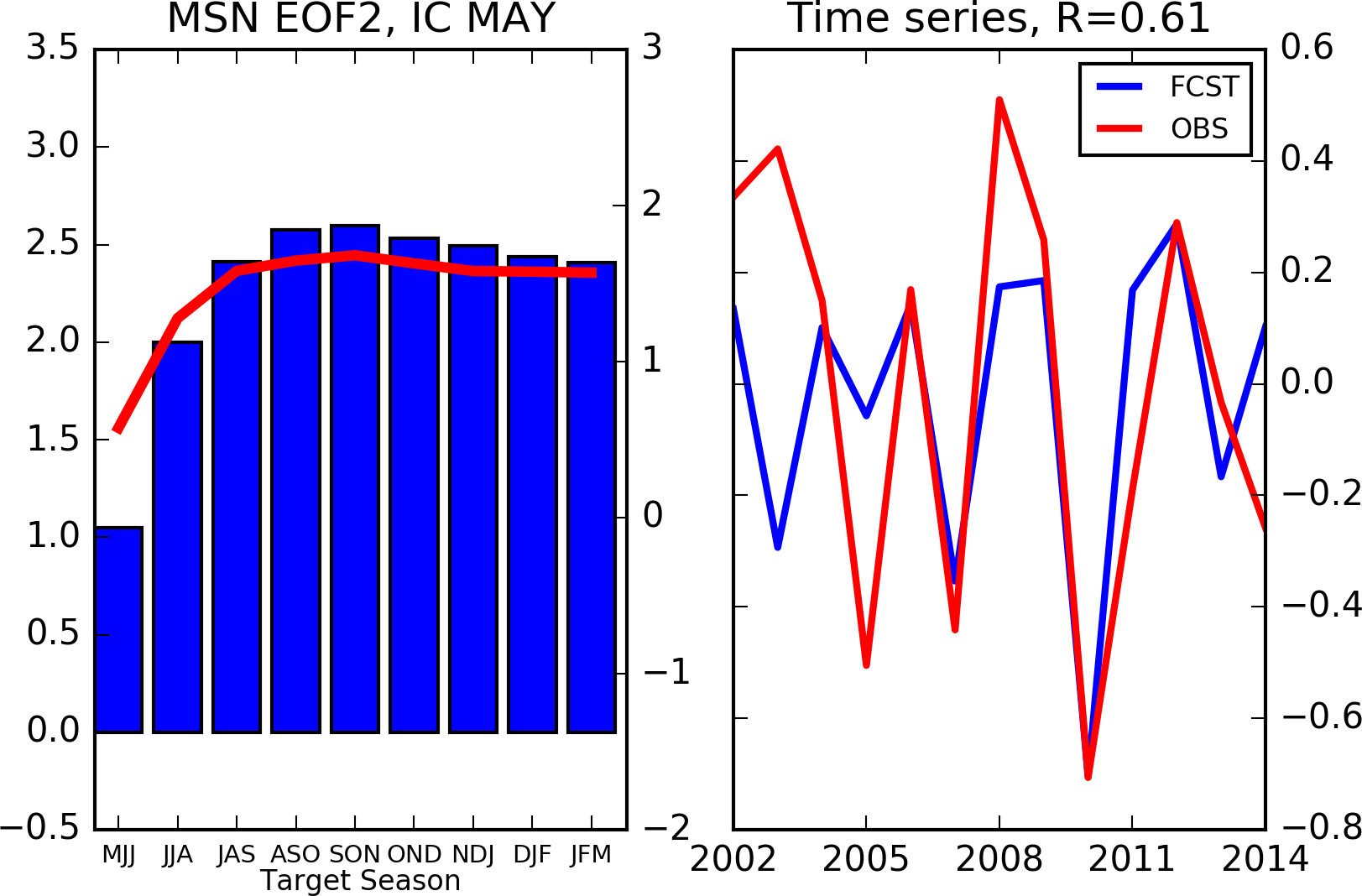

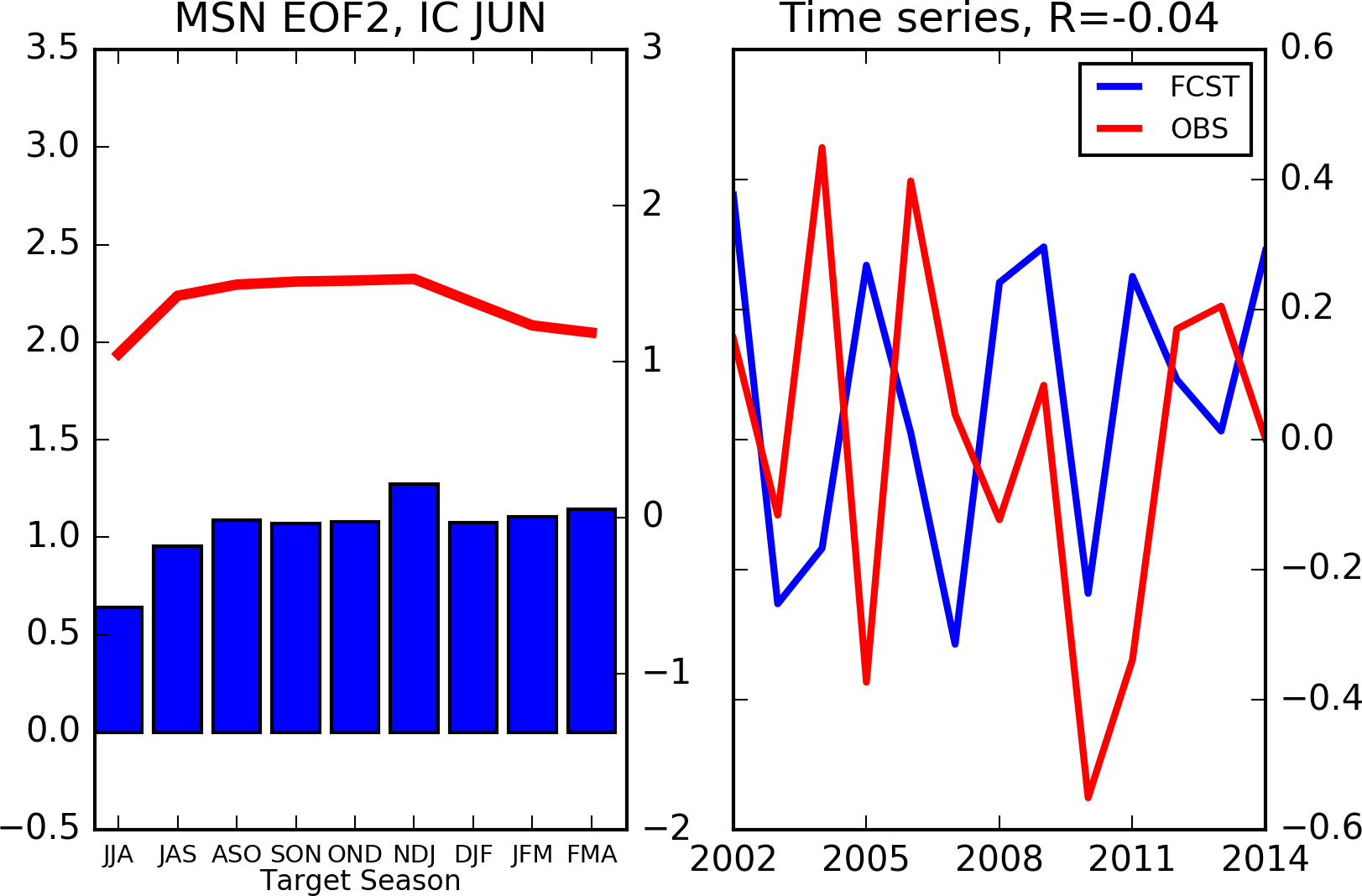

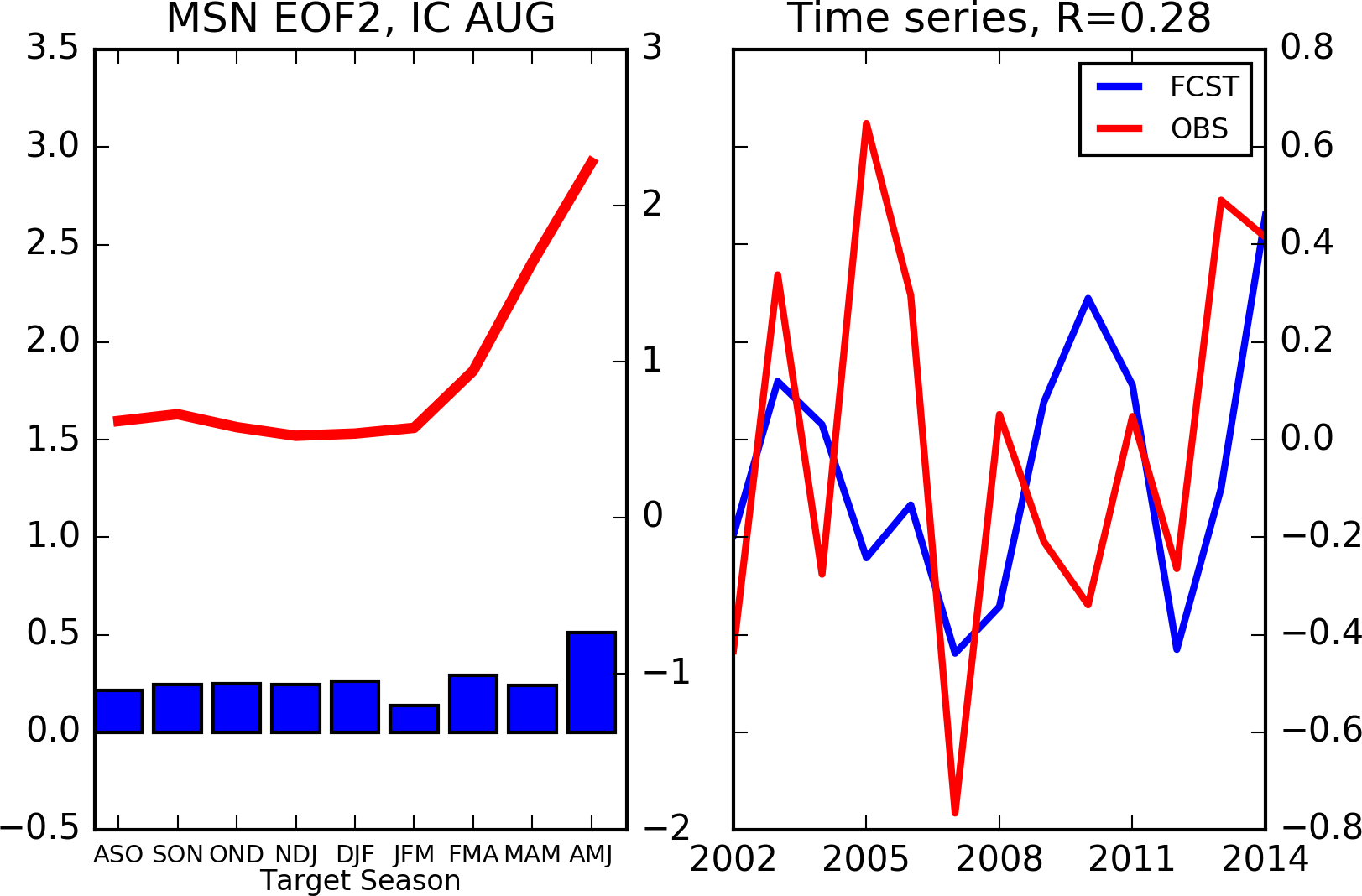

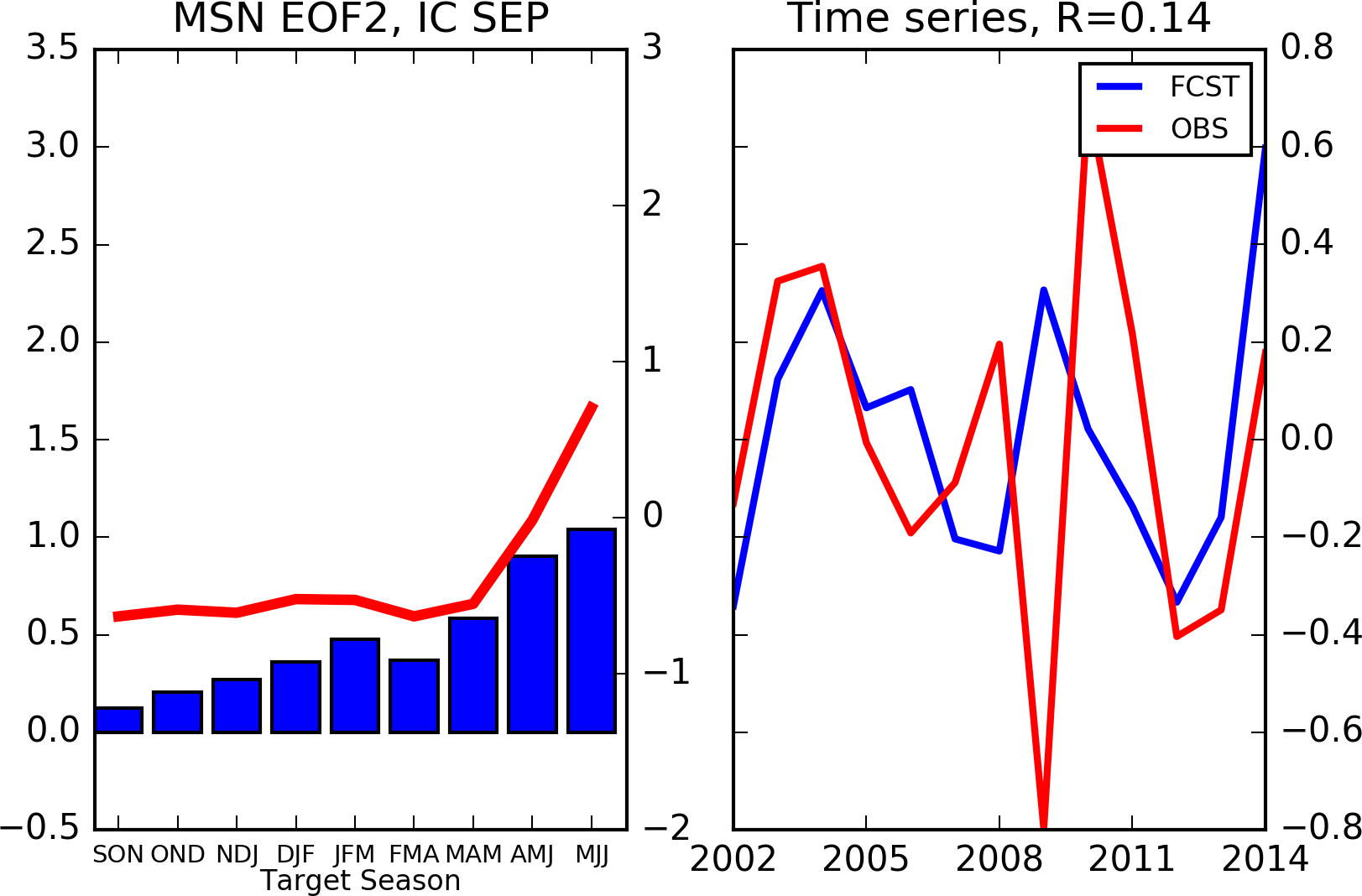

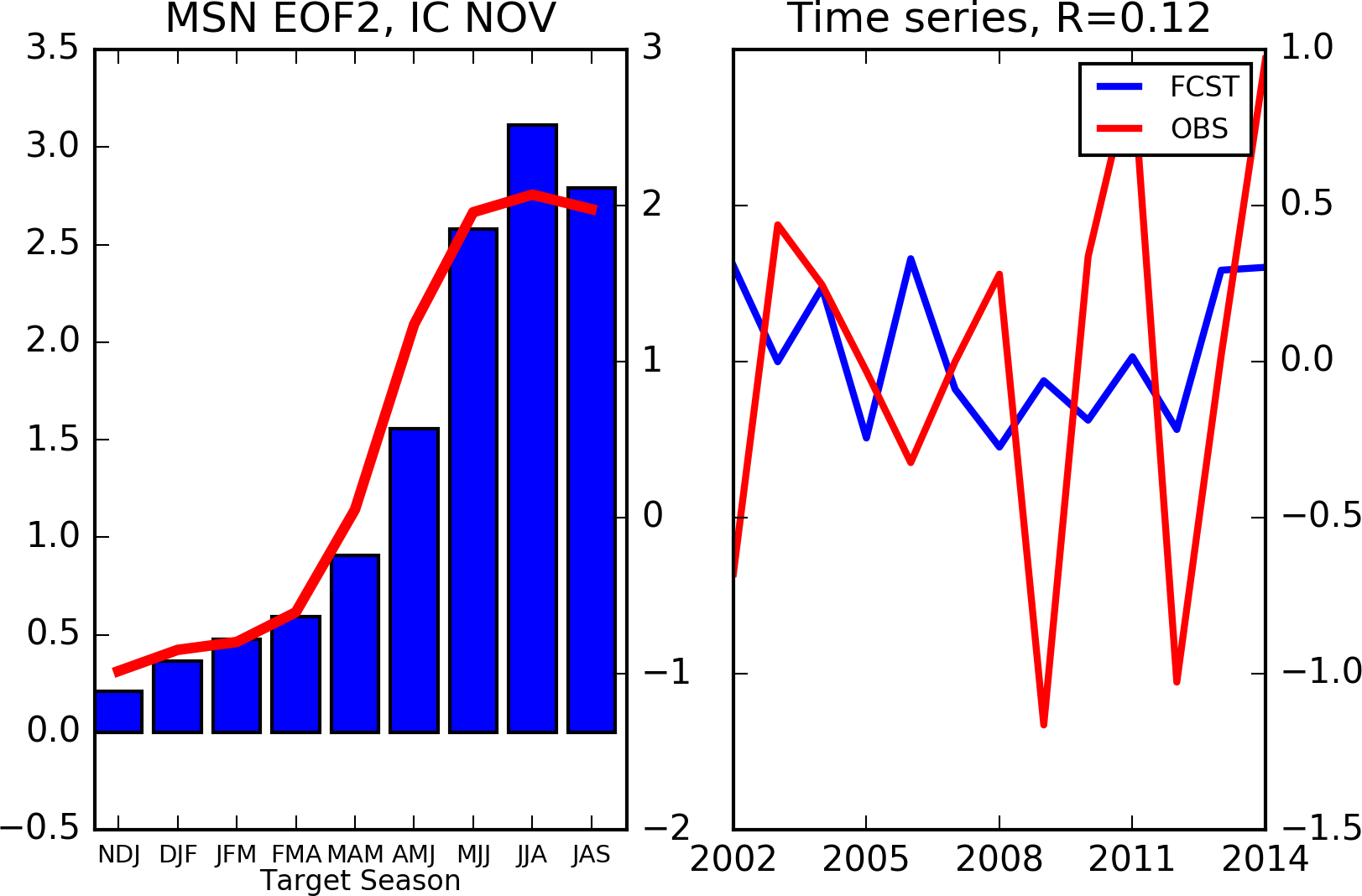

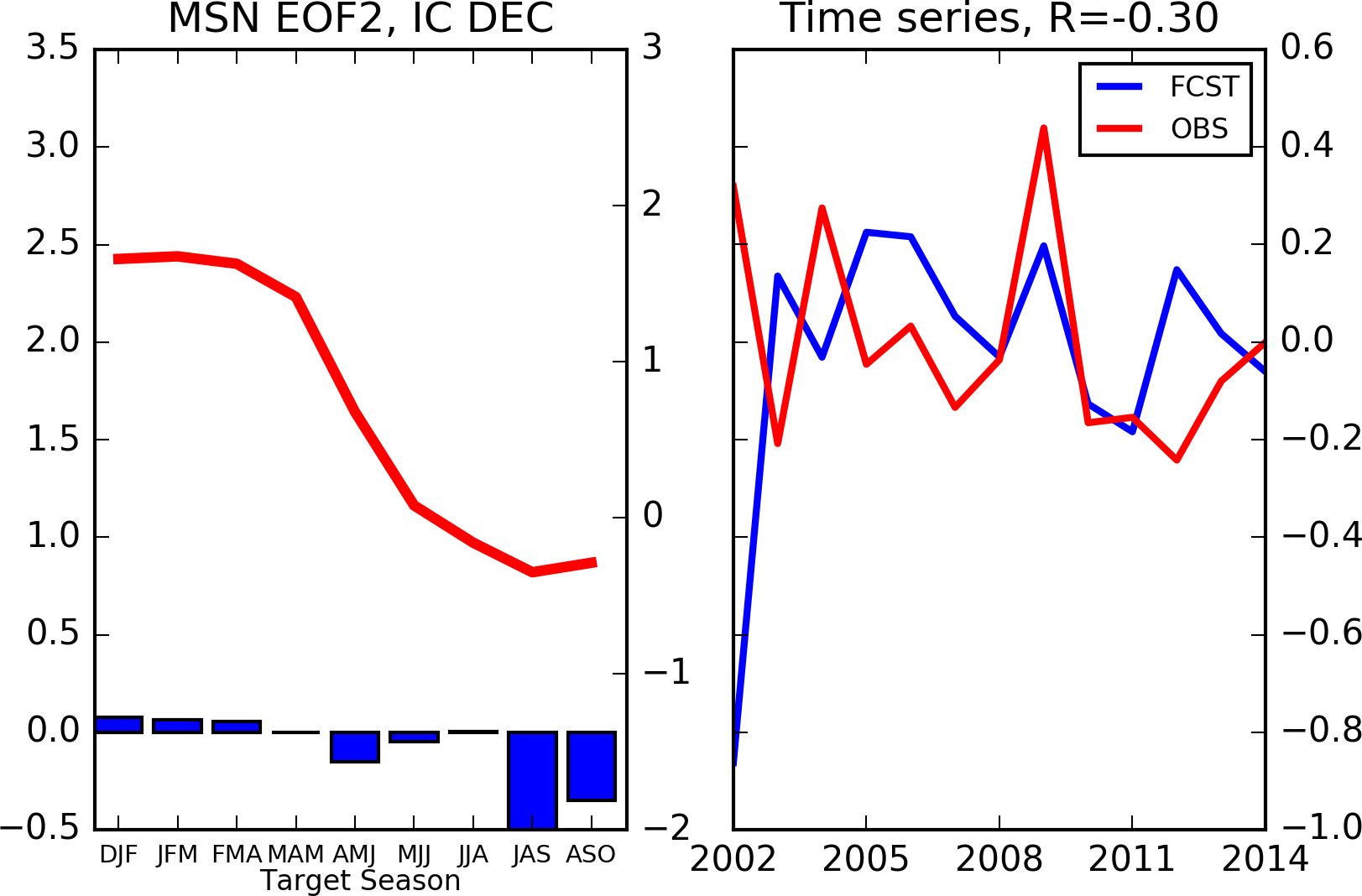
**

**Supplementary Figure 3** Same as Fig. 3, but for ICs in all other 8 months. Figure is generated by Python (https://www.python.org/)


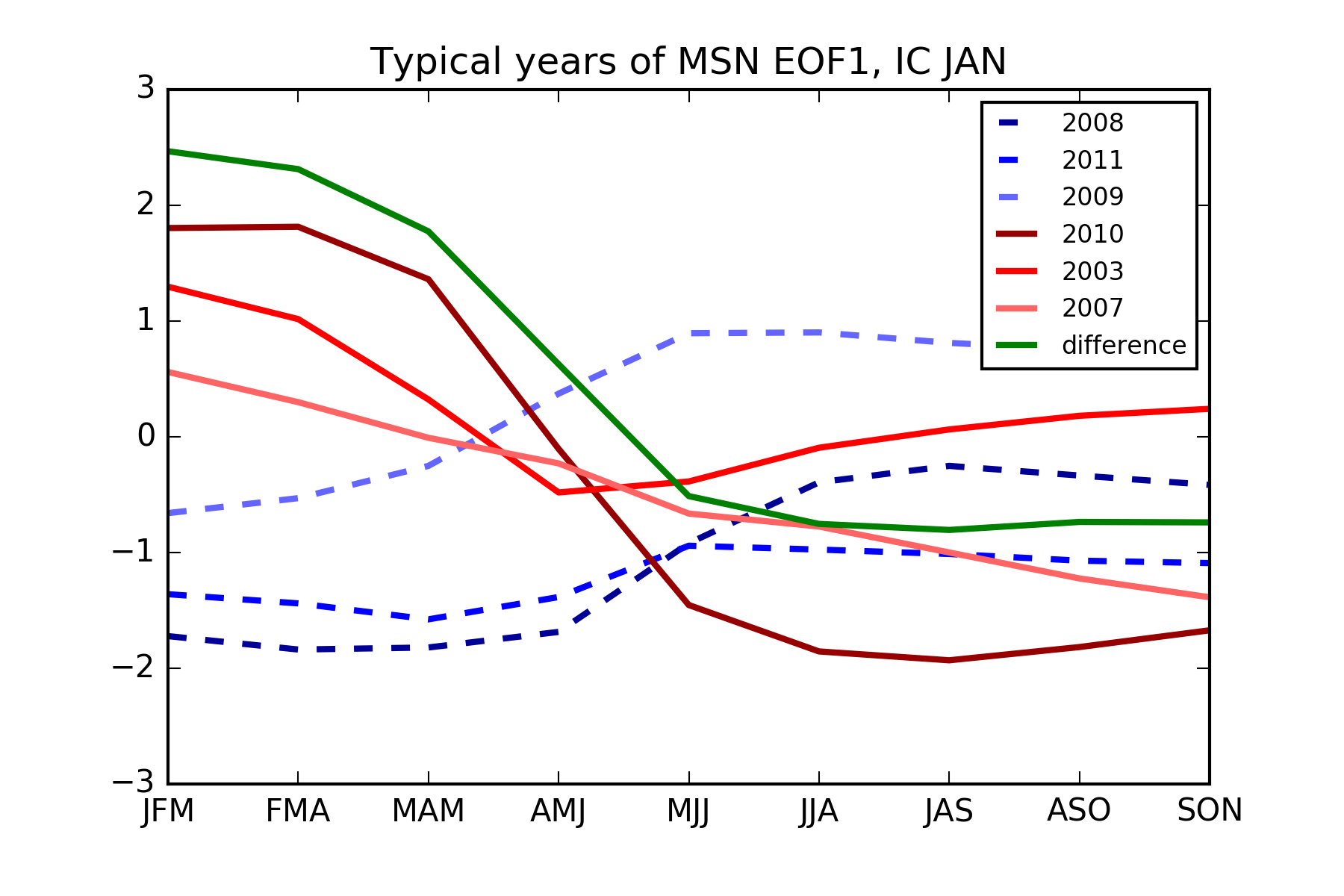

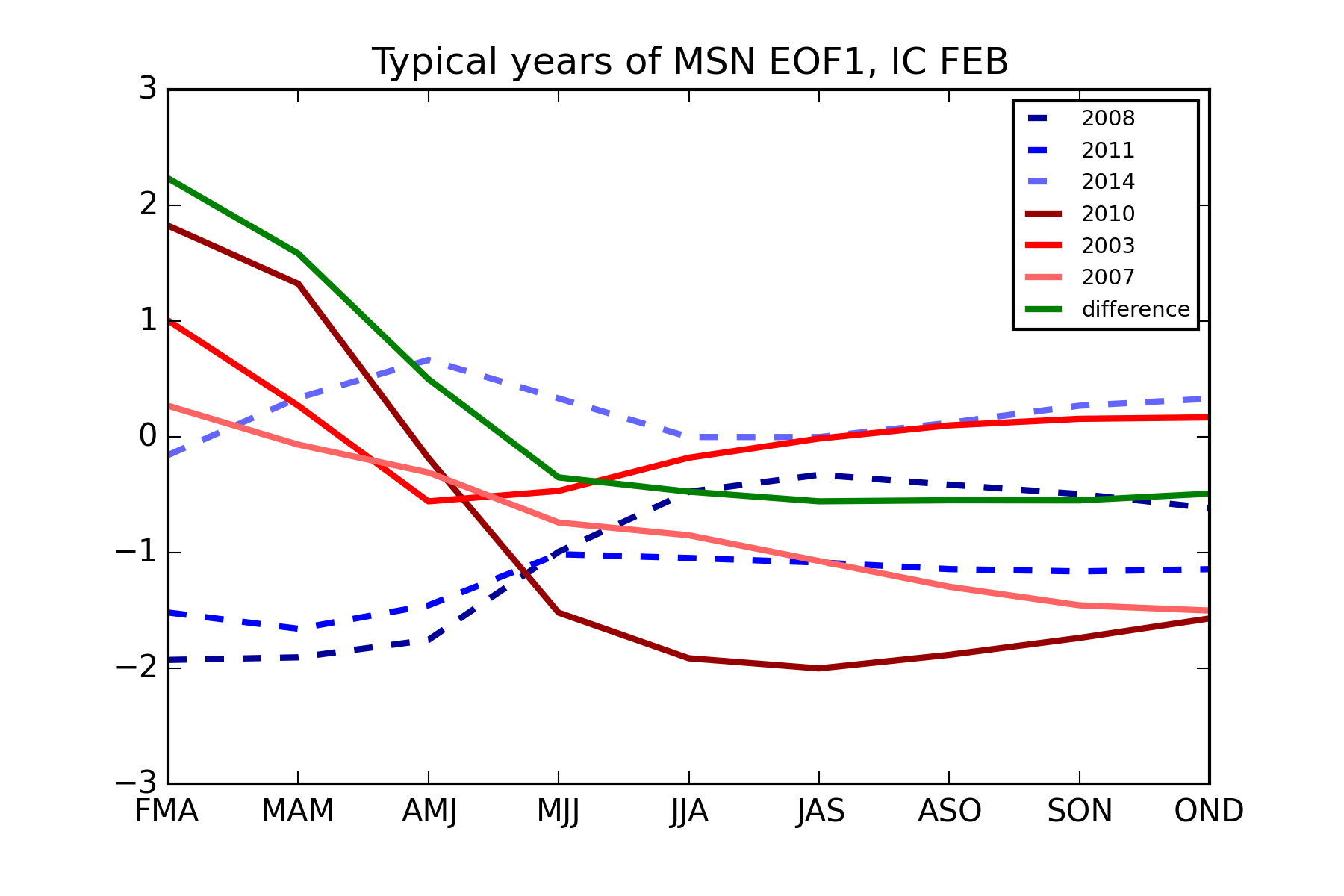


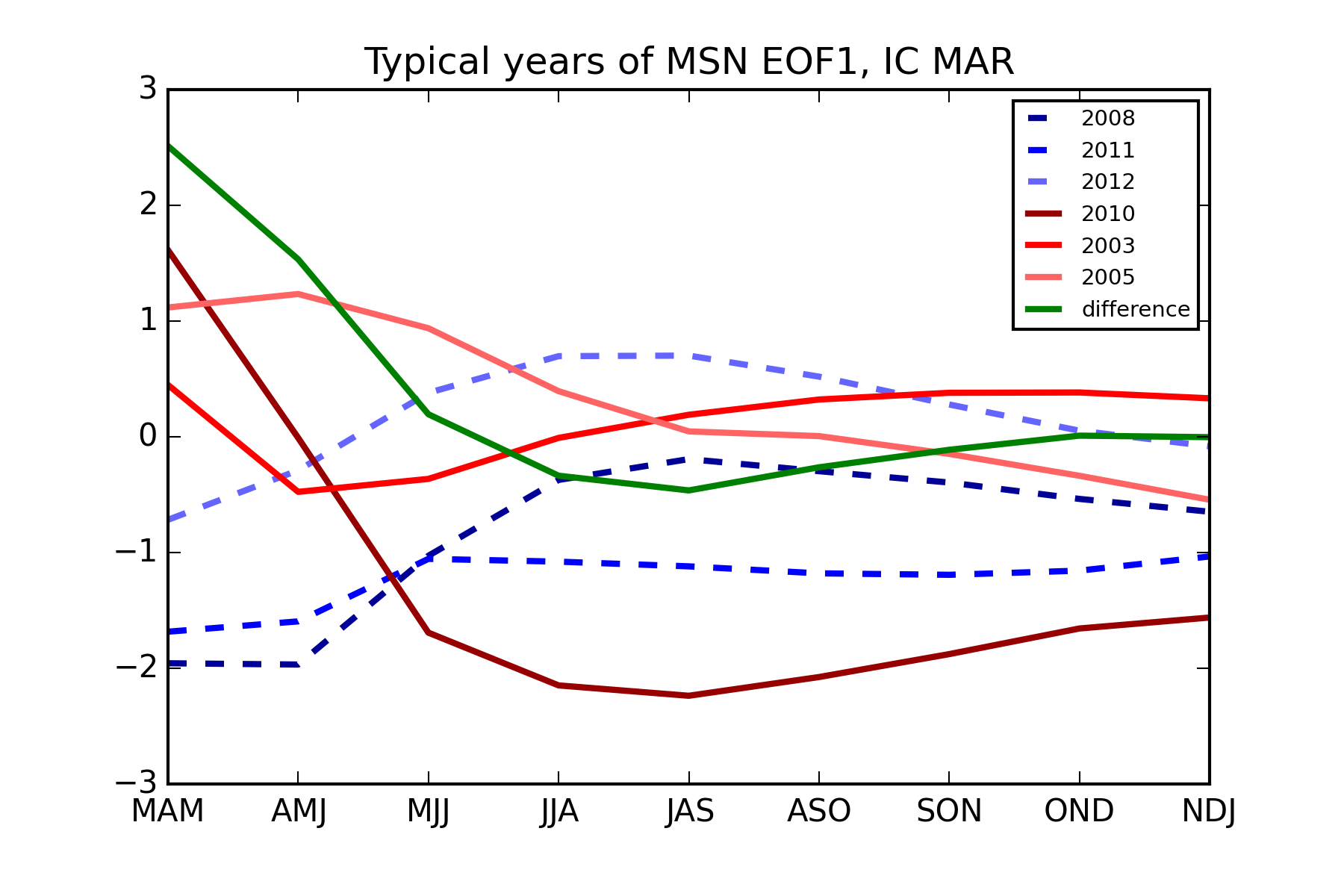

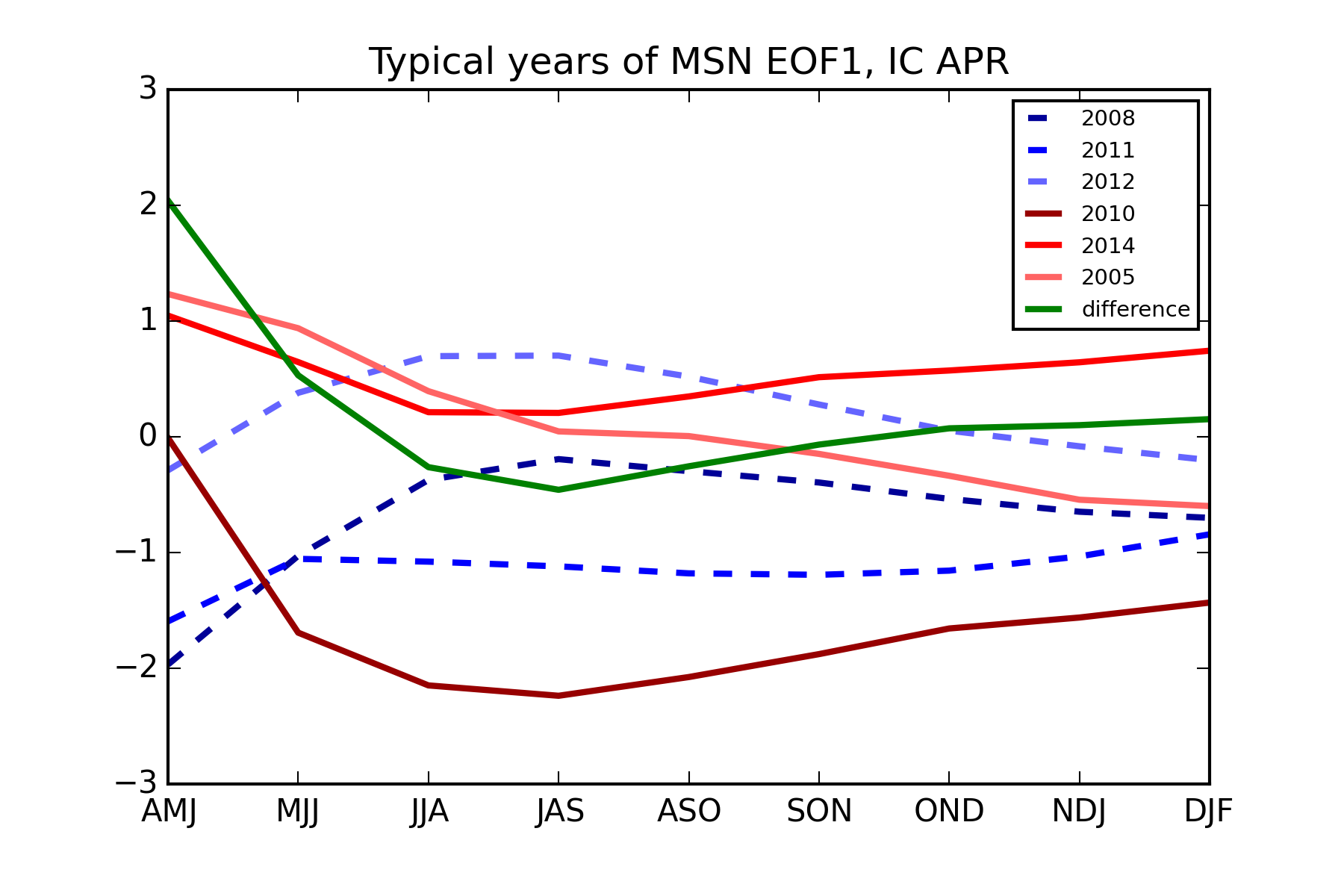

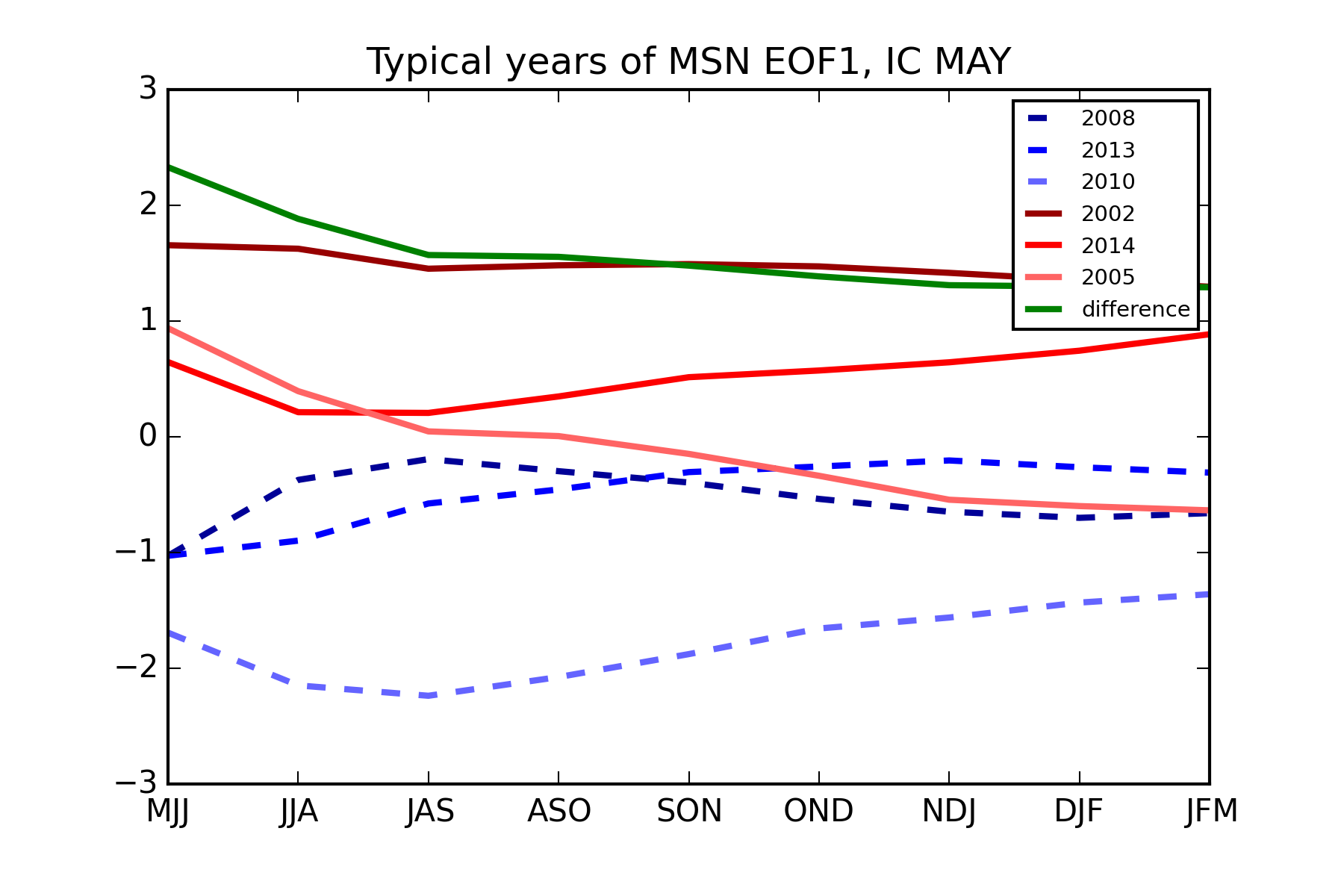

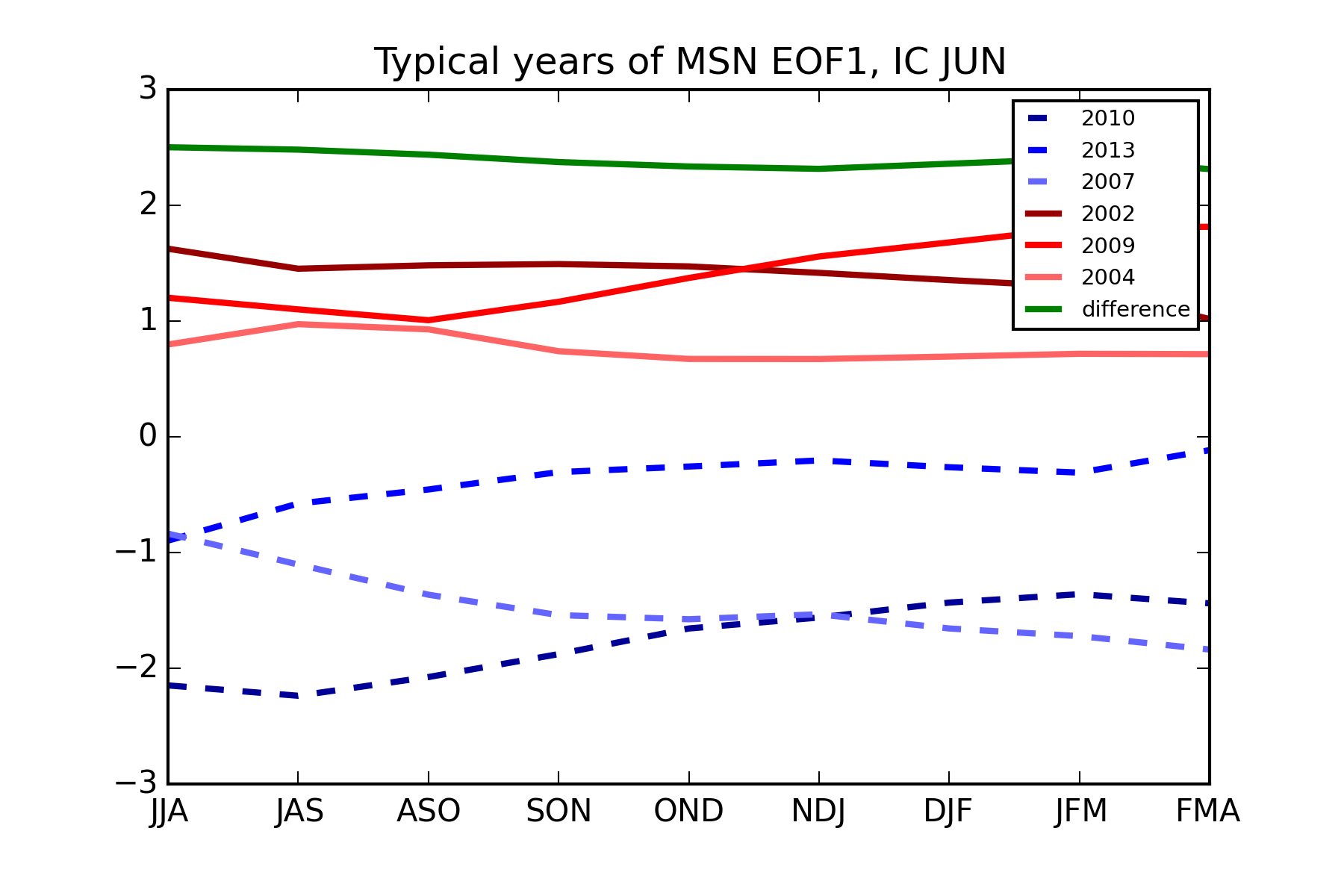

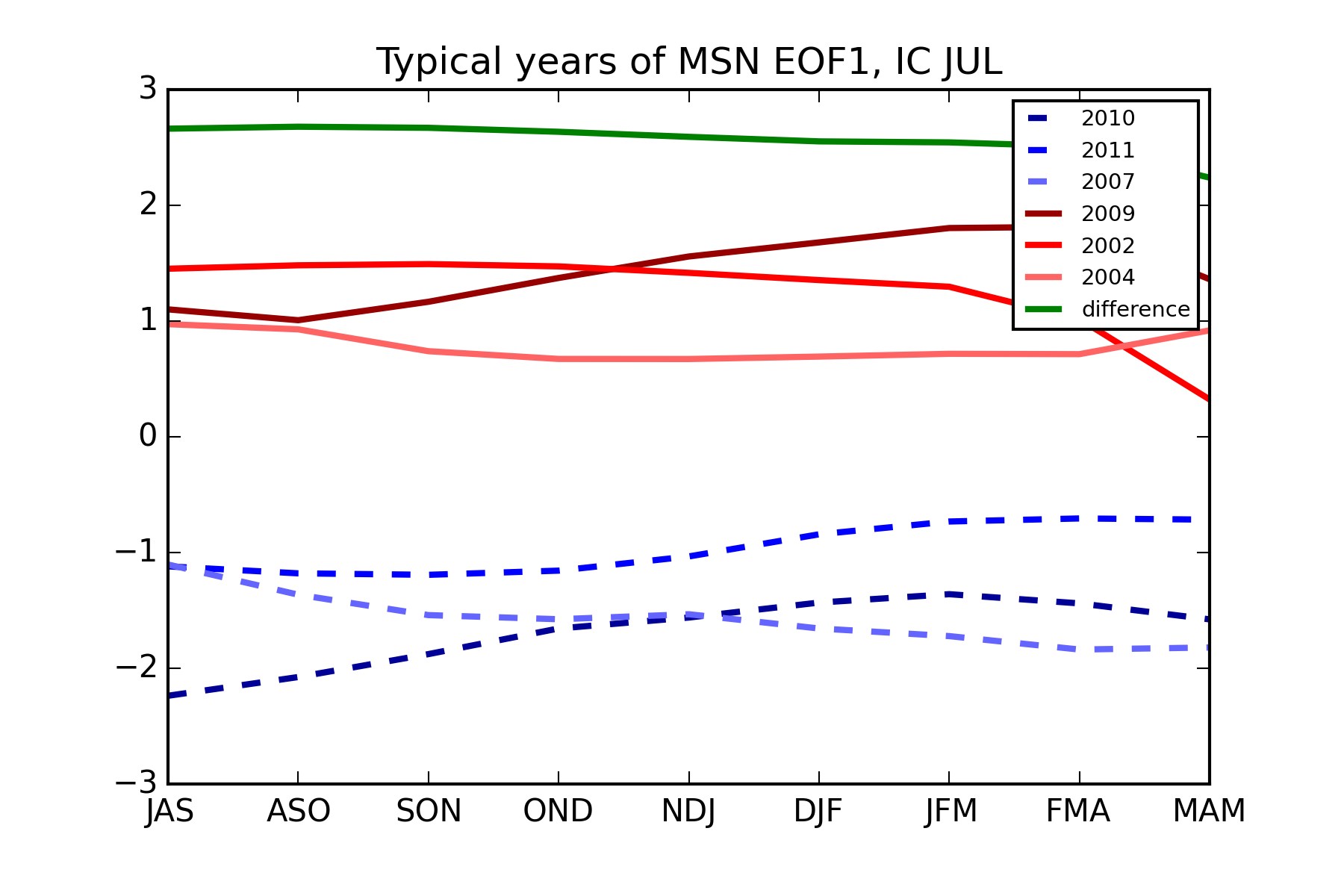

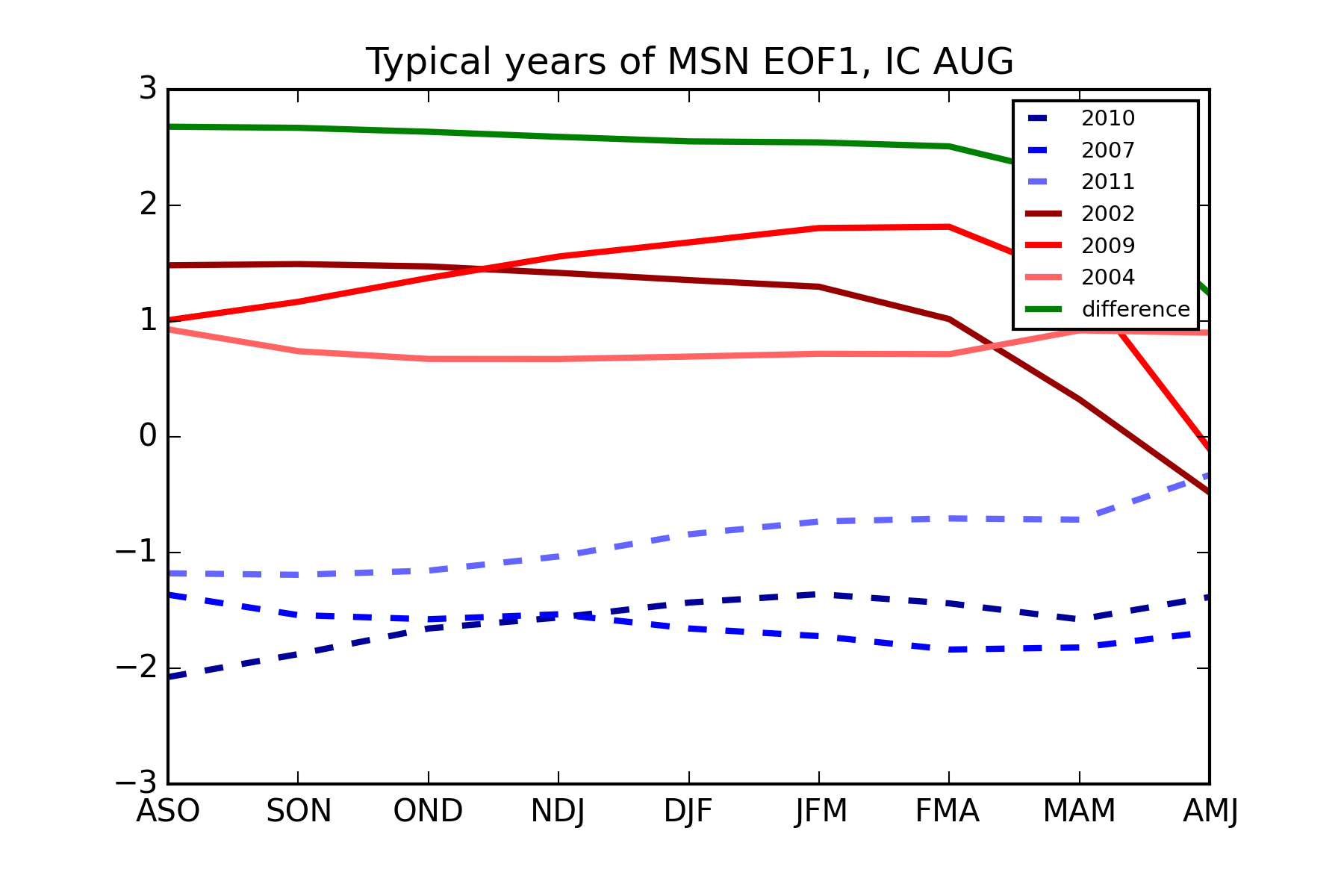

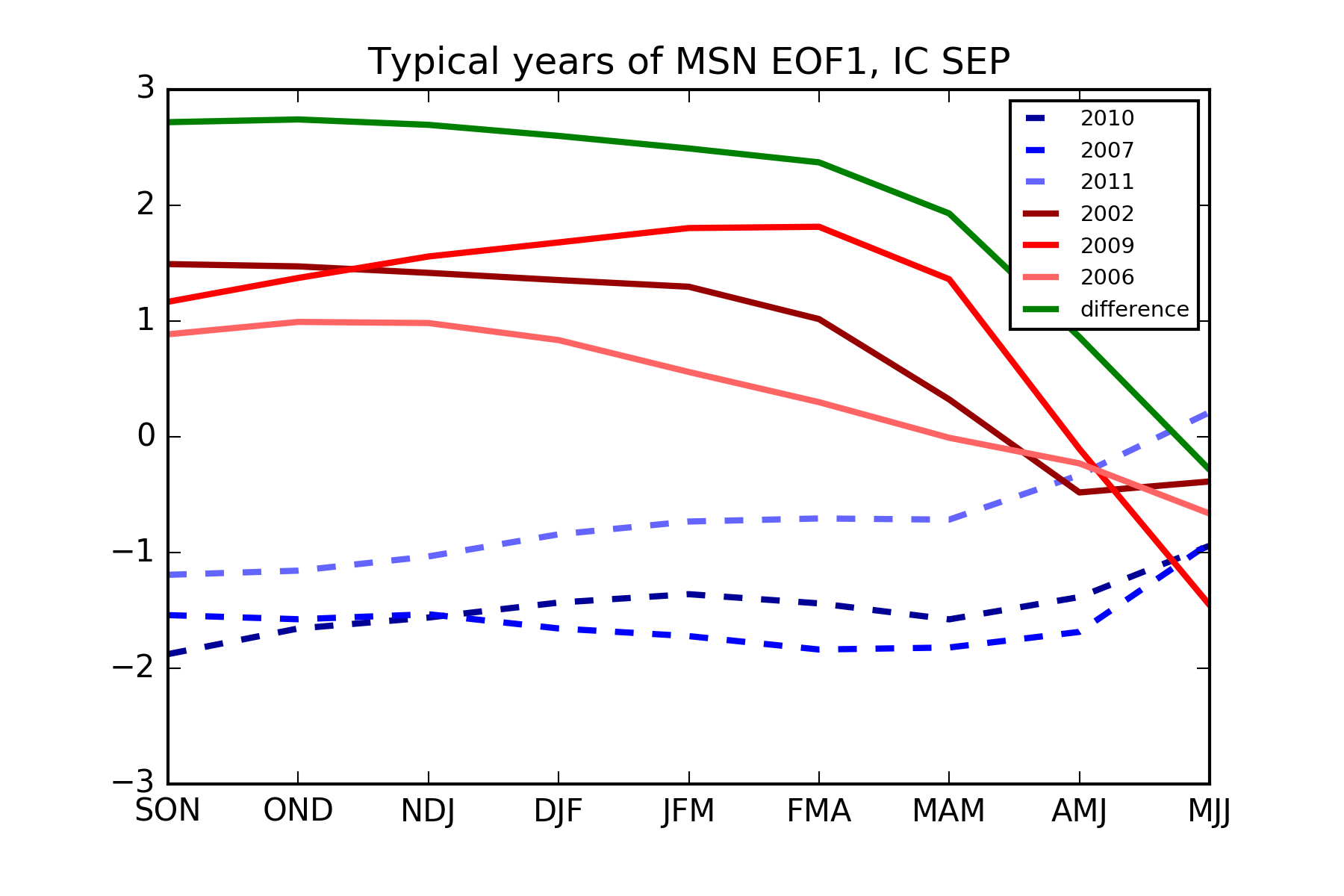

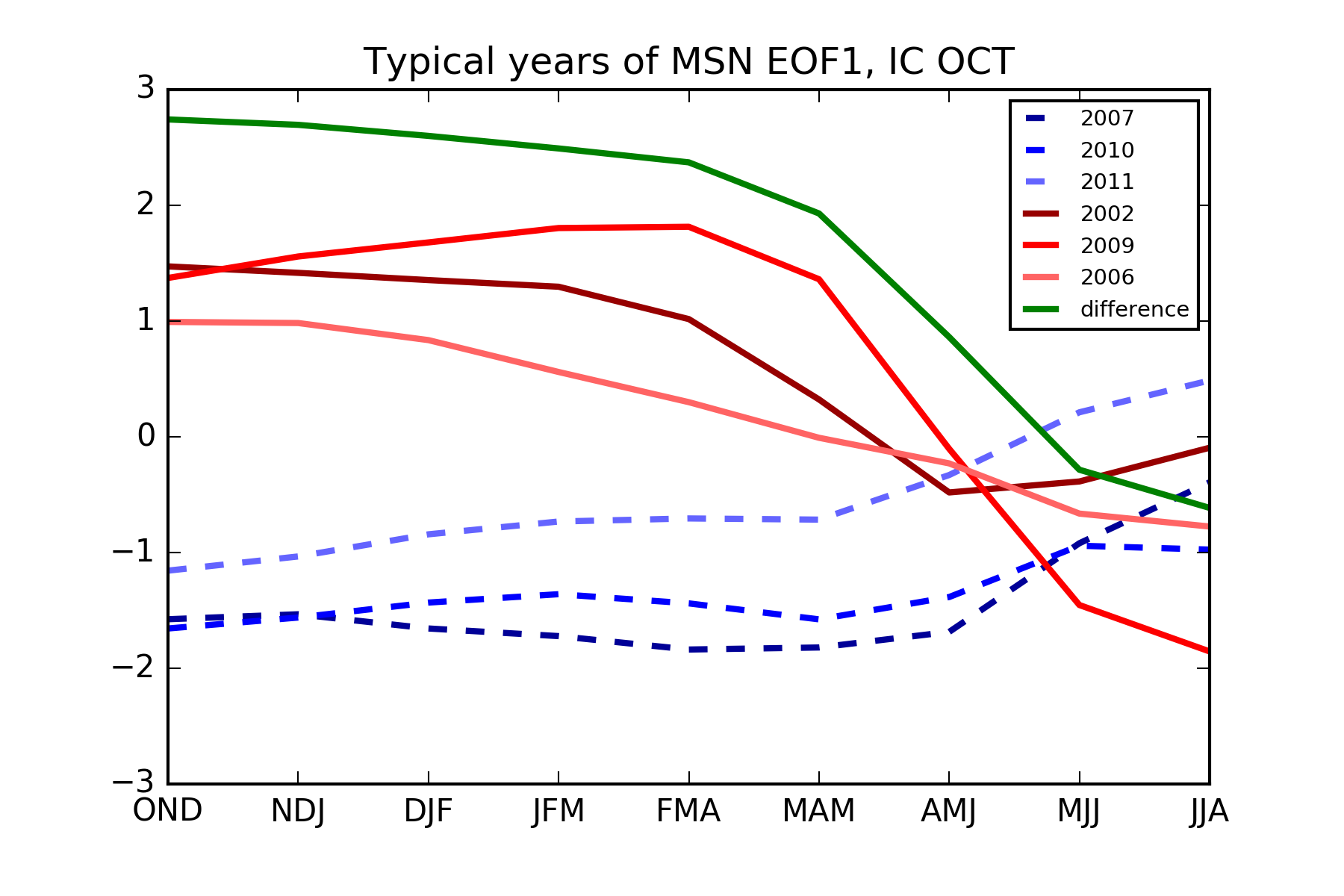

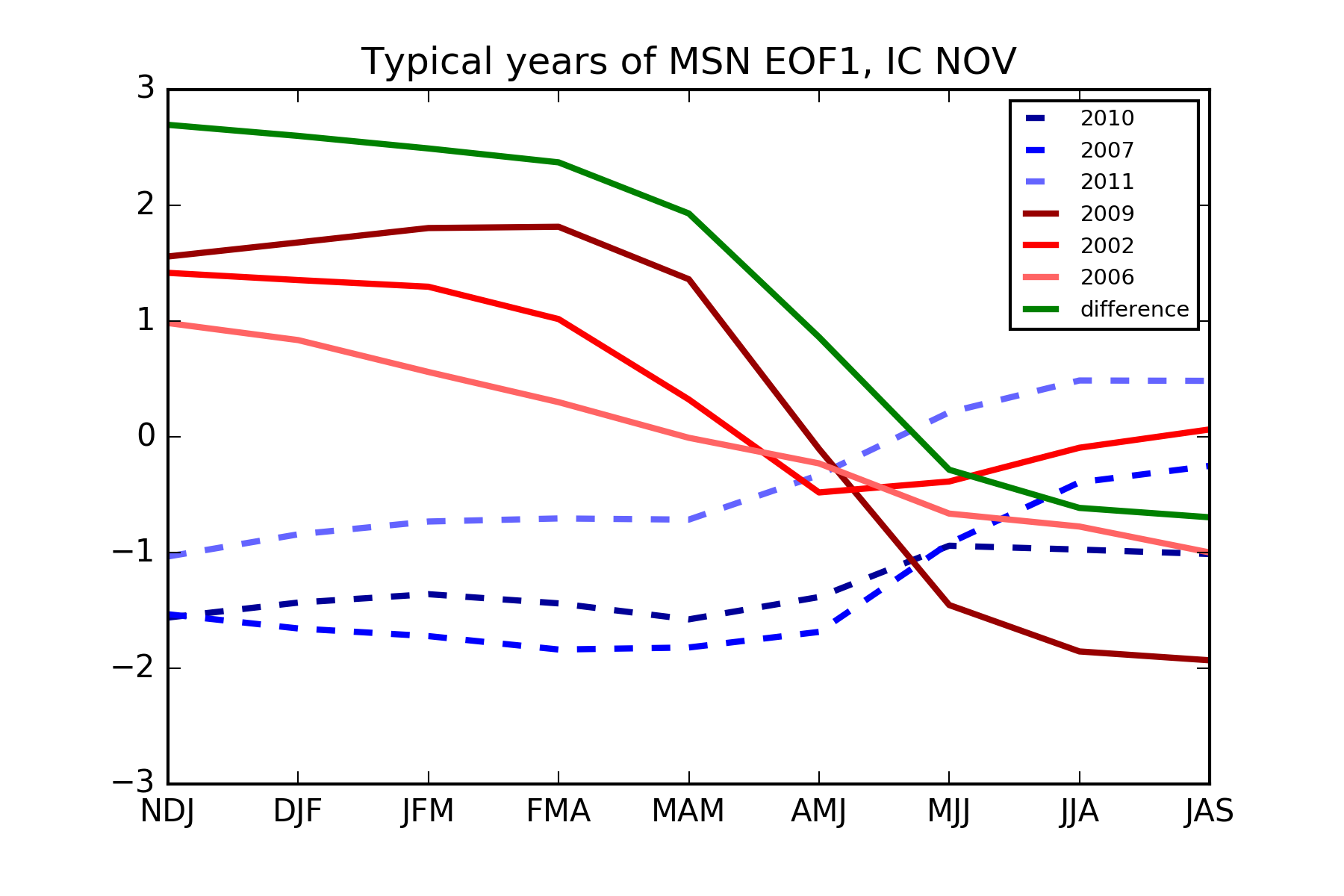

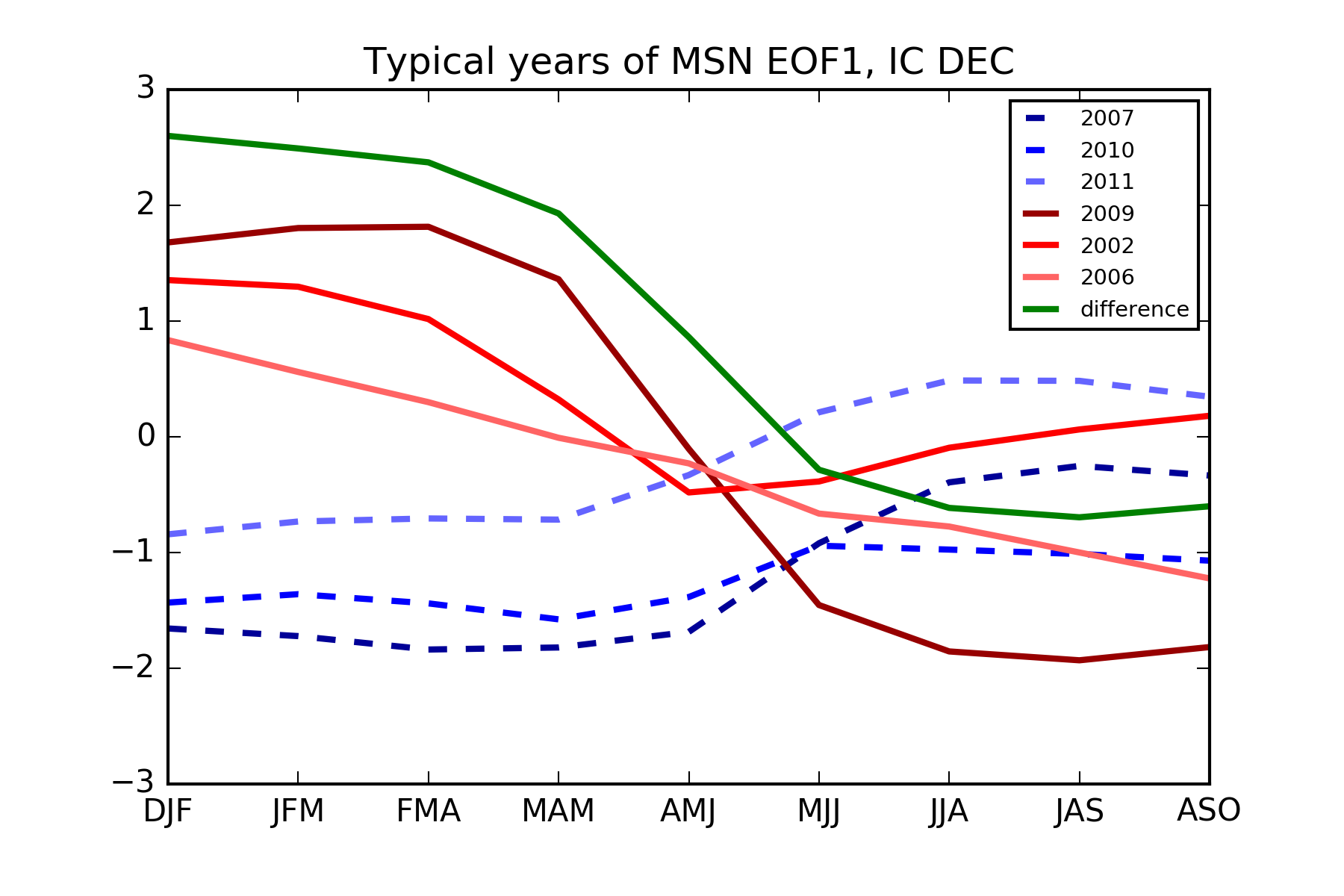


**Supplementary Figure 4** The observed evolution of three maximum positive PC1 years (solid lines) and three maximum negative PC1 years (dashed lines). The green line is the differences between the mean of three maximum positive PC1 years minus the mean of three maximum negative PC1 years in observation. Figure is generated by Python (https://www.python.org/)


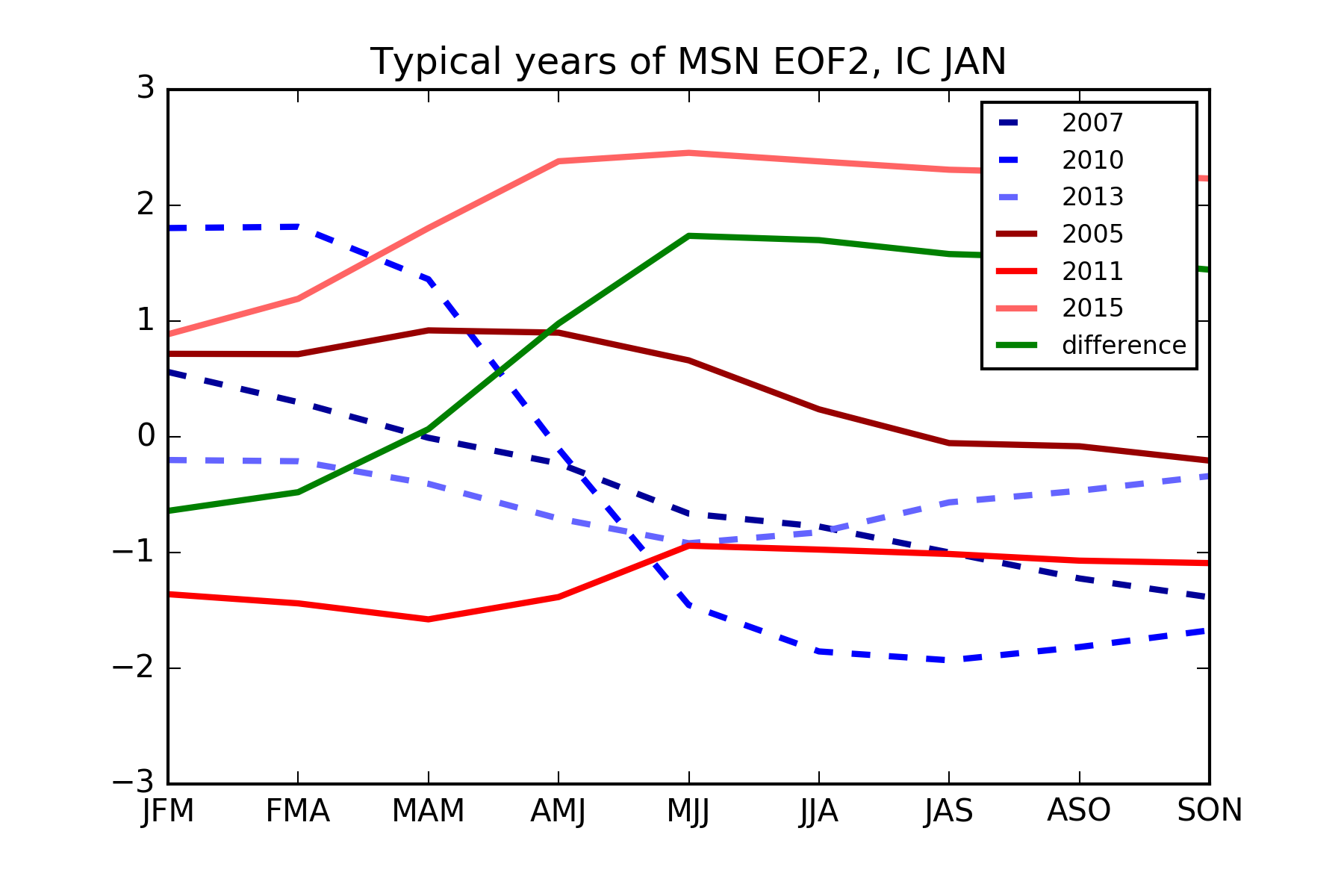

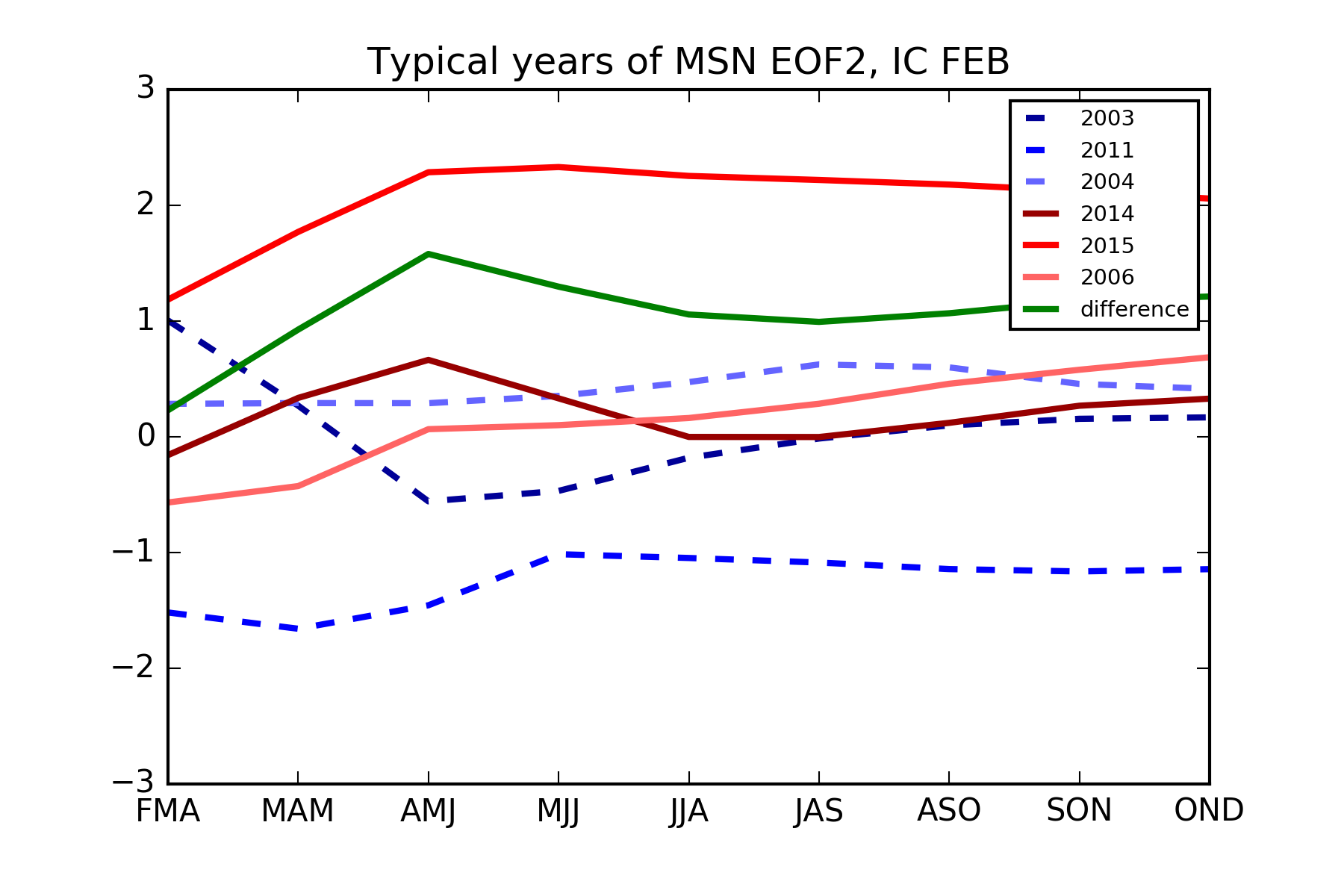

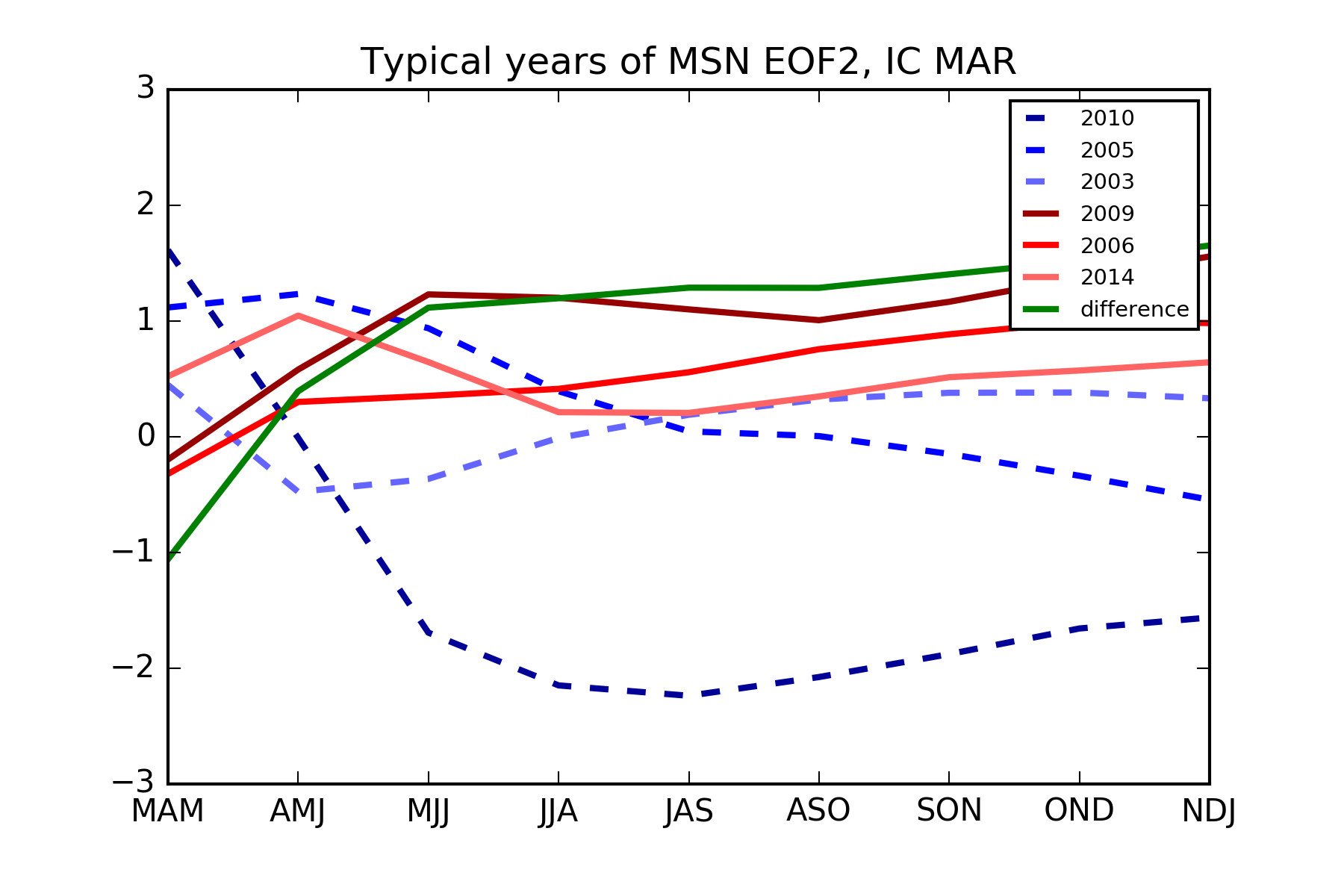

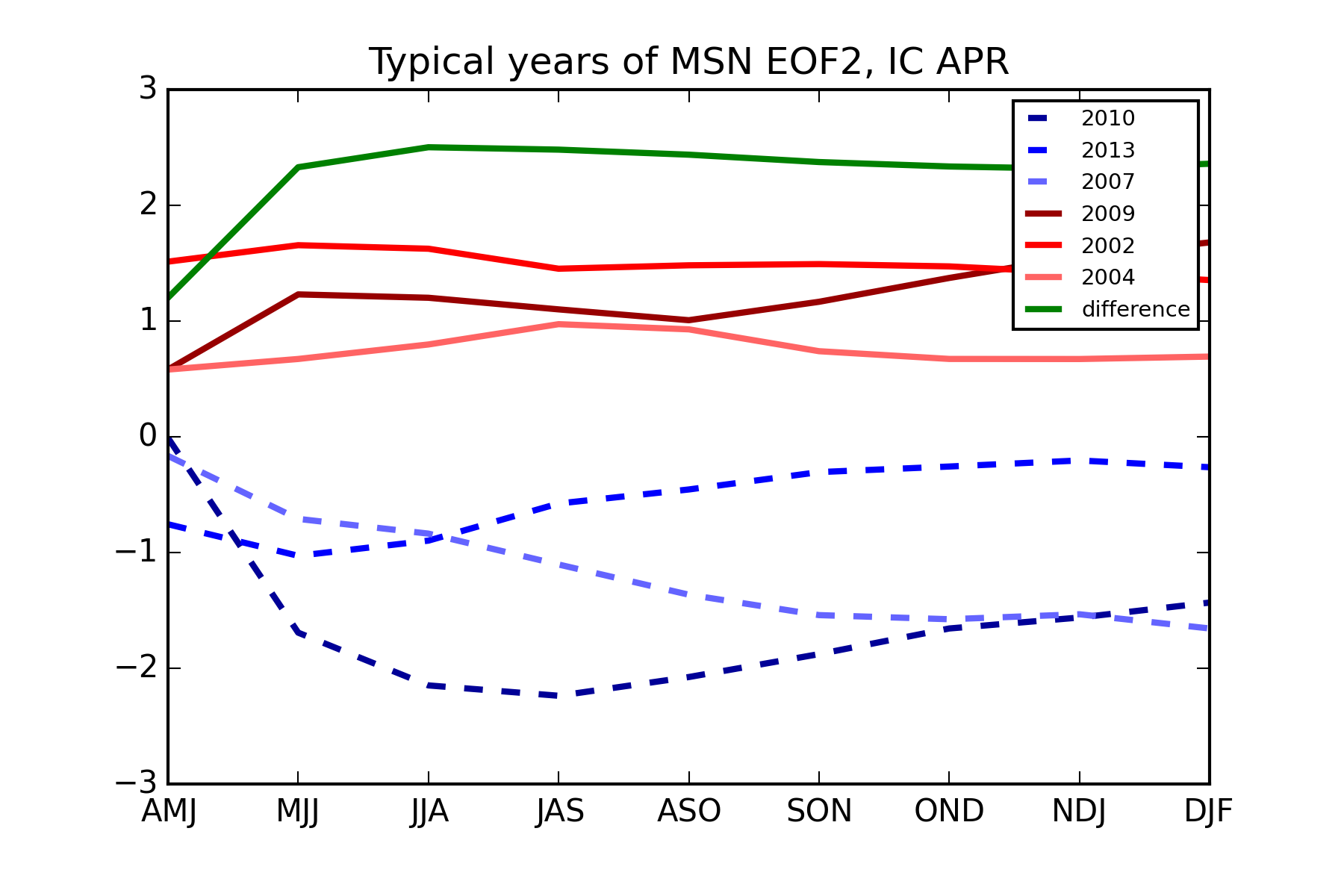

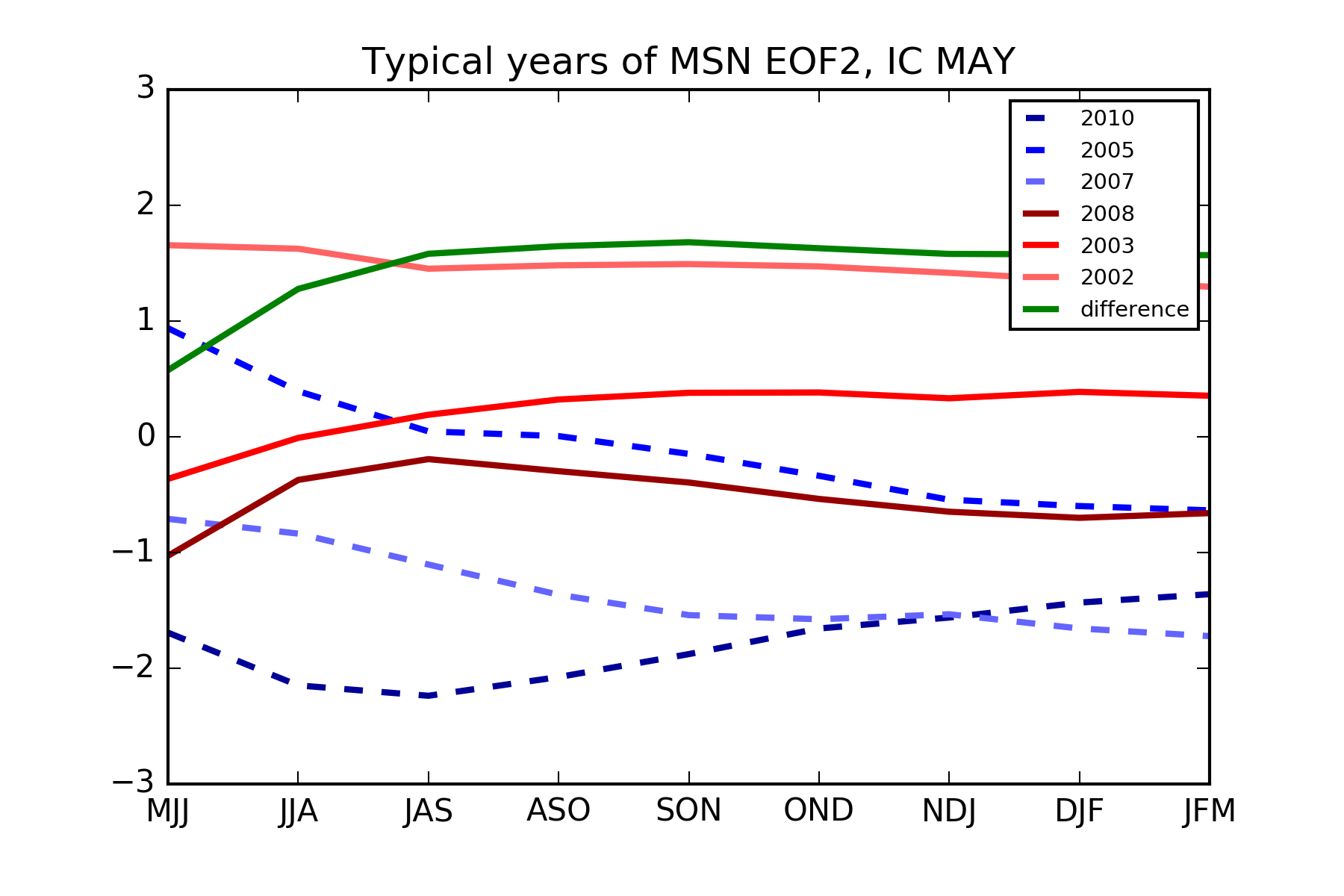

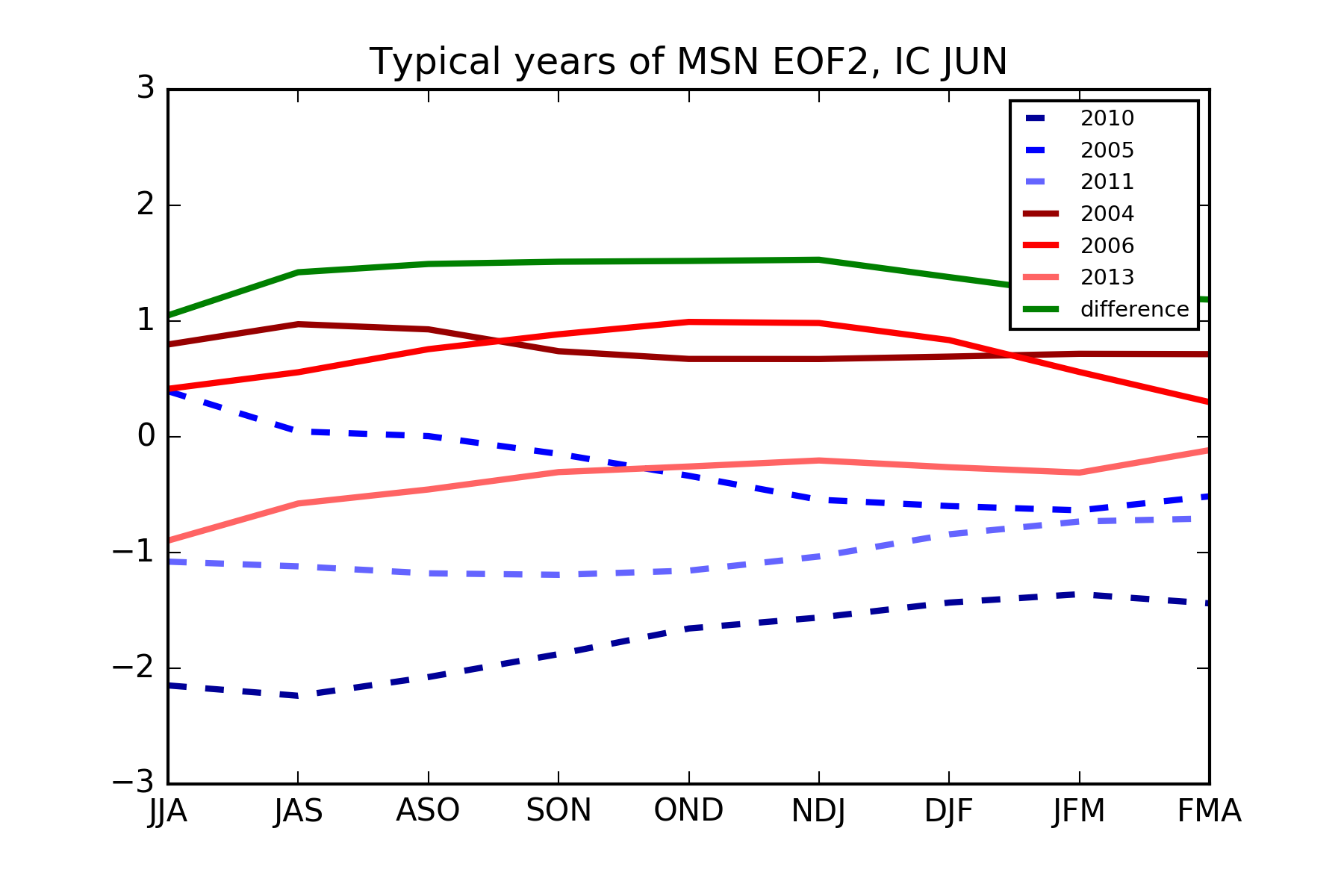

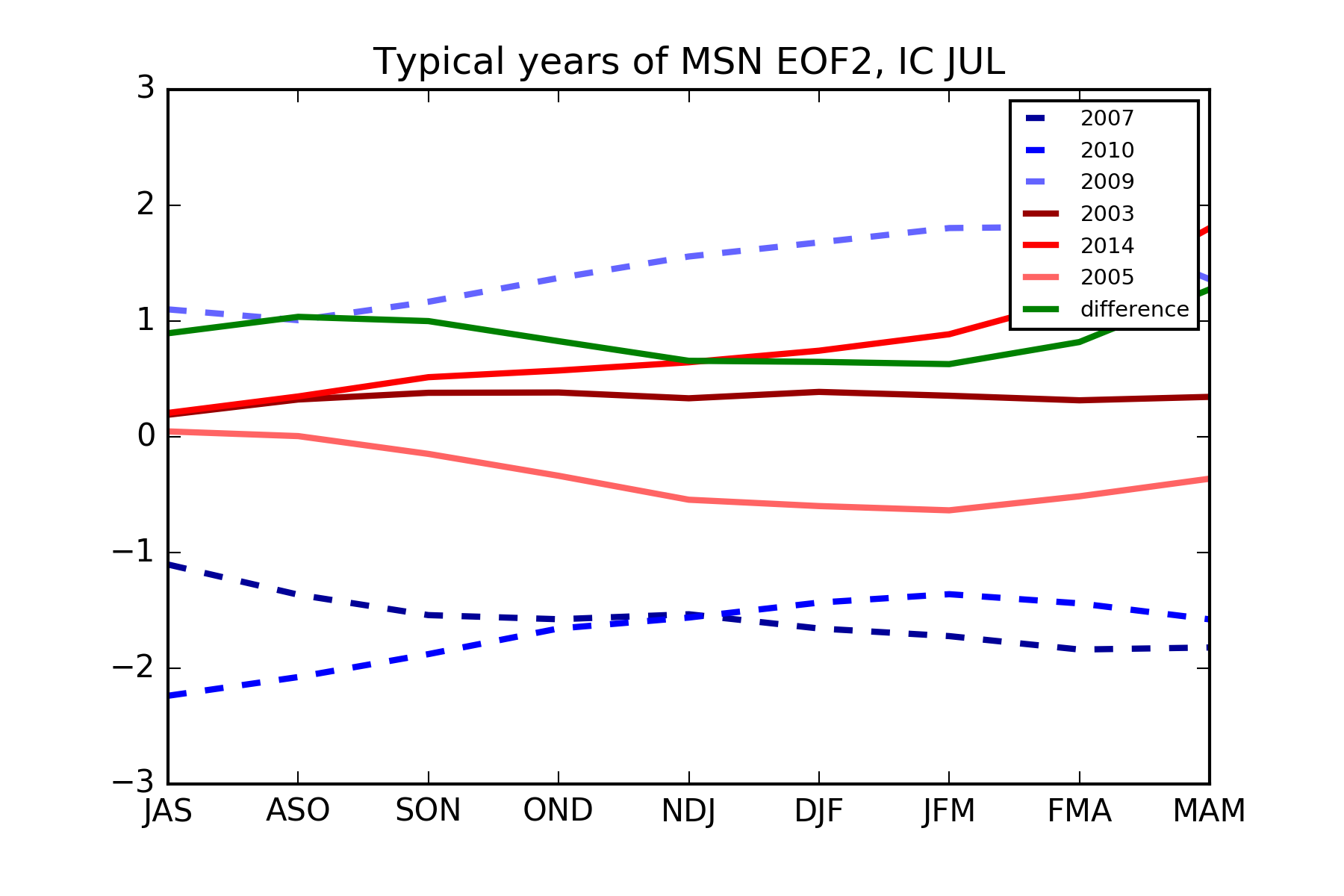

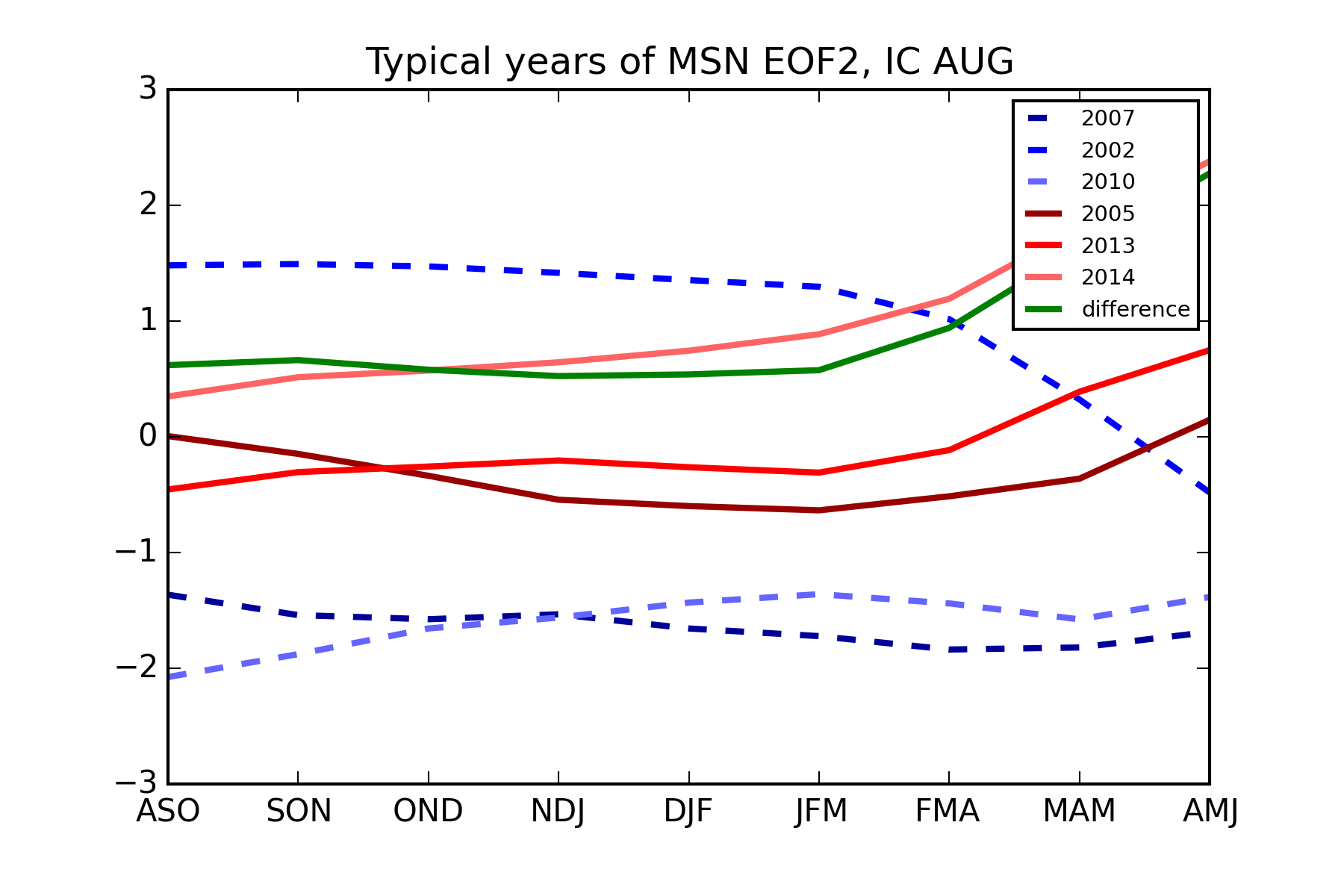

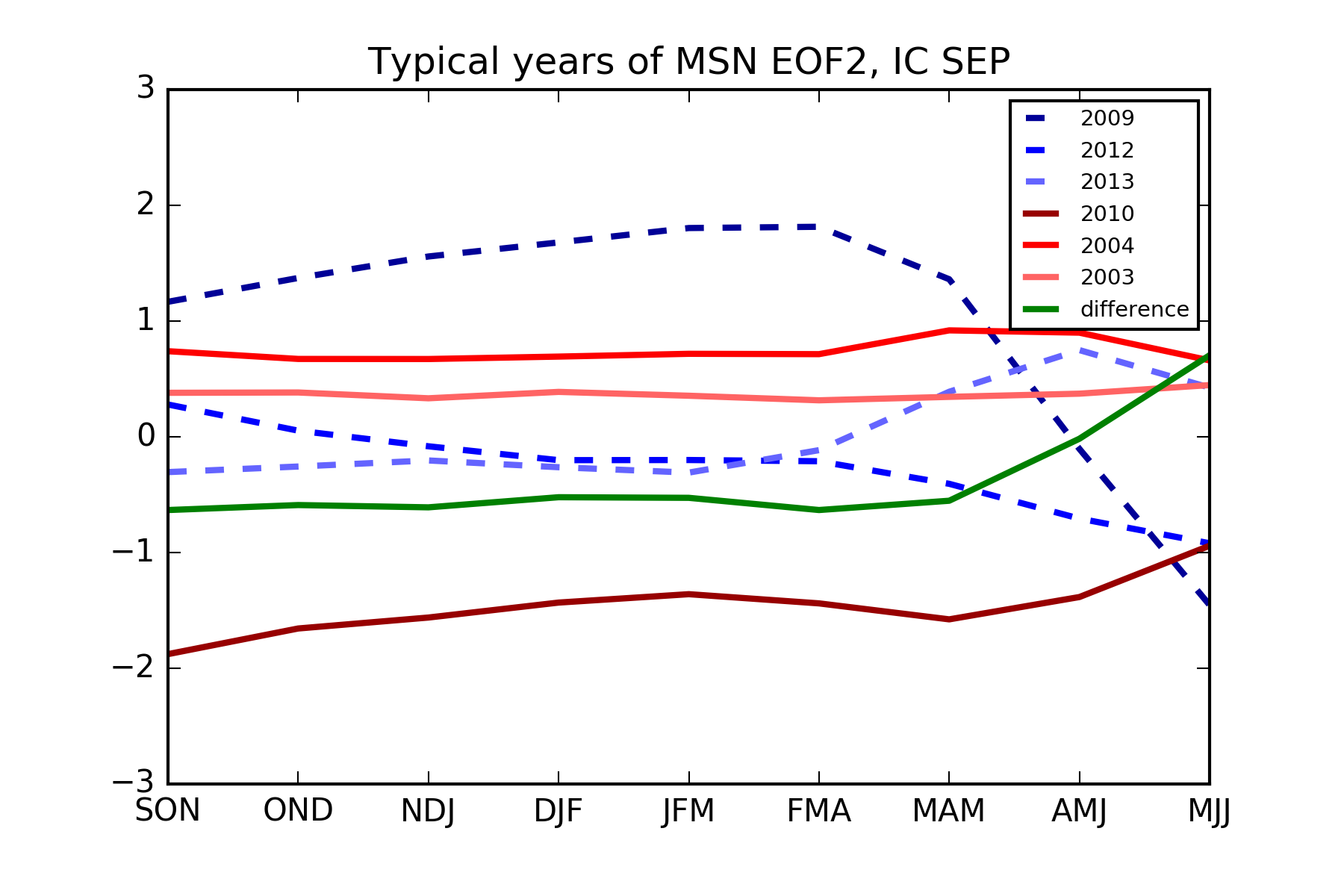

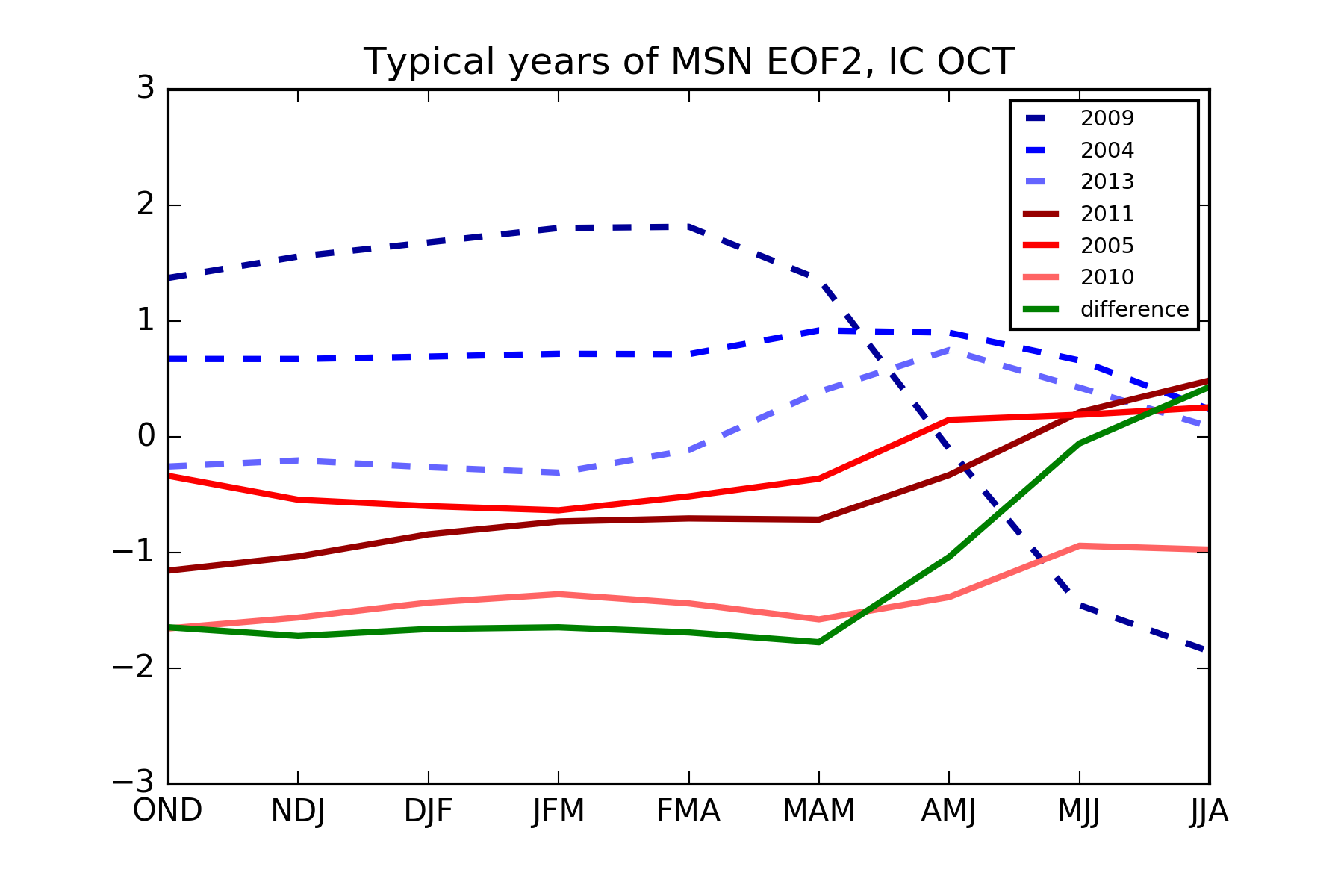

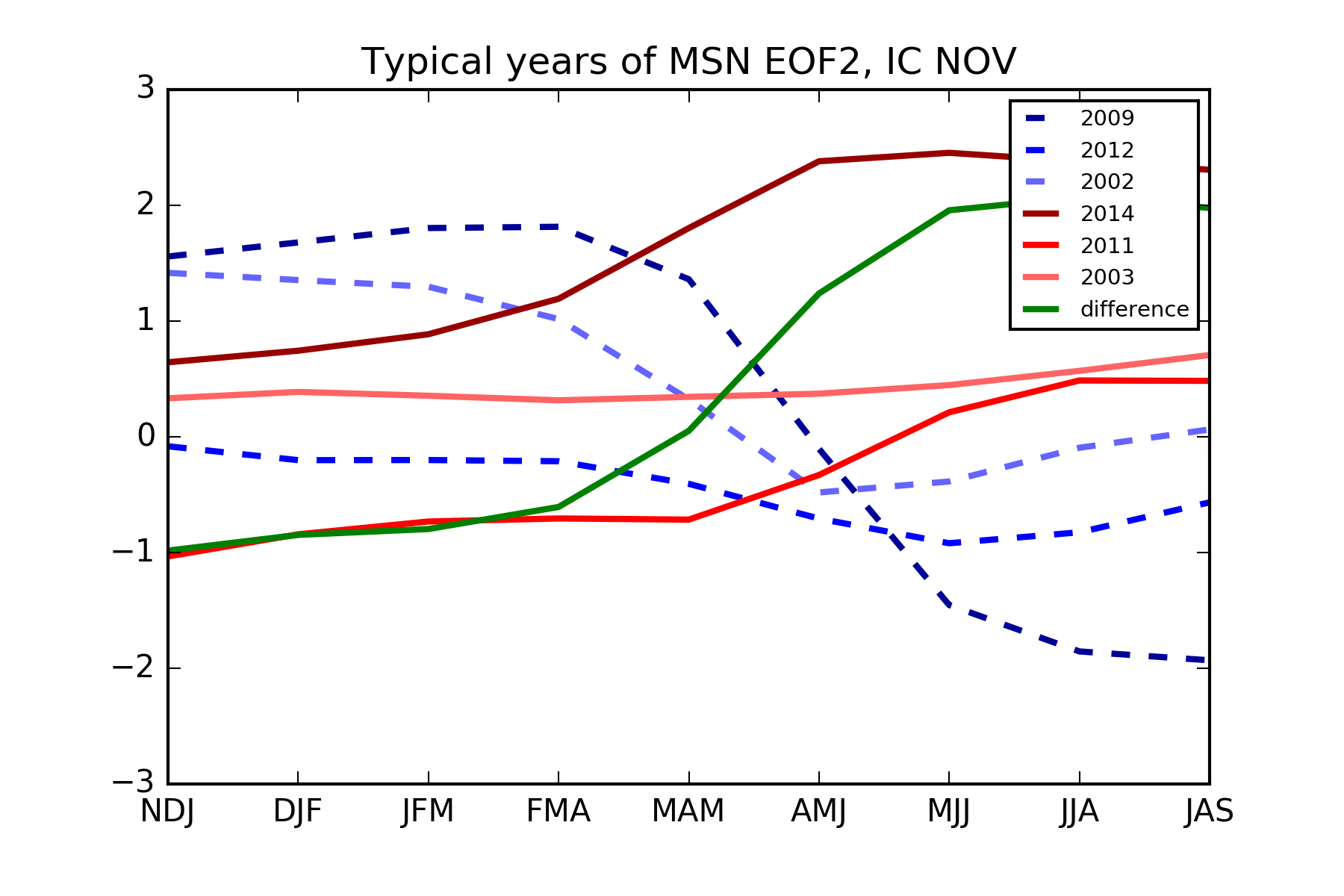

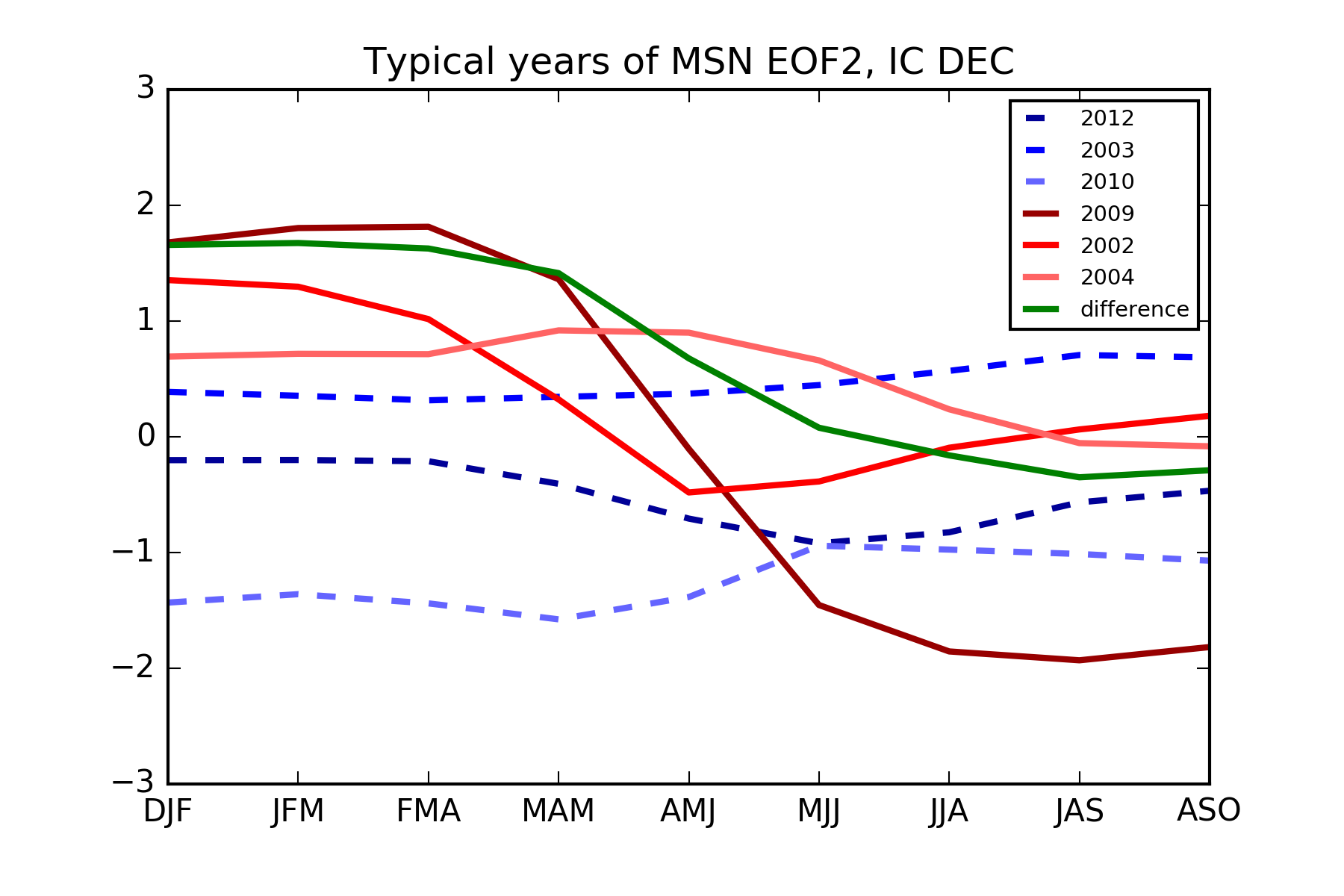


**Supplementary Figure 5** Same as supplementary Fig. 4, but for the second MSN EOF mode. Figure is generated by Python (https://www.python.org/)


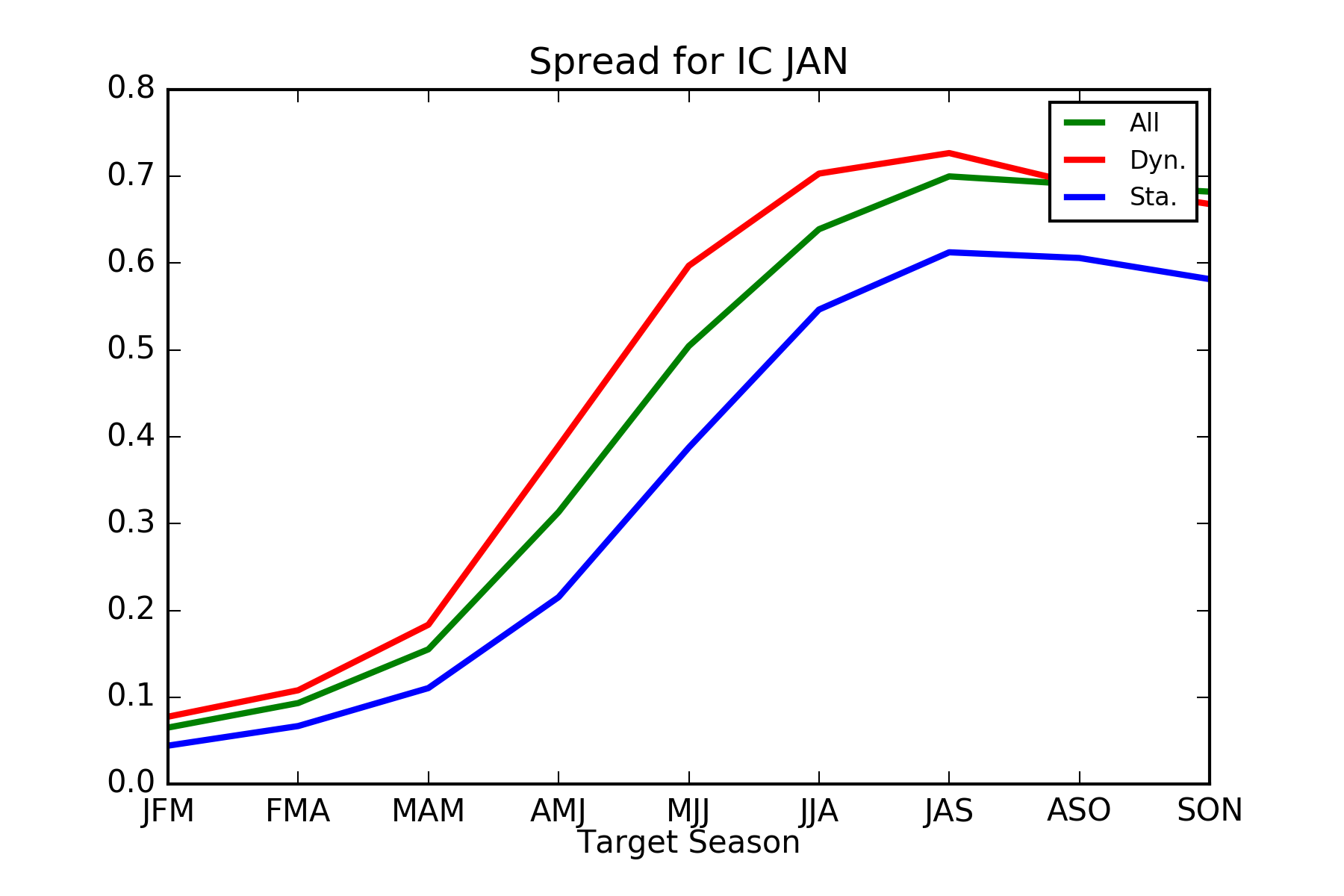

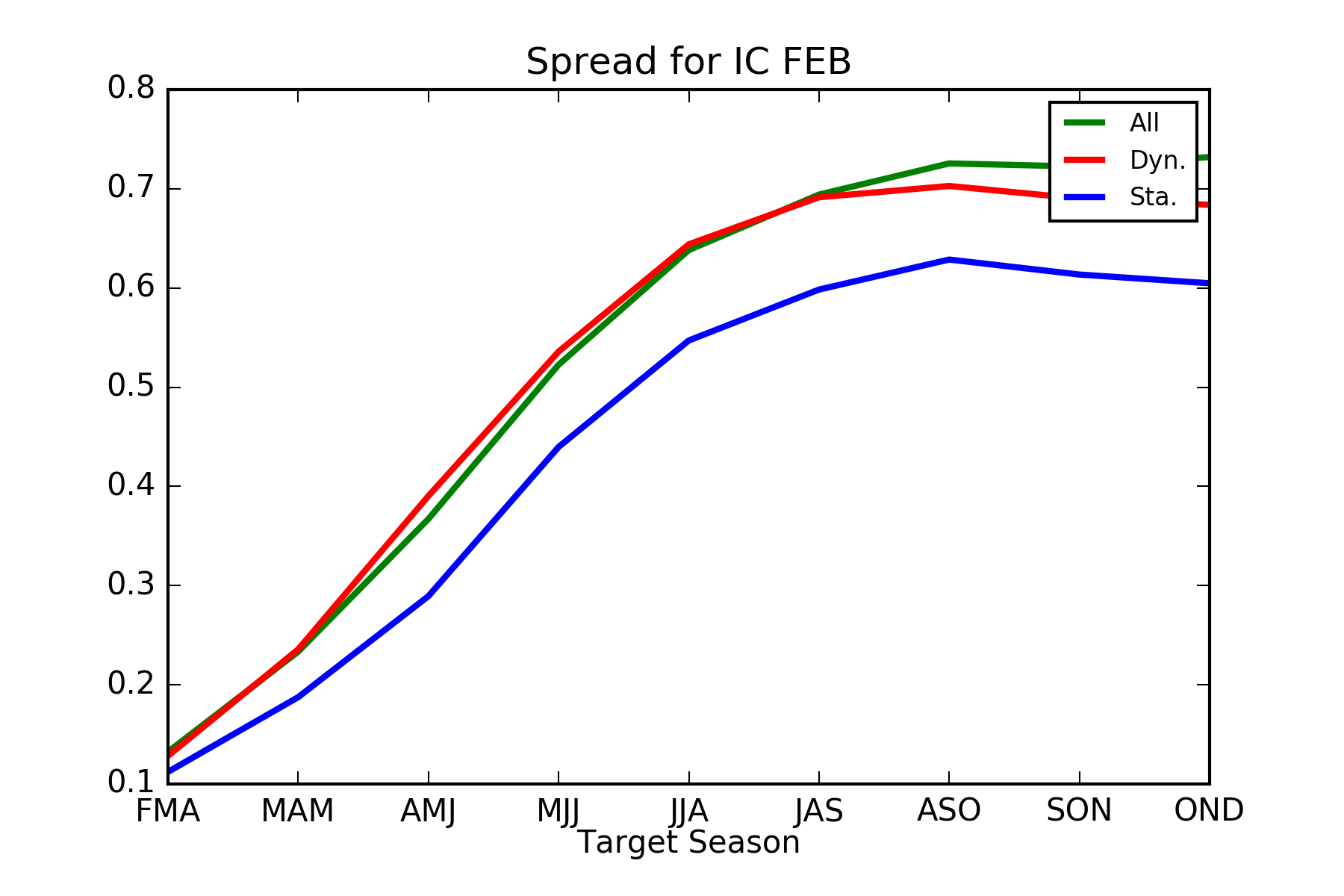

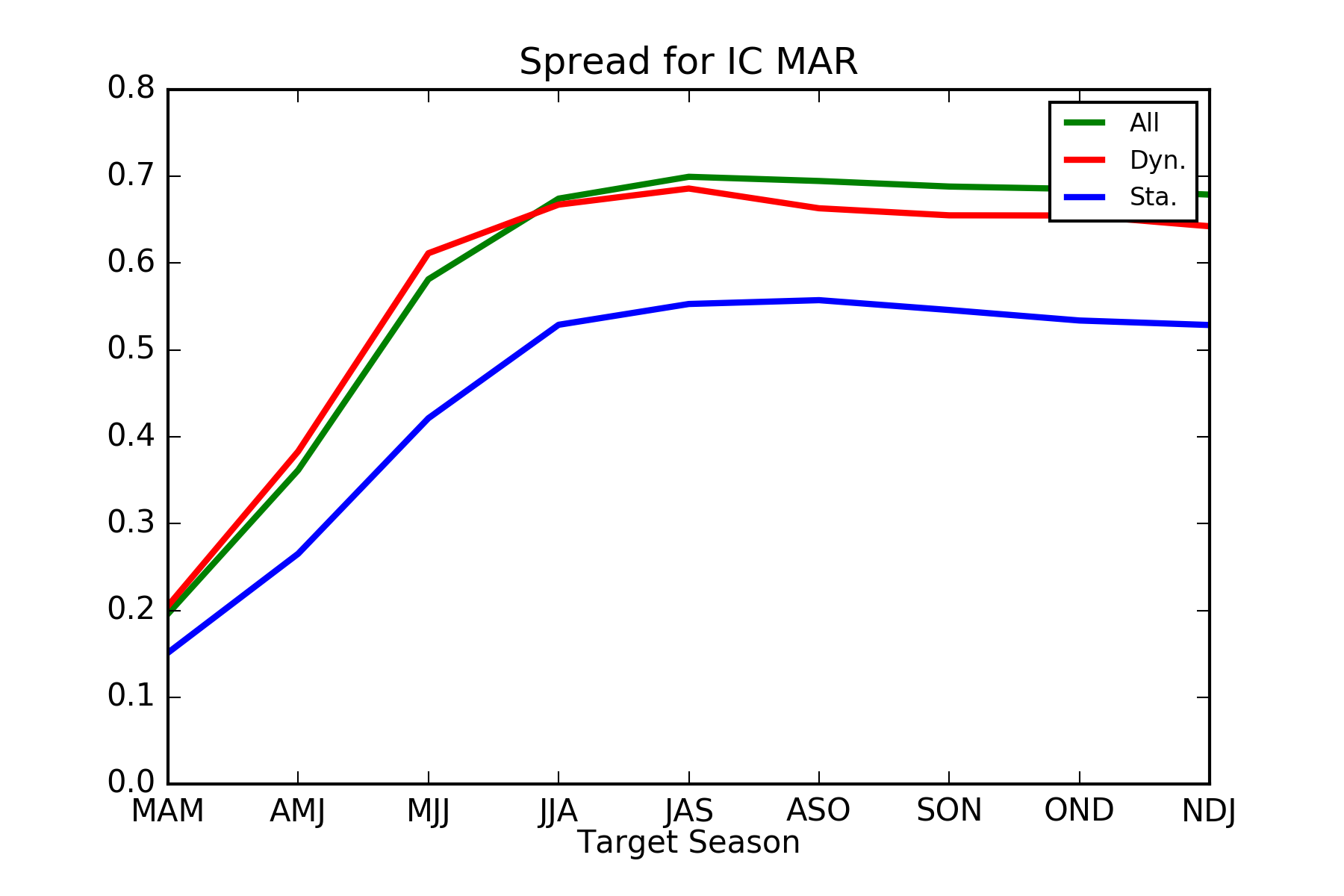

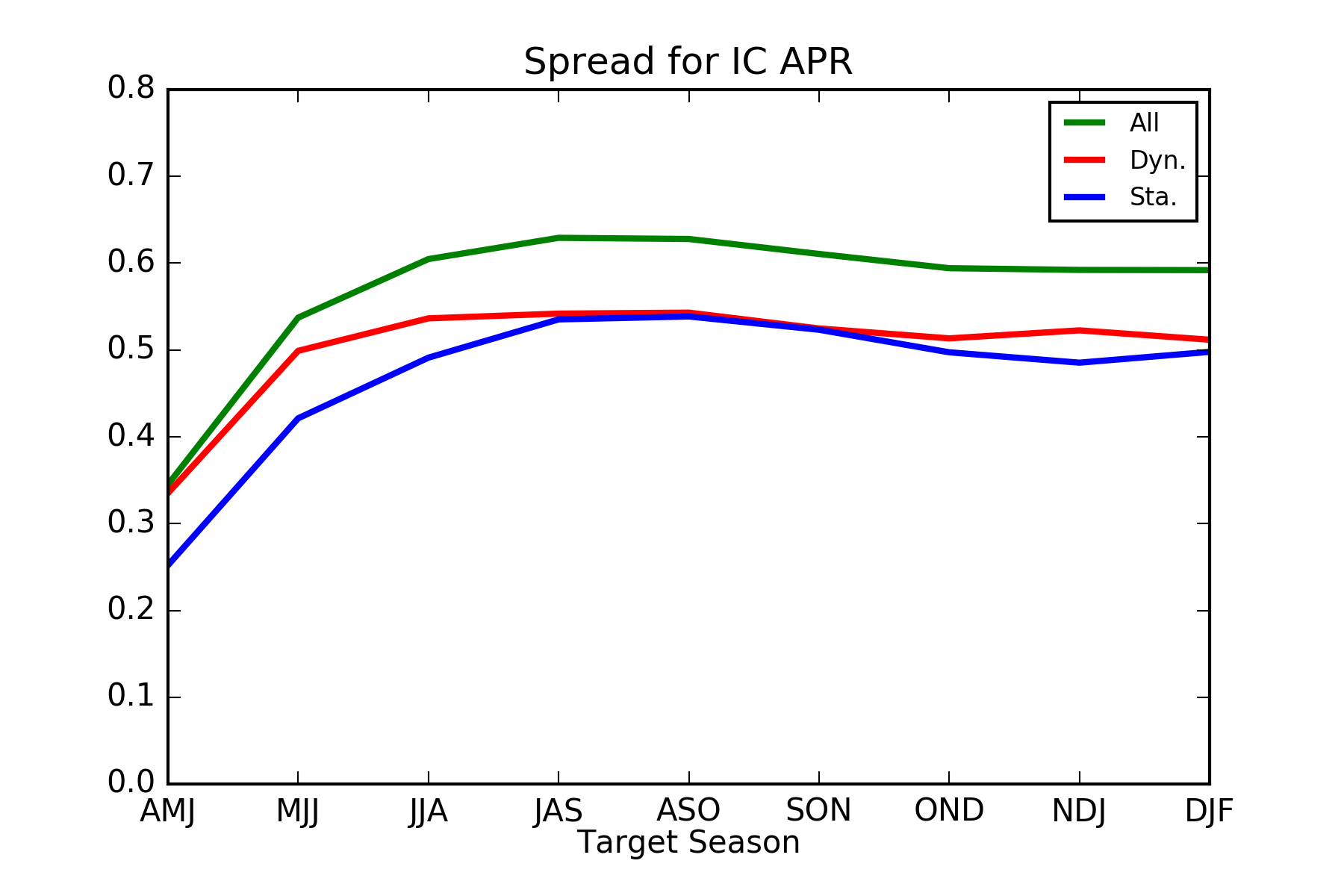

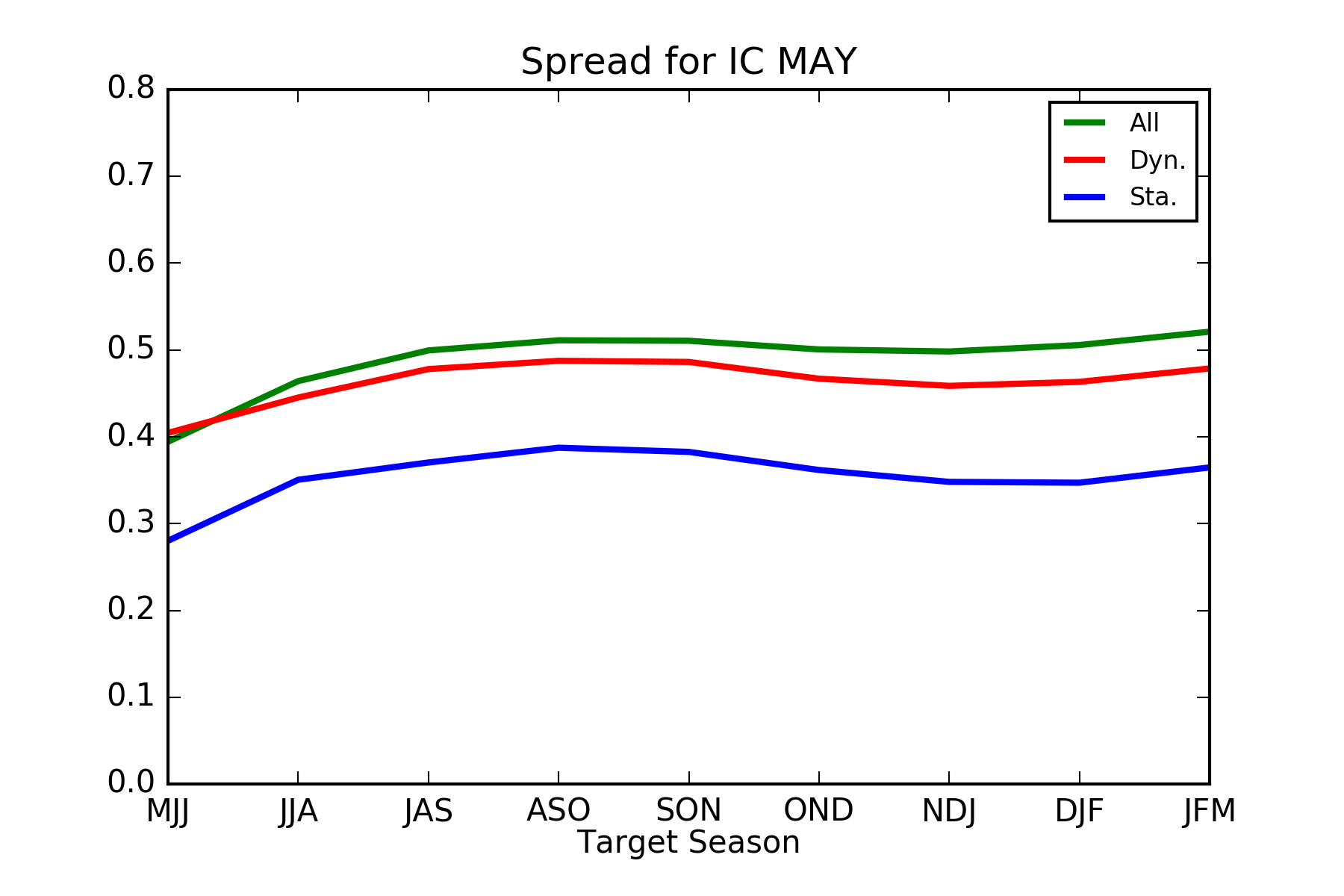

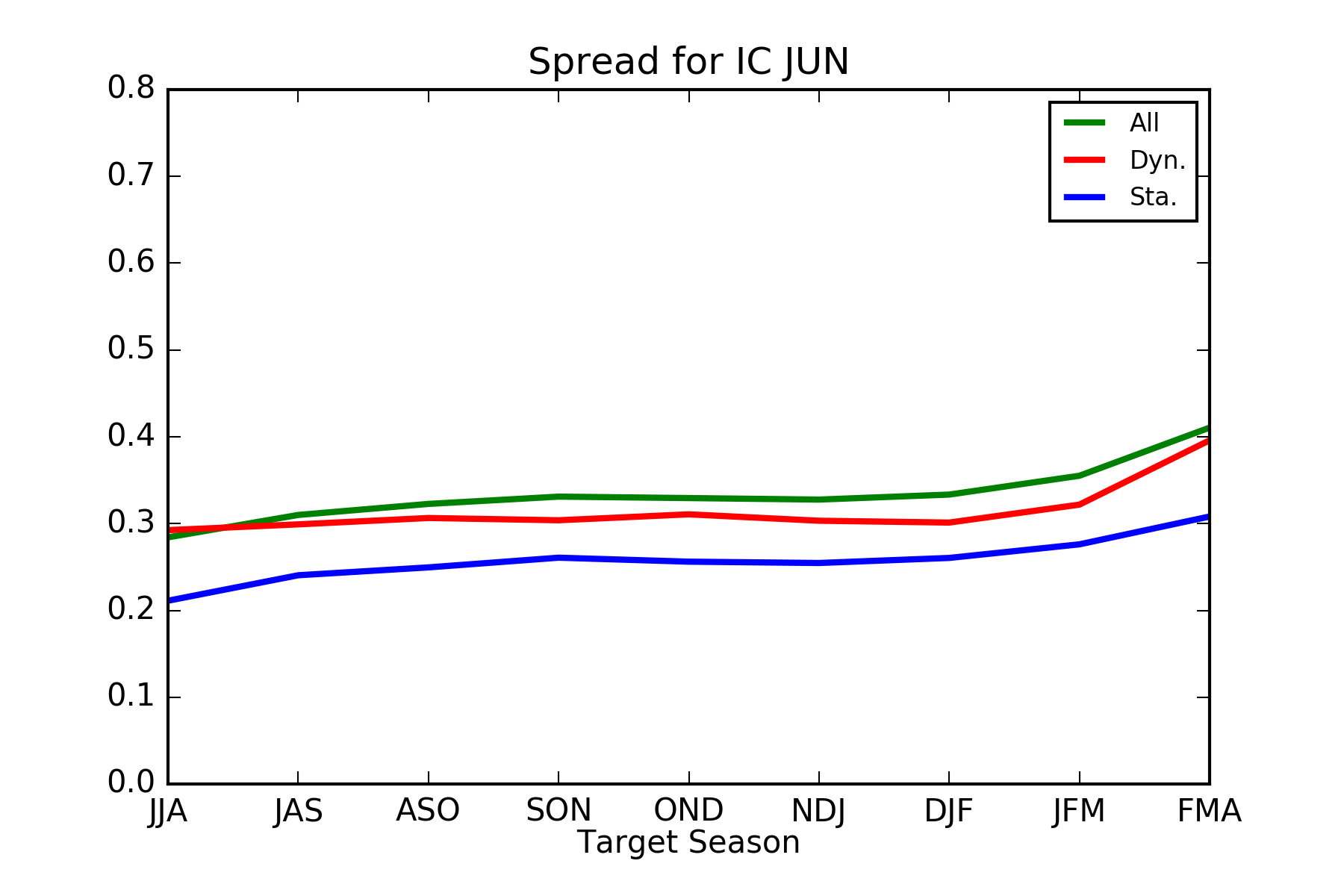

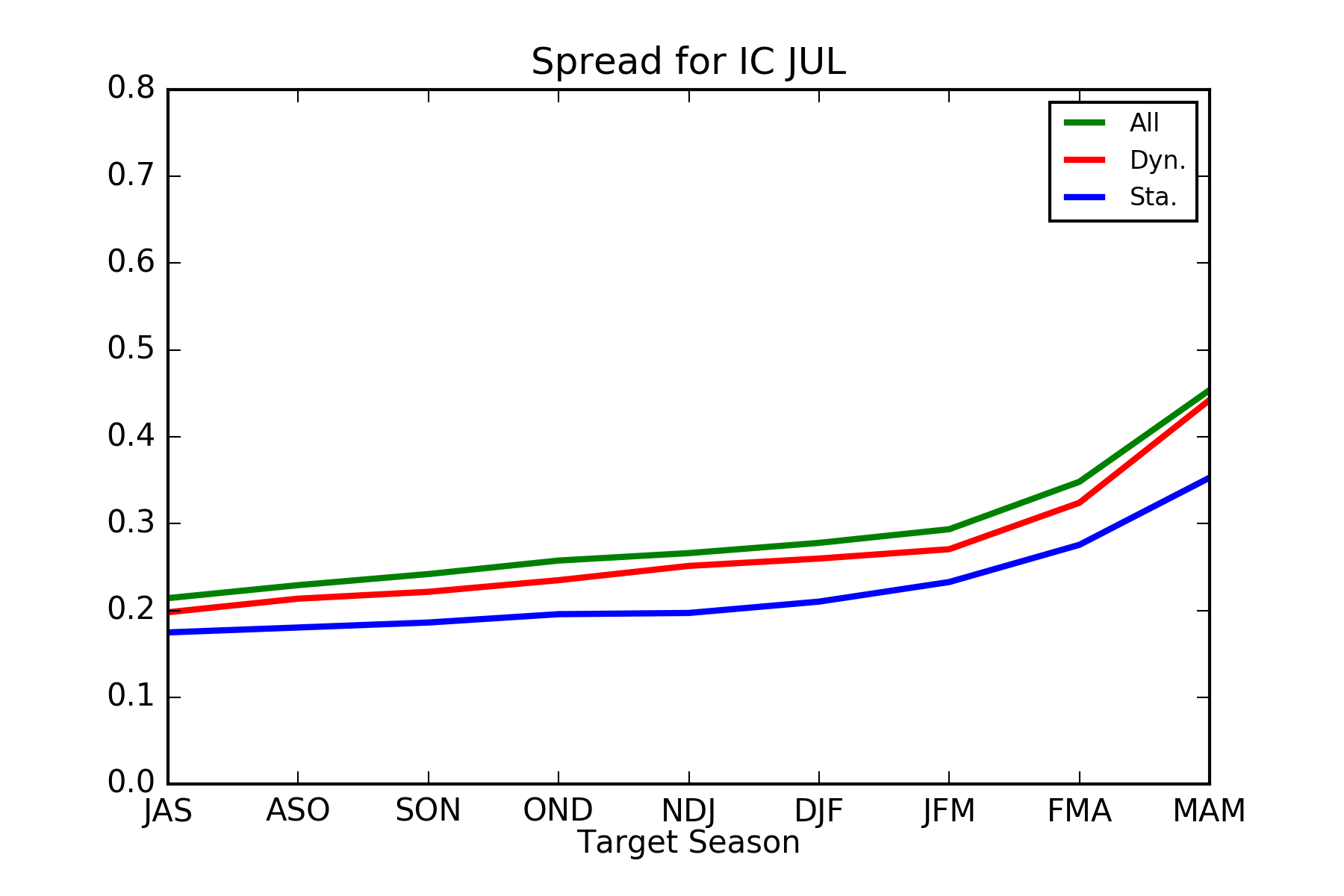

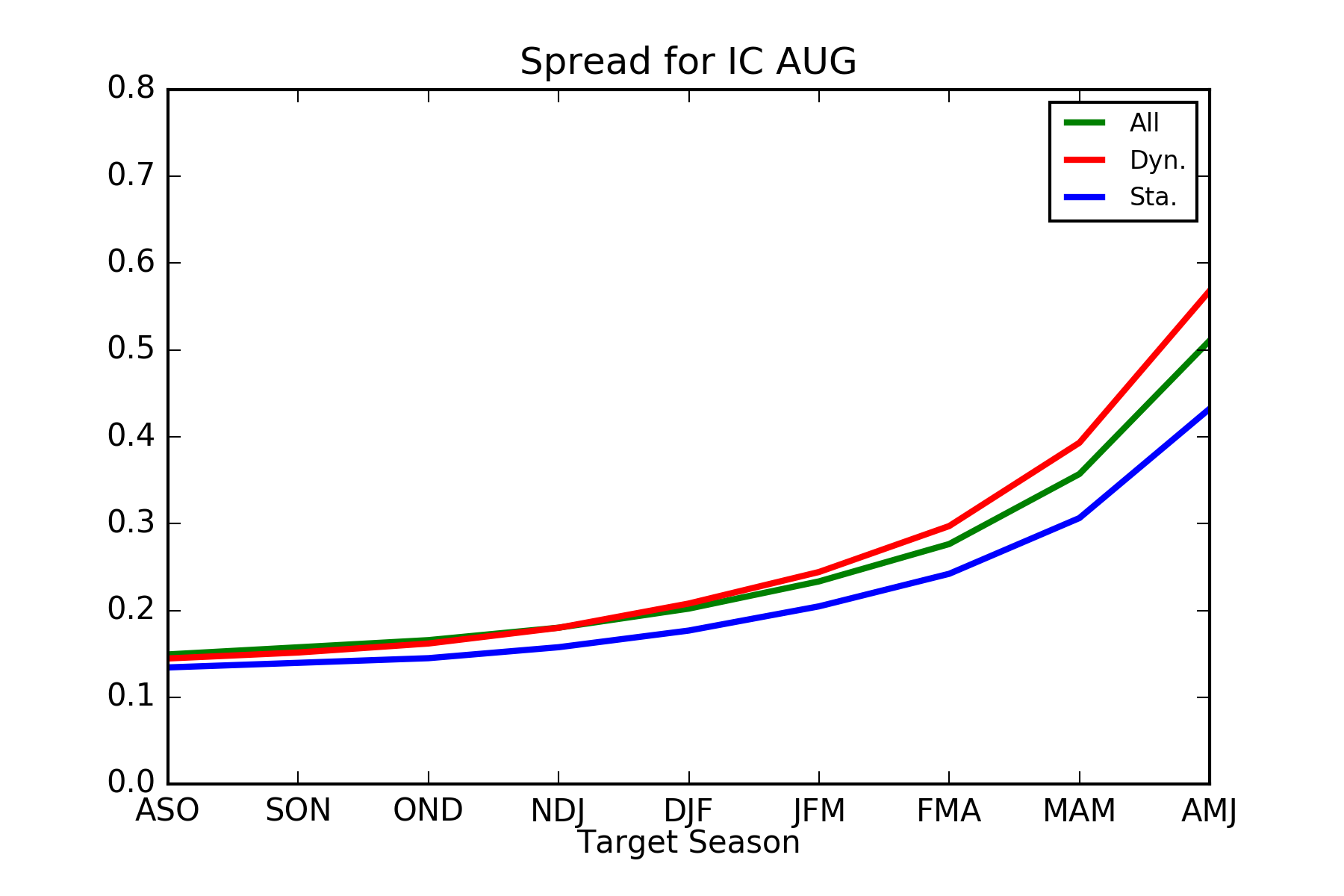

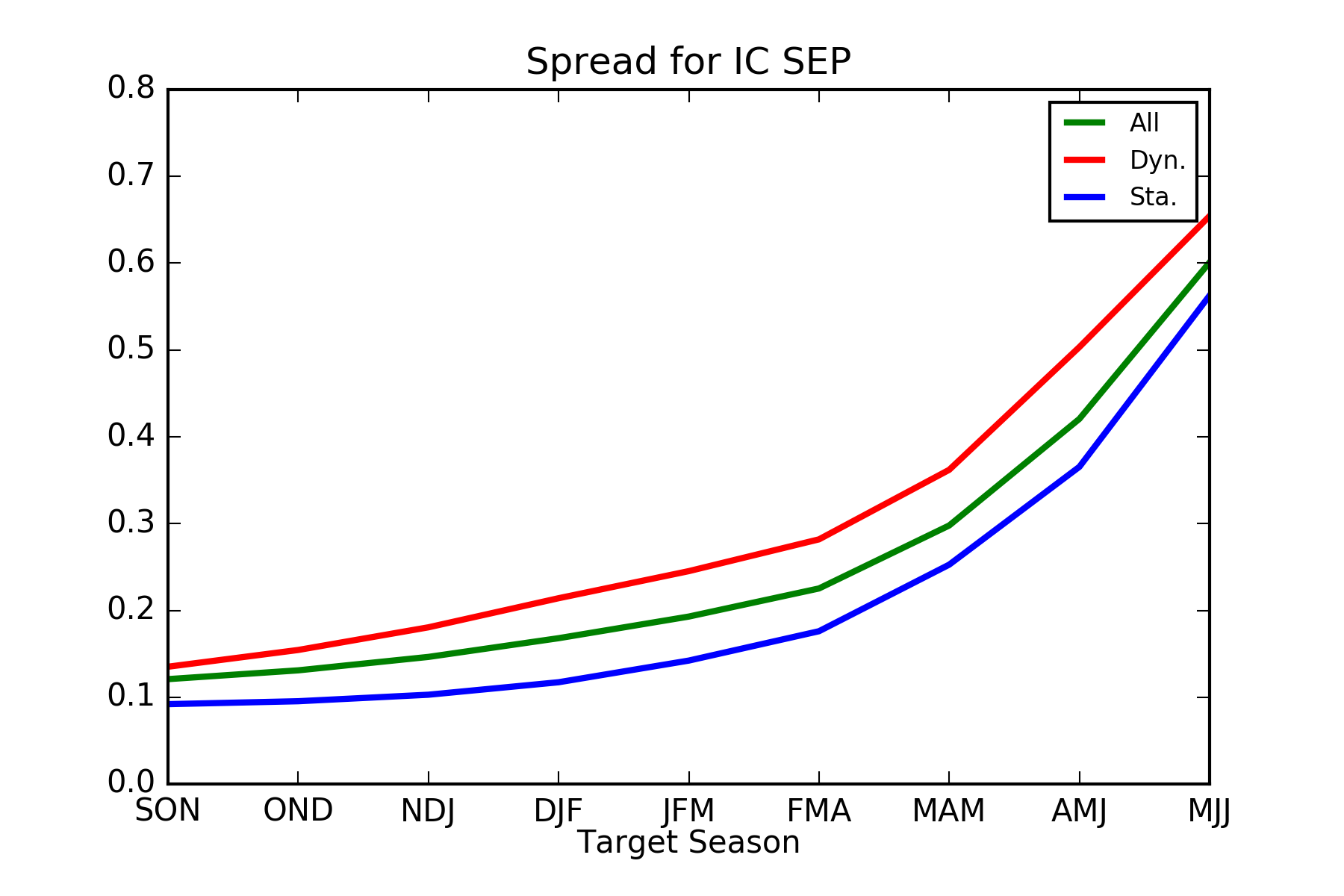

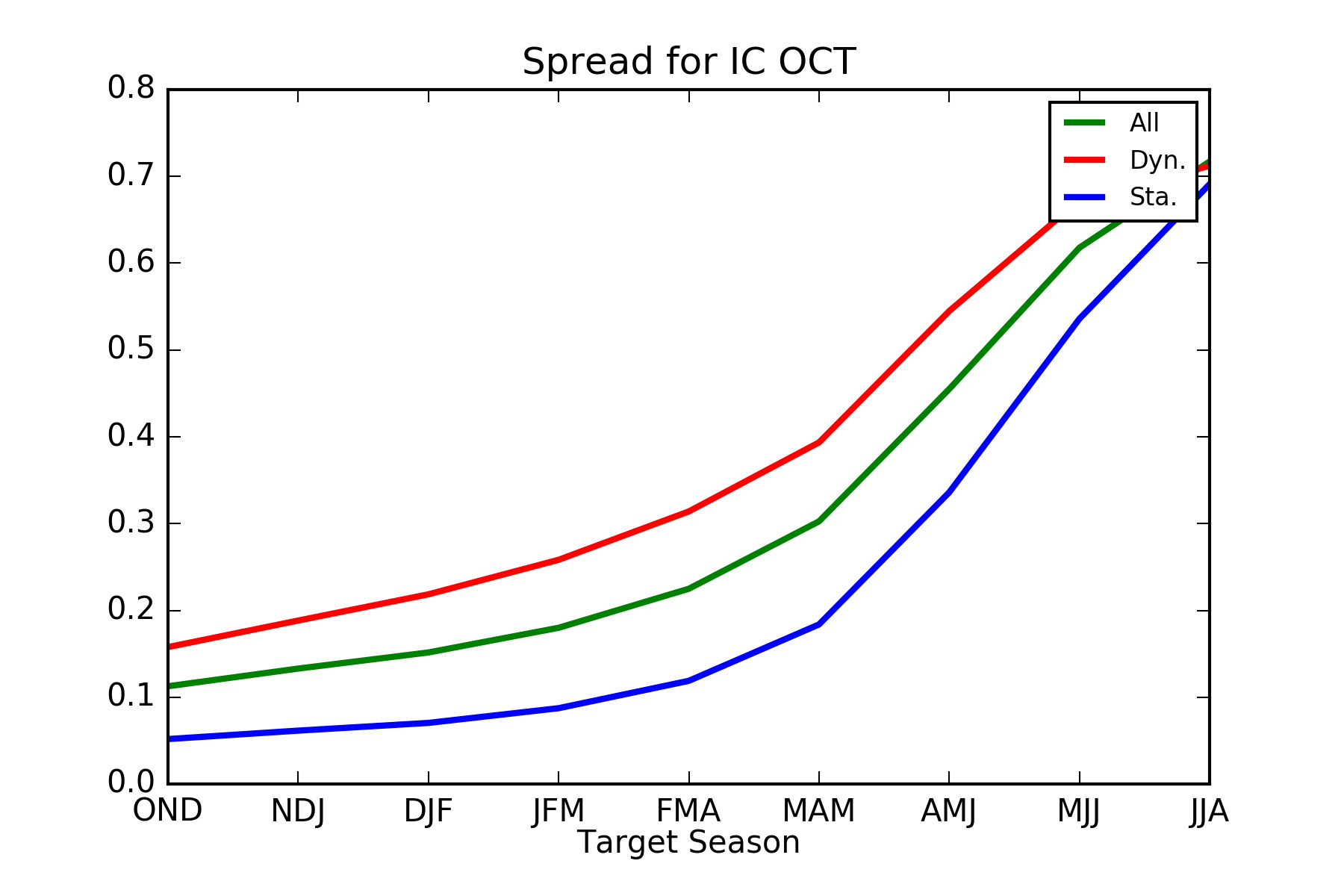

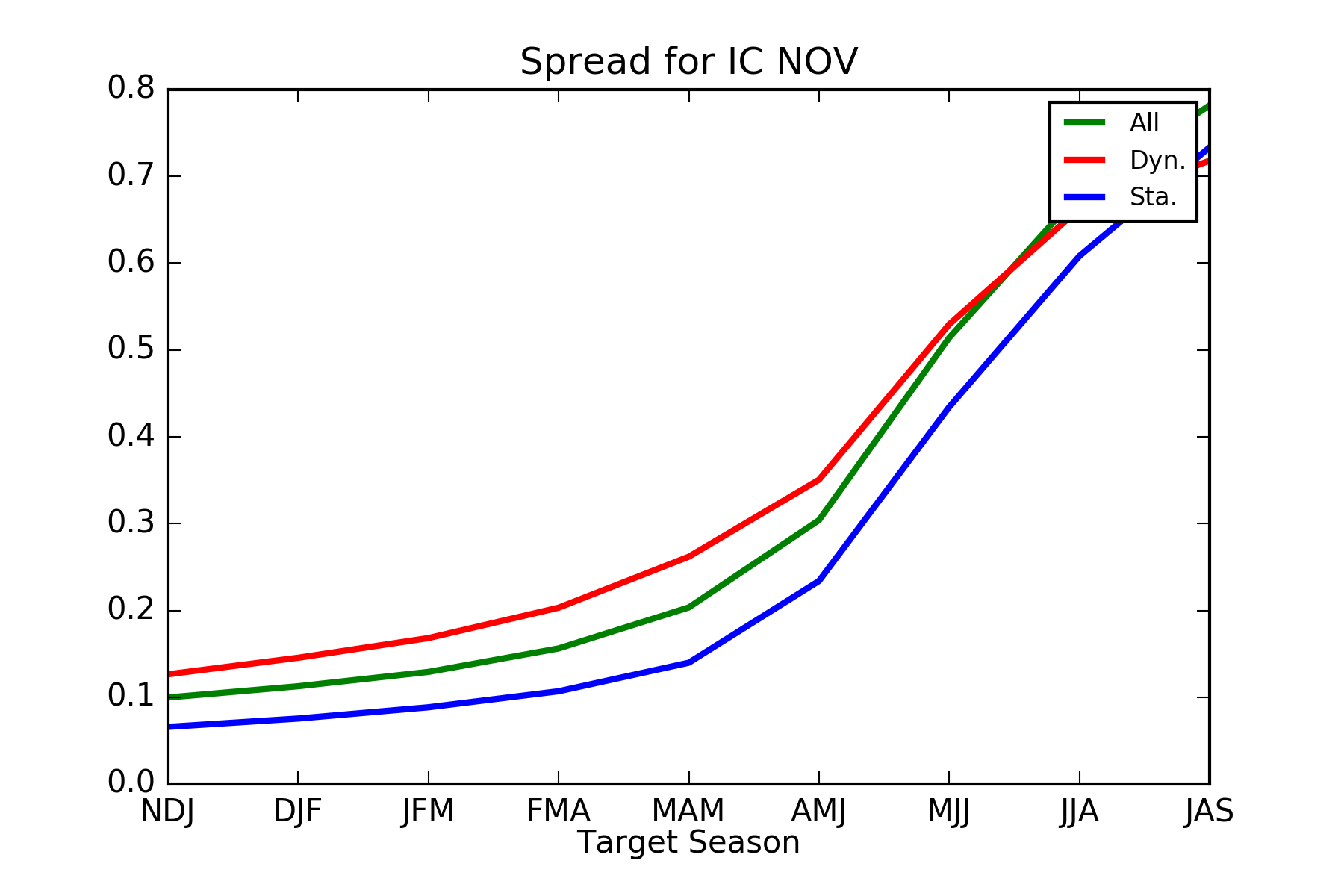

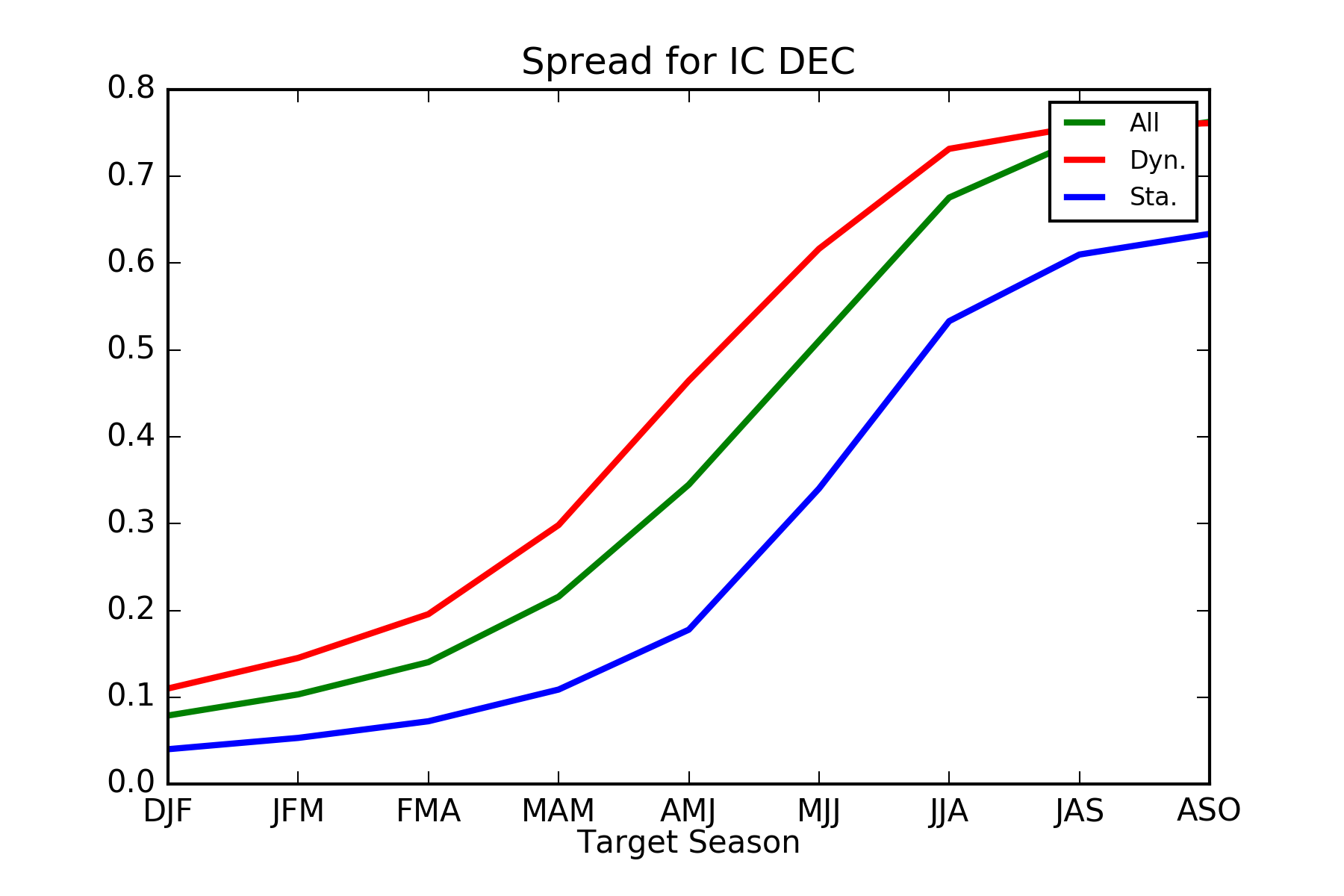


**Supplementary Figure 6** The spread of all models (green lines), dynamical models only (red lines) and statistical models only (blue lines) for IC in each month.


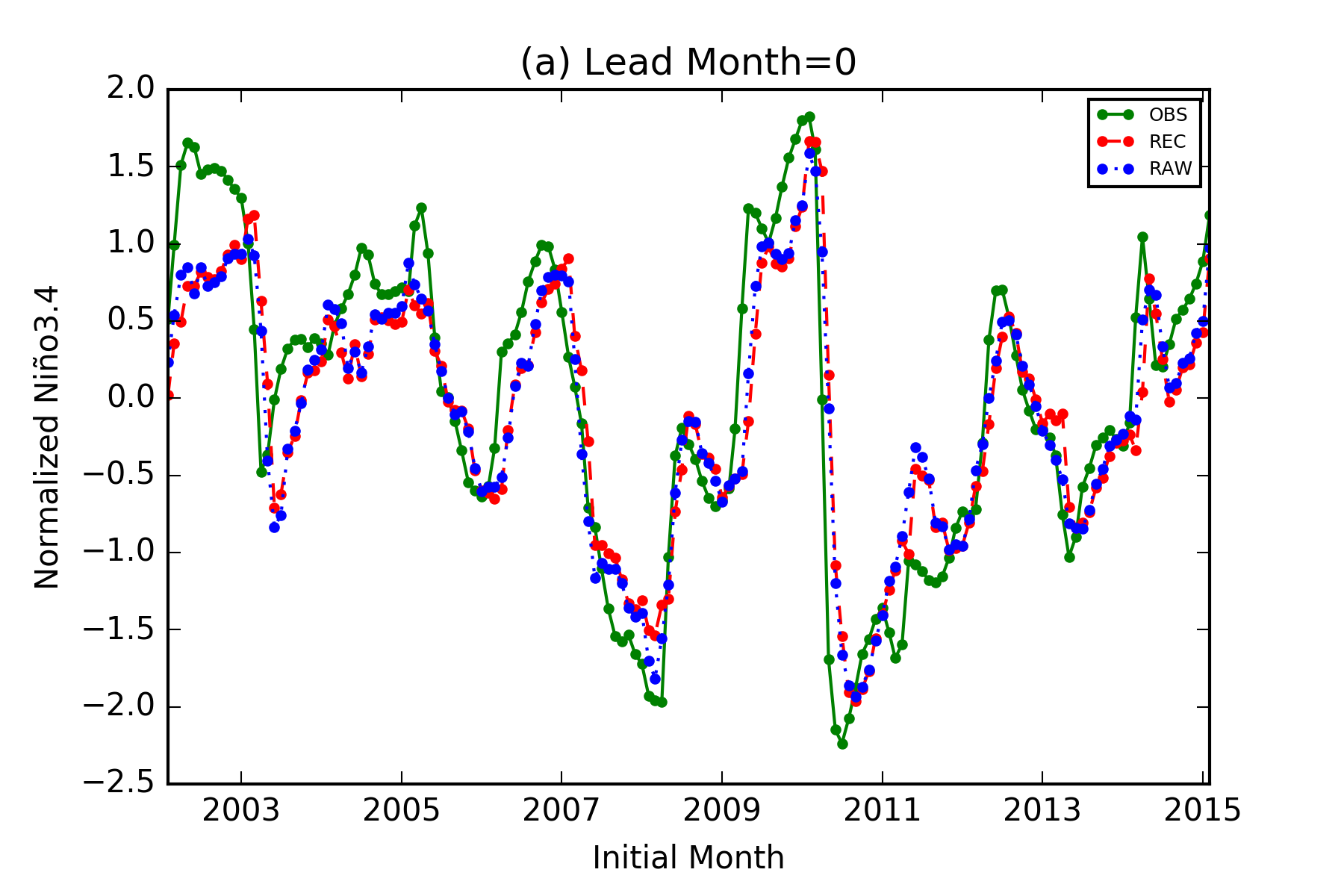

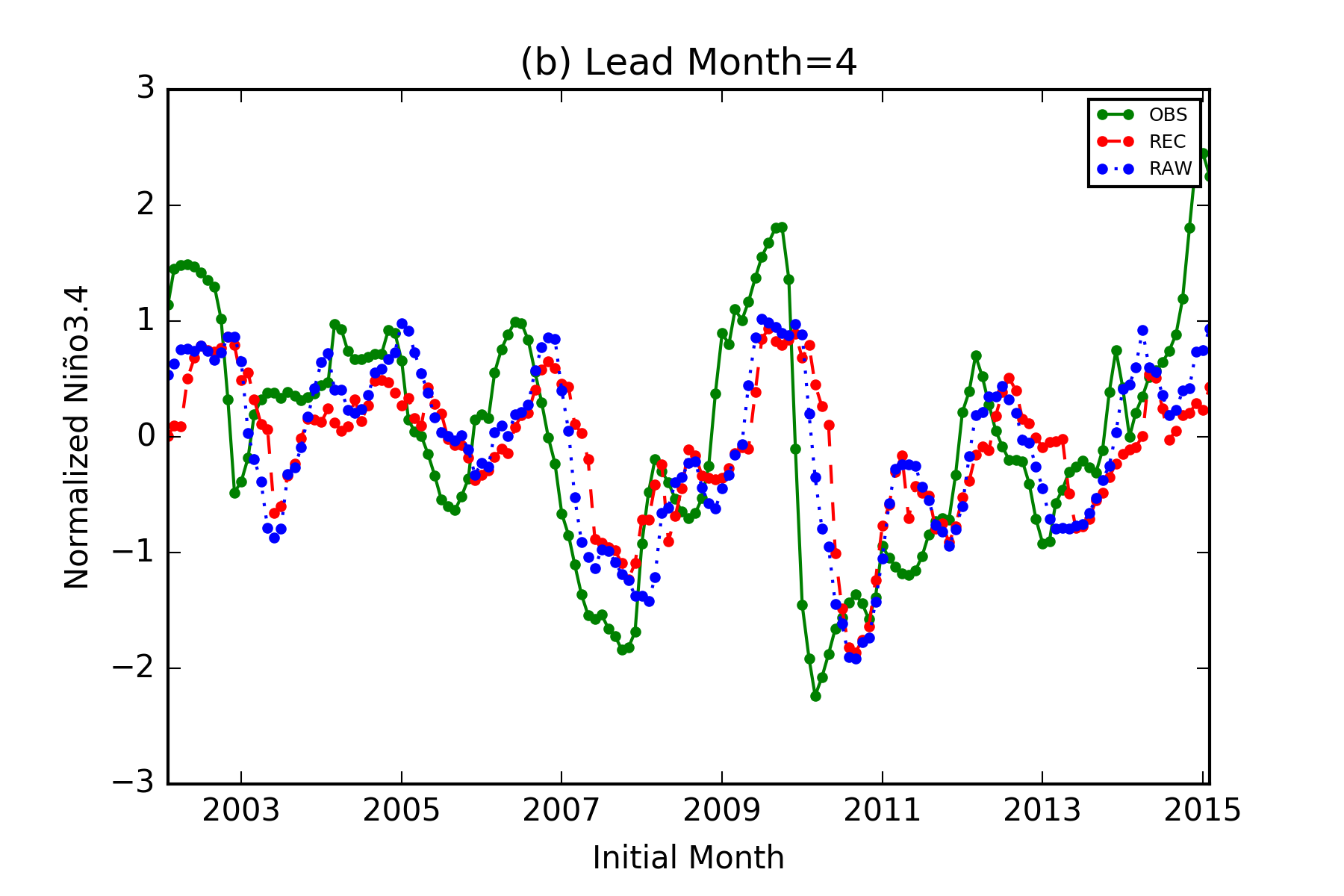

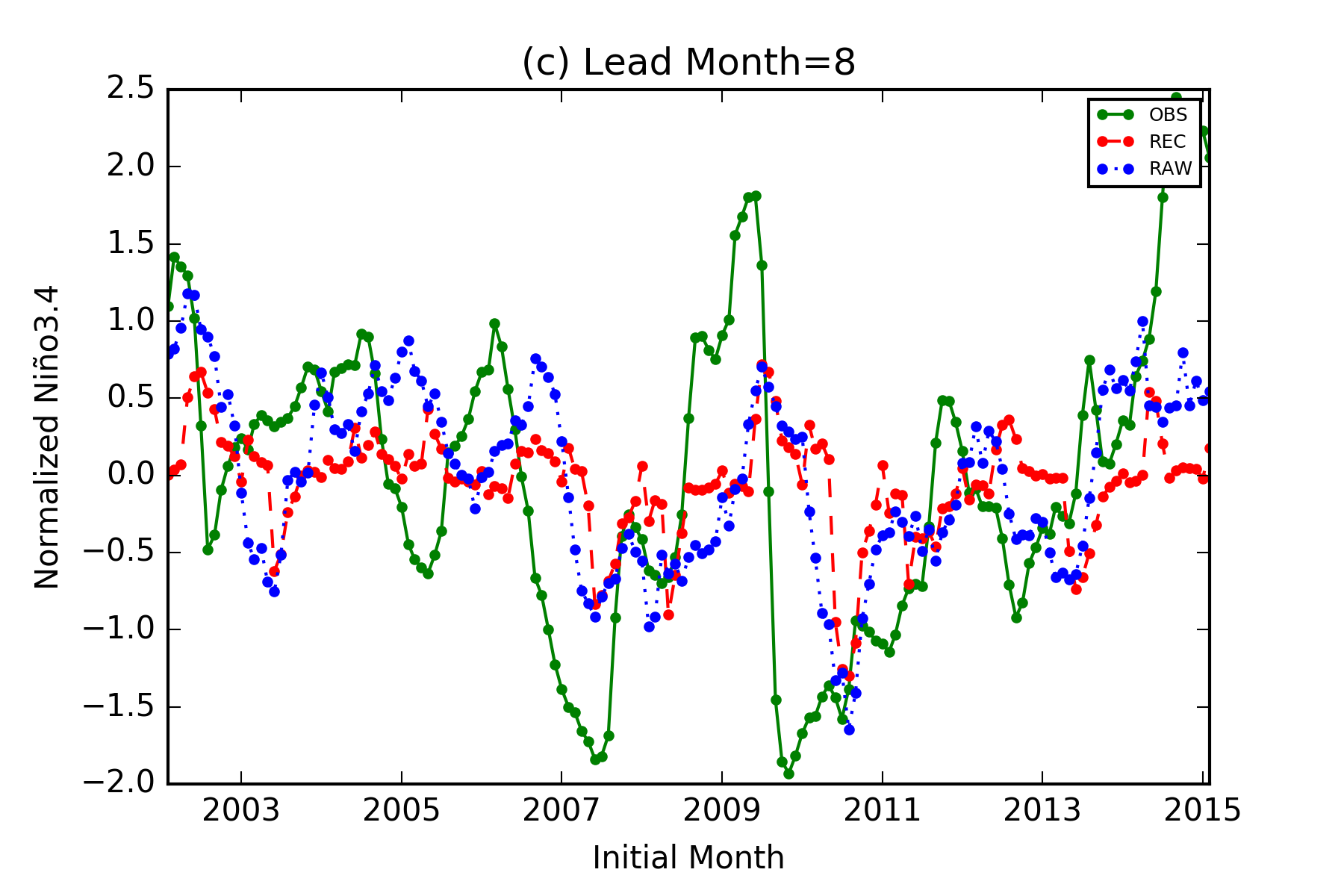


**Supplementary Fig. 7** Normalized Niño3.4 index in observation (green solid line), ensemble mean of raw prediction (blue dotted line), and reconstruction based on MSN EOF1 and EOF2 (red dashed line) for (a) zero-month, (b) four-month, and (c) eight-month lead predictions.

Here, we compare the normalized Niño3.4 index in the observation, reconstructed prediction, raw prediction for zero-month, four-month and eight-month lead, respectively (Supplementary Fig. 7). At shorter lead times, the normalized Niño3.4 index for the ensemble mean of raw predictions and for the reconstruction based on MSN EOF1 and EOF2 are quite similar and have small errors. However the differences increase at longer lead times. For eight-month lead, both the raw ensemble mean prediction and reconstruction prediction have difficulty capturing the extreme ENSO events.
